# SOX12 Facilitates Hepatocellular Carcinoma Progression and Metastasis through Promoting Regulatory T‐Cells Infiltration and Immunosuppression

**DOI:** 10.1002/advs.202310304

**Published:** 2024-07-29

**Authors:** Xiangyuan Luo, Wenjie Huang, Siwen Li, Mengyu Sun, Dian Hu, Junqing Jiang, Zerui Zhang, Yijun Wang, Yufei Wang, Jiaqian Zhang, Zhangfan Wu, Xiaoyu Ji, Danfei Liu, Xiaoping Chen, Bixiang Zhang, Huifang Liang, Yiwei Li, Bifeng Liu, Shuai Wang, Xiao Xu, Yongzhan Nie, Kaichun Wu, Daiming Fan, Limin Xia

**Affiliations:** ^1^ Department of Gastroenterology Institute of Liver and Gastrointestinal Diseases Hubei Key Laboratory of Hepato‐Pancreato‐Biliary Diseases Tongji Hospital of Tongji Medical College Huazhong University of Science and Technology Wuhan 430030 China; ^2^ Hepatic Surgery Center Hubei Key Laboratory of Hepato‐Pancreato‐Biliary Diseases Tongji Hospital of Tongji Medical College Huazhong University of Science and Technology Clinical Medicine Research Center for Hepatic Surgery of Hubei Province Key Laboratory of Organ Transplantation Ministry of Education and Ministry of Public Health Wuhan 430030 China; ^3^ State Key Laboratory of Holistic Integrative Management of Gastrointestinal Cancers and National Clinical Research Center for Digestive Diseases Xijing Hospital of Digestive Diseases Fourth Military Medical University Xi’ an 710032 China; ^4^ The Key Laboratory for Biomedical Photonics of MOE at Wuhan National Laboratory for Optoelectronics‐Hubei Bioinformatics and Molecular Imaging Key Laboratory Systems Biology Theme Department of Biomedical Engineering College of Life Science and Technology Huazhong University of Science and Technology Wuhan 430074 China; ^5^ Key Laboratory of Integrated Oncology and Intelligent Medicine of Zhejiang Province Department of Hepatobiliary and Pancreatic Surgery Affiliated Hangzhou First People's Hospital Zhejiang University School of Medicine Hangzhou 310006 China

**Keywords:** CCL22/CCR4, combined immunotherapy, PD‐L1, TGF‐β1/TGFβR1 signaling, Tregs

## Abstract

Despite the success of immunotherapy in treating hepatocellular carcinoma (HCC), HCC remains a severe threat to health. Here, a crucial transcription factor, SOX12, is revealed that induces the immunosuppression of liver tumor microenvironment. Overexpressing SOX12 in HCC syngeneic models increases intratumoral regulatory T‐cell (Treg) infiltration, decreases CD8^+^T‐cell infiltration, and hastens HCC metastasis. Hepatocyte‐specific SOX12 knockout attenuates DEN/CCl_4_‐induced HCC progression and metastasis, whereas hepatocyte‐specific SOX12 knock‐in accelerates these effects. Mechanistically, SOX12 transcriptionally activates C‐C motif chemokine ligand 22 (*CCL22*) expression to promote the recruitment and suppressive activity of Tregs. Moreover, SOX12 transcriptionally upregulates *CD274* expression to suppress CD8^+^T‐cell infiltration. Either knockdown of CCL22 or PD‐L1 dampens SOX12‐mediated HCC metastasis. Blocking of CC chemokine receptor 4 (CCR4), a receptor for CCL22, by inhibitor C‐021 or Treg‐specific knockout of CCR4 inhibits SOX12‐mediated HCC metastasis. Transforming growth factor‐β1 (TGF‐β1)/TGFβR1‐Smad2/3/4 is identified as a key upstream signaling for SOX12 overexpression in HCC cells. Combining C‐021 or TGFβR1 inhibitor galunisertib with anti‐PD‐L1 exhibits an enhanced antitumor effect in two HCC models. Collectively, the findings demonstrate that SOX12 contributes to HCC immunosuppression through the CCL22/CCR4‐Treg and PD‐L1‐CD8^+^T axes. Blocking of CCR4 or TGFβR1 improves the efficacy of anti‐PD‐L1 in SOX12‐mediated HCC.

## Graphical abstract

Transforming growth factor‐β1 (TGF‐β1)‐stimulated SOX12 overexpression remodels the immunosuppressive microenvironment of hepatocellular carcinoma (HCC), promoting tumor progression and metastasis. SOX12 boosts regulatory T cells (Tregs) through the C‐C motif chemokine ligand 22/CC chemokine receptor 4 (CCR4) axis and dampens CD8^+^T cells via PD‐L1 upregulation. Combining the CCR4 inhibitor C‐021 or TGF‐β1 receptor inhibitor galunisertib with anti‐PD‐L1 inhibits SOX12‐mediated effect.

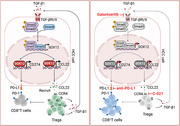

## 1 Introduction

Hepatocellular carcinoma (HCC) is a severe health burden globally.^[^ ^1^ ^]^ There have long been few treatment options for HCC, but immune checkpoint blockade (ICB) has made a significant breakthrough in recent years.^[^ ^2^ ^]^ Classic immune checkpoints such as programmed death 1 (PD‐1) are mainly expressed in immune cells, while its ligand programmed death ligand 1 (PD‐L1) is frequently expressed in tumor cells and other cells.^[^ ^3^ ^]^ In the tumor immune microenvironment (TIME), immune cells interact with tumor cells through paired immune checkpoints or other signals to develop a heterogenous and immunosuppressive ecosystem, facilitating tumor progression and metastasis.^[^ ^4^ ^]^ To date, ICB's low response rate and immunotoxicity have seriously hindered its application in HCC patients, underscoring the need for a complete understanding of how HCC cells interact with immune cells.

Regulatory T‐cells (Tregs) are a class of T‐cells characterized by expression of CD25 and forkhead box p3 (Foxp3), which maintain immune system homeostasis by negatively regulating immune response.^[^ ^5^ ^]^ Due to their immunosuppressive property, Tregs usually play a vital role in the formation of suppressive TIME. Relevant mechanisms include impairing effector T‐cells or secreting inhibitory inflammation factors (TGF‐β1, IL‐10, and IL‐35).^[^ ^6^ ^]^ Tregs infiltrate extensively in various tumors and promote their progression and metastasis by multiple mechanisms.^[^ ^7^ ^]^ Among them, CC chemokine receptor 4 (CCR4) and its ligands C─C motif chemokine ligand 22 (CCL22) are leading chemokines responsible for recruiting Tregs and promoting tumor immune escape.^[^ ^8^ ^]^ CCR4 is expressed in more than 90% of human Tregs, and CCL22 is also overexpressed in many human cancers.^[^ ^9^ ^]^ Several studies have emphasized the essentiality of Tregs in HCC progression and metastasis.^[^ ^10^, ^11^ ^]^ However, the molecular mechanism of the Tregs infiltration in HCC and the interaction between Tregs and HCC cells remains incompletely understood.

Sex determining region Y (SRY)‐related high‐mobility group (HMG) box (SOX) factors belong to an evolutionarily conserved family of transcription factors characterized by a DNA‐binding HMG box domain. The SOX family is essential in cell fate decisions involving numerous developmental processes.^[^ ^12^ ^]^ Once dysregulated, SOX proteins act as master regulators mediating the malignant progression of tumors.^[^ ^13^ ^]^ We previously demonstrated that SOX12, a member of subgroup C of the SOX family, is significantly upregulated in gastric cancer, colorectal cancer, and HCC and acts as an oncogene to facilitate their progression and metastasis.^[^ ^14^, ^15^, ^16^ ^]^ However, whether SOX12 participates in regulating the TIME of HCC remains unknown. Determining the regulatory relationship between SOX12 and the TIME of HCC is therefore essential for better understanding the complex mechanisms of immune cell‐HCC cell interactions.

Here, we identified that transforming growth factor‐β1 (TGF‐β1) upregulated SOX12 in HCC cells via the Smad2/3/4 pathway. SOX12 induced the imbalance between Tregs and effector T‐cells by transcriptionally upregulating the expression of *CCL22* and *CD274*, thus facilitating HCC progression and metastasis. Combination therapy of anti‐PD‐L1 and CCR4 inhibitor or TGFβR1 inhibitor impeded SOX12‐mediated HCC progression and metastasis.

## 2 Results

### 2.1 Overexpression of SOX12 in HCC Cells Facilitates Tregs Infiltration and HCC Metastasis in Immunocompetent Mice

**Figure 1:**
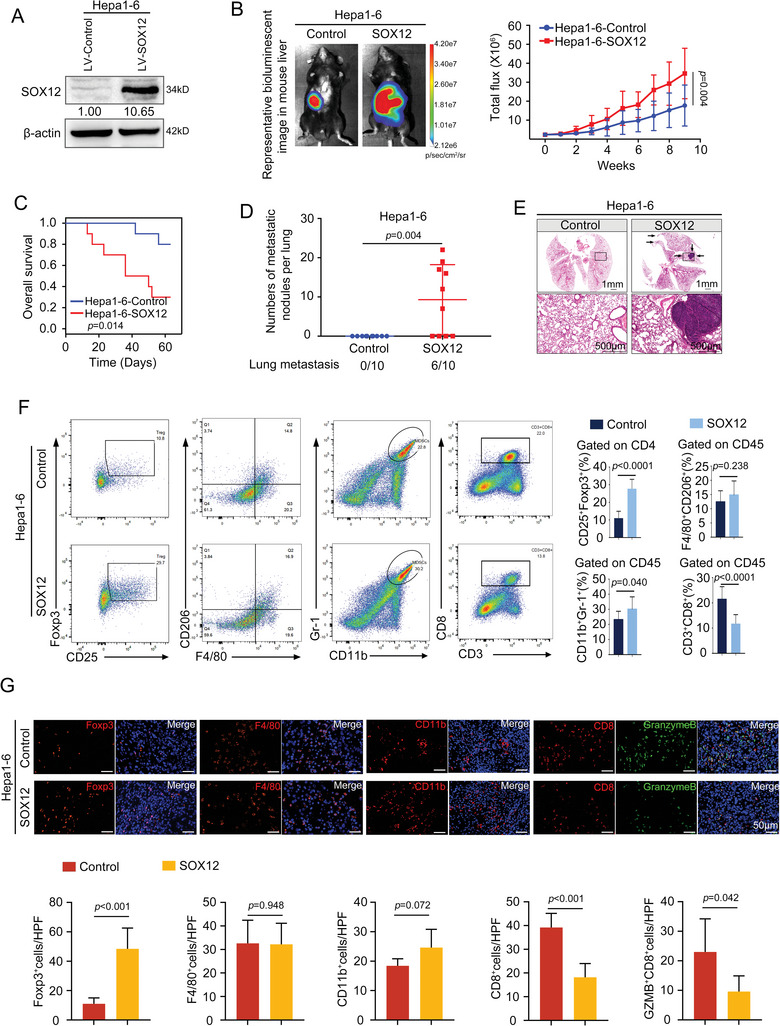
Overexpression of SOX12 in HCC cells facilitates Tregs infiltration and HCC metastasis. A) The efficiency of SOX12 overexpression was validated. B–G) Construction of intrahepatic orthotopic model bearing Hepa1‐6‐SOX12 cells (n = 10 per group). (B) The representative bioluminescent images and bioluminescence intensity of tumors, (C) overall survival, (D) lung metastatic nodule numbers, and (E) representative lung H&E staining were shown. (F–G) The intratumoral infiltration of CD25^+^Foxp3^+^Tregs, F4/80^+^CD206^+^TAMs, CD11b^+^Gr‐1^+^MDSCs, and CD3^+^CD8^+^T‐cells was analyzed by flow cytometry (F) and immunofluorescent staining (G). For (B), Two‐way ANOVA. For (C), Long‐rank test. For (D), (F), and (G), Unpaired *t*‐test. HPF, High power field; GZMB, Granzyme B.

We have previously reported that SOX12 is highly expressed in HCC and facilitates HCC metastasis in immunodeficient mice, suggesting that SOX12 functioned as an oncogene in HCC.^[^ ^16^ ^]^ However, whether SOX12 regulates the HCC immune microenvironment remains unknown. Bioinformatics analysis indicated that SOX12 expression was significantly associated with the infiltration of immunosuppressive cells in HCC, including myeloid‐derived suppressor cell (MDSC) and Treg (Figure S1, Supporting Information). Next, we established intrahepatic orthotopic implantation models using immunocompetent mice bearing SOX12‐overexpressed mouse HCC cells (Hepa1‐6‐SOX12 and H22‐SOX12) (**Figure** **1**A; Figure S2A, Supporting Information). Compared with the control group, mice in the SOX12 overexpression group exhibited worse survival and a significant elevation of lung metastasis rate (Figure 1B–E; Figure S2B–E, Supporting Information). Upregulation of SOX12 dramatically increased CD25^+^Foxp3^+^Tregs infiltration, observably decreased CD3^+^CD8^+^T‐cells infiltration, and moderately elevated CD11b^+^Gr‐1^+^MDSCs percentages (Figure 1F,G; Figure S2F–G, Supporting Information). There were no statistical differences in the proportions of F4/80^+^CD206^+^ tumor‐associated macrophages (TAMs) between the two groups (Figure 1F–G; Figure S2F,G, Supporting Information). These results suggested that SOX12 primarily affected the abundance of intratumoral Tregs and CD8^+^T‐cells of HCC.

### 2.2 Hepatocyte‐Specific SOX12 Knockout Attenuates DEN/CCl_4_‐Induced HCC Progression and Metastasis, Whereas Hepatocyte‐Specific SOX12 Knock‐In Accelerates This Process

**Figure 2:**
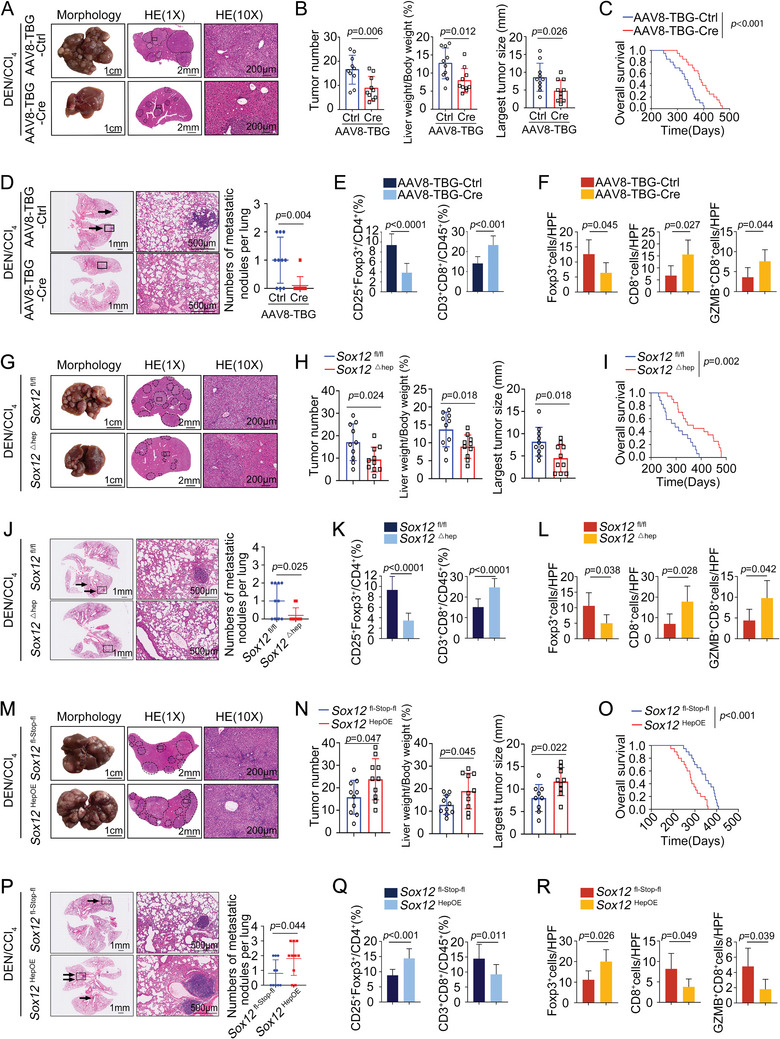
Hepatocyte‐specific SOX12 knockout attenuates DEN/CCl_4_‐induced HCC progression and metastasis, whereas hepatocyte‐specific SOX12 knock‐in accelerates this process. A) Typical appearances and H&E staining images of the liver from the DEN/CCl_4_‐treated *Sox12* ^fl/fl^ mice injected with the AAV8‐TBG‐Cre virus or control virus (n = 10 per group). B) The tumor numbers, liver‐to‐body ratios, and largest tumor size of the indicated mice. C) The overall survival of the indicated mice. D) The representative lung H&E staining and lung metastatic nodule numbers in the indicated mice. E,F) The intrahepatic infiltration of CD25^+^Foxp3^+^Tregs and CD3^+^CD8^+^T‐cells was analyzed by flow cytometry (E) and immunofluorescent staining (F). G) Typical appearances and H&E staining images of the livers from the DEN/CCl_4_‐treated *Sox12* ^△hep^ mice at 34 weeks of age (n = 10 per group). H) The tumor numbers, liver‐to‐body ratios, and largest tumor size of the indicated mice. I) The overall survival of the indicated mice. J) The representative lung H&E staining and lung metastatic nodule numbers were shown. K,L) The intrahepatic infiltration of CD25^+^Foxp3^+^Tregs and CD3^+^CD8^+^T‐cells was analyzed by flow cytometry (K) and immunofluorescent staining (L). M) Typical appearances and H&E staining images of the livers from the DEN/CCl_4_‐treated *Sox12* ^HepOE^ mice at 34 weeks of age (n = 10 per group; n = 9 per group for detecting the largest tumor size). N) The tumor numbers, liver‐to‐body ratios, and largest tumor size of the indicated mice were examined. O) The overall survival of the indicated mice. P) The representative lung H&E staining and lung metastatic nodule numbers were shown. Q,R) The intrahepatic infiltration of CD25^+^Foxp3^+^Tregs and CD3^+^CD8^+^T‐cells was analyzed by flow cytometry (Q) and immunofluorescent staining (R). For (B), (D–F), (H), (J–L), (N), (P–R), Unpaired *t*‐test. For (C), (I), and (O), Long‐rank test. HPF, High power field; GZMB, Granzyme B.

Adequate diethylnitrosamine (DEN)/carbon tetrachloride (CCl_4_) treatment in mice can spontaneously induce the initiation and development of HCC characterized by chronic liver inflammation, which is better suited to study the inflammatory and immune microenvironment of HCC.^[^ ^17^ ^]^ Following the administration of DEN/CCl_4_, we observed pronounced liver tumor lesions and specific lung metastatic nodules in mice at 34 weeks (Figure S3A–C, Supporting Information). Immunofluorescent staining showed that the intratumoral infiltration of Tregs was increased, whereas the infiltration of effector T‐cells was decreased (Figure S3D, Supporting Information). Moreover, in comparison to the control group, the expression of SOX12 was progressively upregulated in the DEN/CCl_4_ group (Figure S3E, Supporting Information). This suggested that SOX12 might be involved in the initiation and progression of HCC. To investigate the potential role of SOX12 in the DEN/CCl_4_‐induced HCC progression and metastasis, we constructed a hepatocyte‐specific SOX12 knockout model utilizing the adeno‐associated virus‐serotype 8 (AAV8)‐thyroxine‐binding globulin promoter (TBG)‐Cre recombinase system (Figure S4A,B, Supporting Information). When SOX12 was eliminated from hepatocytes, the DEN/CCl_4_‐induced liver tumor and mouse death were significantly alleviated, as indicated by reduced tumor numbers, decreased ratio of liver weight to body weight, diminishing largest tumor size, and prolonged overall survival (**Figure** **2**A–C). Deletion of SOX12 reduced the levels of alanine transaminase (ALT) and aspartate transaminase (AST), suggesting a relative recovery in liver function (Figure S4C, Supporting Information). Hepatocyte‐specific SOX12 knockout mice also exhibited fewer lung metastatic nodules (Figure 2D). Flow cytometry and immunofluorescent staining indicated that hepatocellular deficiency of SOX12 dramatically inhibited DEN/CCl_4_‐induced intratumoral Tregs accumulation and recovered CD8^+^T‐cells infiltration (Figure 2E,F; Figure S4D,E, Supporting Information). To further validate, we created another hepatocyte‐specific SOX12 knockout (*Sox12* ^△hep^) mice by crossing *albumin‐Cre* (*Alb‐Cre*) mice and *Sox12* ^flox/flox^ mice (Figure S4F–I, Supporting Information). In this model, the results we obtained were consistent with those indicated in the hepatocyte‐specific SOX12 knockout model established using the AAV8‐TBG‐Cre system, demonstrating a remission of DEN/CCl_4_‐induced tumor burden, mouse mortality, lung metastasis, and impaired liver function (Figure 2G–J; Figure S4J, Supporting Information). Moreover, the DEN/CCl_4_‐induced dysregulation of Tregs and CD8^+^T‐cells was improved in *Sox12* ^△hep^ mice (Figure 2K,L; Figure S4K,L, Supporting Information). By isolating Tregs from liver tumors, we observed that the mRNA levels of several functional cytokines (IL‐10, IL‐35, TGF‐β1) in Tregs from *Sox12* ^△hep^ tumor were down‐regulated (Figure S4M, Supporting Information). These results suggested that the hepatocyte‐specific knockout of SOX12 diminished the progression and metastasis of HCC induced by DEN/CCl_4_, while also alleviating the abnormal accumulation and functional secretion of intratumoral Tregs and increasing the infiltration of effector T‐cells.

Subsequently, we generated hepatocyte‐specific SOX12 knock‐in mice (*Sox12* ^HepOE^; Figure S5A–E, Supporting Information). Overexpression of SOX12 in hepatocytes remarkably deteriorated the DEN/CCl_4_‐caused progression and metastasis of HCC (Figure 2M–P; Figure S5F, Supporting Information). Meanwhile, the knock‐in of SOX12 deepened the DEN/CCl_4_‐induced liver immunosuppression (Figure 2Q,R; Figure S5G–I, Supporting Information). These studies suggested that SOX12 was pivotal in remodeling the immunosuppressive microenvironment and facilitating HCC progression and metastasis.

### 2.3 *CCL22* and *CD274* Are Two Key Transcriptional Targets of SOX12 in HCC Cells

**Figure 3:**
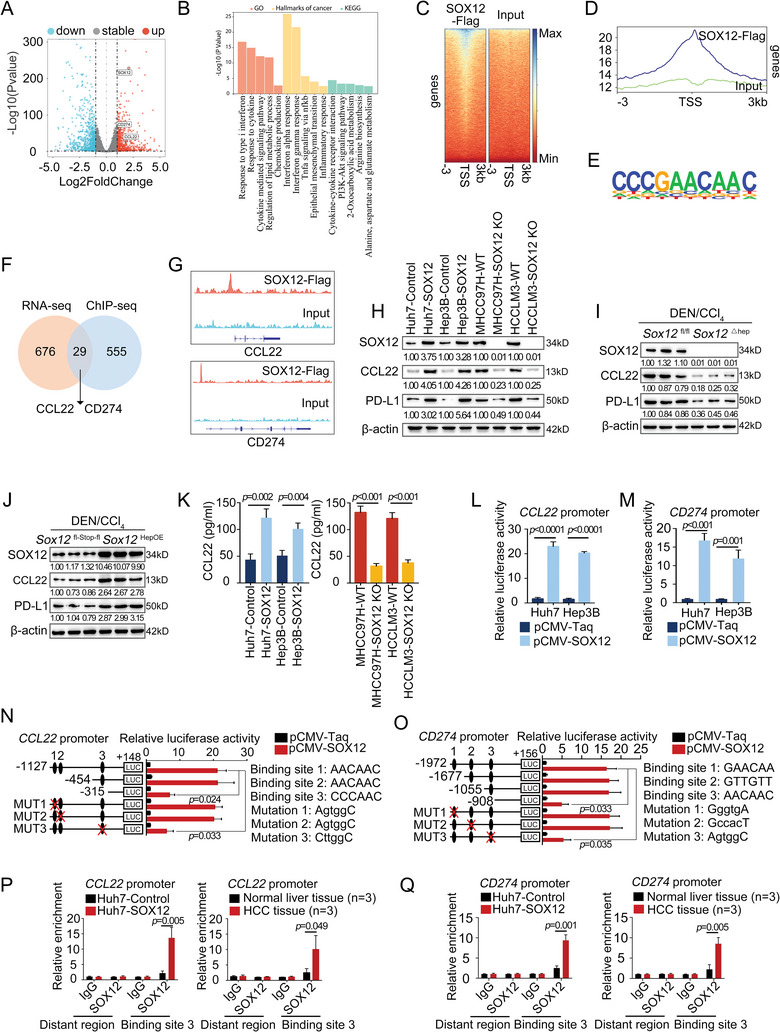
*CCL22* and *CD274* are two key transcriptional targets of SOX12 in HCC cells. A) Volcano plot mapped from the RNA‐sequencing results analyzing the differentially expressed genes (DEGs) between Huh7‐SOX12 and Huh7‐Control cells. B) The enrichment analysis of the DEGs of RNA‐sequencing. C,D) Heatmaps and density plot of ChIP‐sequencing signals for SOX12‐Flag and input in the transcription start site (TSS) region. E) SOX12‐Flag ChIP‐seq‐derived de novo motif logo. F) Venn diagram of overlapping upregulated genes from RNA‐sequencing data with ChIP‐sequencing data. G) Gene track view of SOX12‐Flag ChIP‐seq tags in the *CCL22* promoter and *CD274* promoter. H) The expression of CCL22 and PD‐L1 in HCC cells with SOX12 overexpression or knockout. I,J) The expression of CCL22 and PD‐L1 in the liver tumors of DEN/CCl_4_‐treated *Sox12* ^△hep^ mice (I) and *Sox12* ^HepOE^ mice (J). K) ELISA detected the supernatant level of CCL22. L,M) The activities of the *CCL22* promoter (L) and *CD274* promoter (M) in HCC cells were detected by luciferase assay. N,O) The activities of the serially truncated or mutated *CCL22* promoter (N) and *CD274* promoter (O) in Huh7 cells were detected by luciferase assay. P,Q) The binding of SOX12 on the *CCL22* promoter (P) and *CD274* promoter (Q) in Huh7 cells and HCC tissues were detected by ChIP assay. For (K–M), Unpaired *t*‐test. For (N–Q), Two‐way ANOVA.

We wondered how SOX12 regulates the infiltration of these immune cells in HCC. Herein, we performed RNA‐sequencing on total RNA extracted from Huh7 cells overexpressed with SOX12 (Huh7‐SOX12) and control cells (Huh7‐Control). By comparing the Huh7‐SOX12 group and the Huh7‐Control group, we screened 1628 differentially expressed genes (DEGs), including 705 upregulated genes and 923 down‐regulated genes (Fold Change > 2, p.adj < 0.05) (**Figure** **3**A; Table S1, Supporting Information). Enrichment analysis suggested that SOX12 was involved in the chemokine and cytokine‐mediated signaling pathways, inflammatory responses, interferon responses, epithelial‐mesenchymal transition, and various metabolic processes (Figure 3B). To further identify direct targets of SOX12, chromatin immunoprecipitation (ChIP)‐sequencing in the Huh7‐SOX12‐Flag cells was performed (Figure 3C,D). We obtained a 10‐nucleotide sequence motif closely associated with SOX12 binding (Figure 3E) and identified many SOX12‐binding genes (Table S2, Supporting Information). By overlapping our RNA‐sequencing data and ChIP‐sequencing data, 29 candidate targets were selected (Figure 3F). Due to the notable increase in Tregs observed in mice liver tumors with overexpressed SOX12, we directed our research toward exploring the underlying mechanisms. From the pool of 29 potential targets of SOX12, our attention was drawn to *CCL22*, a pivotal chemokine known for its role in the recruitment of Tregs through binding to its specific receptor, CCR4 (Figure 3G).^[^ ^7^ ^]^ Therefore, we hypothesized that CCL22 might mediate the accumulation of intratumoral Tregs induced by SOX12. Moreover, we discovered that *CD274*, responsible for encoding the classic immune checkpoint PD‐L1 and being one of the most successful immunotherapeutic targets, is also a potential target of SOX12 (Figure 3G). This aroused our interest. We subsequently confirmed the positive regulatory effect of SOX12 on CCL22 and PD‐L1 expression in human HCC cells and mice *Sox12* ^△hep^ and *Sox12* ^HepOE^ HCC models (Figure 3H–K; Figure S6A,B, Supporting Information). Besides, SOX12 enhanced the luciferase activities of the *CCL22* and *CD274* promoters (Figure 3L,M). Using bioinformatics analysis, we identified three putative SOX12‐binding sites on the *CCL22* promoter (Figure S7, Supporting Information) and constructed corresponding reporters with truncations or site‐directed mutagenesis of the *CCL22* promoter. Deletion of the *CCL22* promoter region ranging from −454 to −315 bp observably interfered with the SOX12‐induced luciferase activation (Figure 3N). Mutation of a putative binding site within the −454 to −315 bp region of the *CCL22* promoter removed the SOX12‐induced *CCL22* promoter activity (Figure 3N). Meanwhile, we also demonstrated that SOX12 transactivated *CD274* by binding to the binding site 3 sequence (AACAAC) in the −1055 to −908 bp region of the *CD274* promoter (Figure 3O; Figure S8, Supporting Information). ChIP assay verified the direct binding of SOX12 to the *CCL22* and *CD274* promoters (Figure 3P,Q). Altogether, our results revealed that *CCL22* and *CD274* were two direct transactivation targets of SOX12 in HCC.

### 2.4 SOX12 Facilitates HCC Metastasis Through Upregulating CCL22 and PD‐L1 Expression

**Figure 4:**
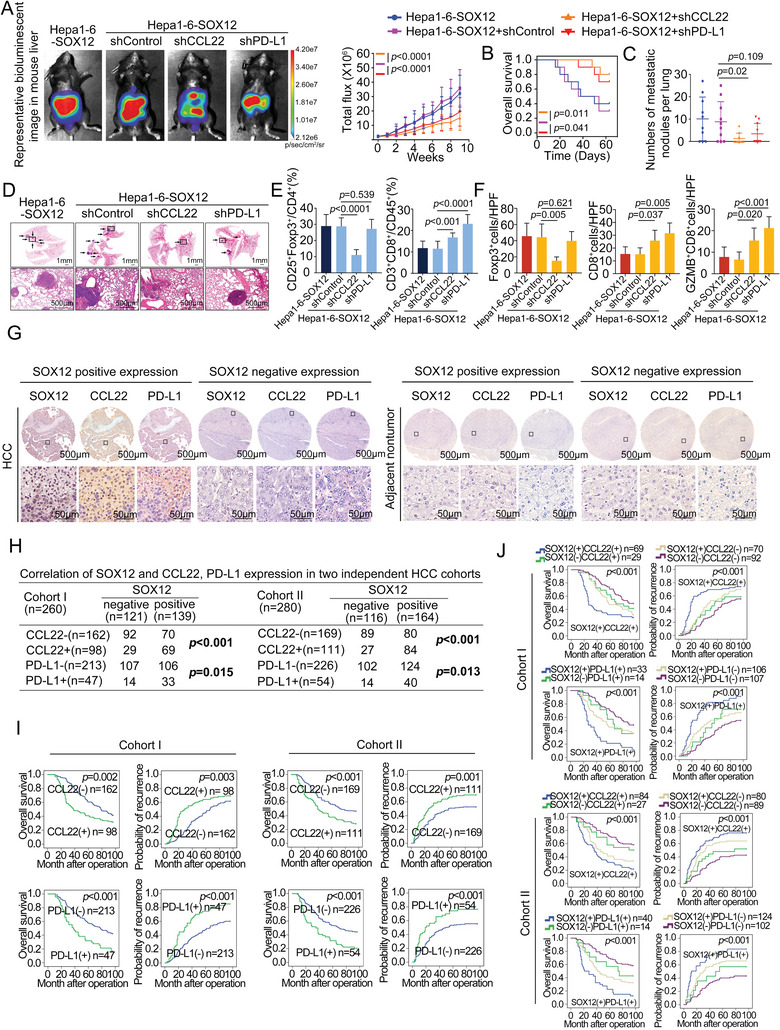
SOX12 facilitates HCC metastasis through upregulating CCL22 and PD‐L1 expression. A–F) Construction of intrahepatic orthotopic model bearing Hepa1‐6‐SOX12‐shCCL22 or Hepa1‐6‐SOX12‐shPD‐L1 cells (n = 10 per group). (A) The representative bioluminescent images and bioluminescence intensity of tumors, (B) overall survival, (C) lung metastatic nodule numbers, and (D) representative lung H&E staining were shown. (E,F) The intratumoral infiltration of CD25^+^Foxp3^+^Tregs and CD3^+^CD8^+^T‐cells was analyzed by flow cytometry (E) and immunofluorescent staining (F). G) Representative IHC images of SOX12, CCL22, and PD‐L1 expression in adjacent nontumor tissues and HCC tissues in human tissue microarray. The scale bars display 500 µm (low magnification) and 50 µm (high magnification). H) Association analysis of SOX12 and CCL22, PD‐L1 expression in two cohorts. I) Association of the recurrence rate or overall survival time and the expression of CCL22 or PD‐L1 in two cohorts was analyzed by Kaplan–Meier analysis. J) Association of the recurrence rate or overall survival time and the co‐expression of SOX12/CCL22 or SOX12/PD‐L1 in two cohorts was analyzed by Kaplan–Meier analysis. For (A), Two‐way ANOVA. For (B), (I), and (J), Long‐rank test. For (C), (E), and (F), Unpaired *t*‐test. For (H), Chi‐squared test. HPF, High power field; GZMB, Granzyme B.

We noted a gradual increase in the levels of CCL22 and PD‐L1 in mice following DEN/CCl_4_ treatment, suggesting the involvement of these proteins in the HCC progression and metastasis. (Figure S6C, Supporting Information). For this purpose, we silenced the expression of CCL22 or PD‐L1 in Hepa1‐6‐SOX12 cells (Figure S6D, Supporting Information) and then generated orthotopic implantation models. Results showed that the knockdown of either CCL22 or PD‐L1 ameliorated the survival burden of mice and reduced HCC metastasis caused by SOX12 overexpression (**Figure** **4**A–D). Down‐regulation of CCL22 or PD‐L1 also alleviated the SOX12‐mediated inhibition of CD8^+^T‐cells infiltration (Figure 4E,F; Figure S6E,F, Supporting Information). Notably, down‐regulation of CCL22 significantly suppressed the SOX12‐induced increase of Tregs infiltration, but down‐regulation of PD‐L1 did not affect Tregs infiltration (Figure 4E,F; Figure S6E,F, Supporting Information). These results suggested that SOX12 upregulated CCL22 and PD‐L1 expression to facilitate HCC metastasis.

In addition, by immunohistochemical (IHC) staining of two independent HCC cohorts, we demonstrated that SOX12 expression was positively associated with the CCL22 and PD‐L1 expression (Figure 4G,H). HCC patients with positive expression of CCL22 or PD‐L1 were positively correlated with more aggressive tumor behaviors (Tables S3 and S4, Supporting Information) and worse prognosis (Figure 4I). HCC patients with simultaneous expression of SOX12/CCL22 or SOX12/PD‐L1 displayed a poorer prognosis among all (Figure 4J).

### 2.5 Intratumoral Tregs Recruited by the SOX12‐CCL22‐CCR4 Axis Suppress CD8^+^T‐Cells and Mediate SOX12‐Induced HCC Metastasis

**Figure 5:**
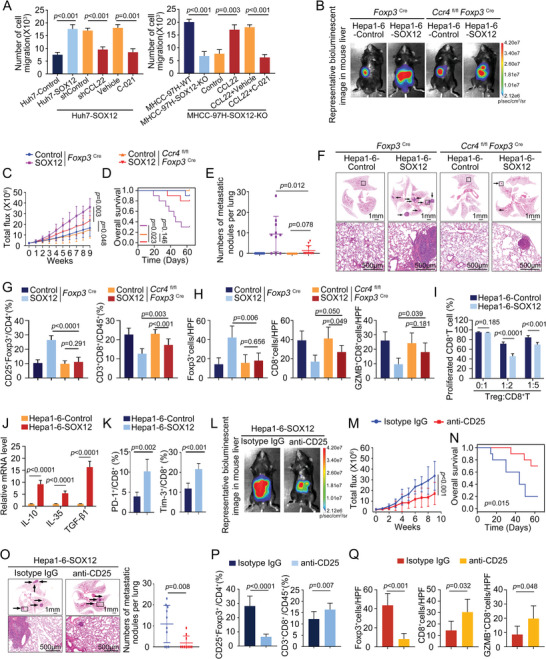
Intratumoral Tregs recruited by the SOX12‐CCL22‐CCR4 axis suppress CD8^+^T‐cells and mediate SOX12‐induced HCC metastasis. A) The number of Treg migration in co‐culture of human PBMCs‐derived Tregs with the supernatant of HCC cells under different treatments for 24 h, including SOX12 overexpression followed by CCL22 knockdown or C‐021 treatment (1 µM), and SOX12 knockout followed by CCL22 overexpression and C‐021 treatment (1 µM). B–H) The intrahepatic orthotopic models bearing Hepa1‐6‐SOX12 cells were constructed based on *Ccr4* ^fl/fl^ *Foxp3* ^Cre^ mice (n = 10 per group). (B,C) The representative bioluminescent images and bioluminescence intensity of tumors, (D) overall survival, (E) lung metastatic nodule numbers, and (F) representative lung H&E staining were shown. (G,H) The intratumoral infiltration of CD25^+^Foxp3^+^Tregs and CD3^+^CD8^+^T‐cells was analyzed by flow cytometry (G) and immunofluorescent staining (H). I) The proliferation of CFSE‐labeled CD8^+^T‐cells was determined by flow cytometry. J) The mRNA levels of IL‐10, IL‐35, and TGF‐β1 in Tregs from the orthotopic tumor‐bearing Hepa1‐6‐Control cells or bearing Hepa1‐6‐SOX12 cells. K) Flow cytometry was utilized to analyze the proportions of PD‐1^+^/CD8^+^T‐cells and Tim‐3^+^/CD8^+^T‐cells in the liver tumors of mice after the orthotopic injection of specific HCC cells for 2 weeks. (n = 5 per group). L–Q) Administration of anti‐CD25 to Hepa1‐6‐SOX12 cells‐established intrahepatic orthotopic model (n = 10 per group). (L,M) The representative bioluminescent images and bioluminescence intensity of tumors, (N) overall survival, (O) lung metastatic nodule numbers and representative lung H&E staining were shown. (P,Q) The intratumoral infiltration of CD25^+^Foxp3^+^Tregs and CD3^+^CD8^+^T‐cells was analyzed by flow cytometry (P) and immunofluorescent staining (Q). For (C) and (M), Two‐way ANOVA. For (D) and (N), Long‐rank test. For (A), (E), (G–K), (O), (P,Q), Unpaired *t*‐test.

Given the vital role of CCL22 and its receptor CCR4 on Tregs recruitment and tumor progression,^[^ ^18^ ^]^ we hypothesized that CCL22‐CCR4 axis was involved in SOX12‐induced Tregs enrichment. In vitro migration assay showed that upregulation of SOX12 in Huh7 cells promoted Tregs migration, whereas knockout of SOX12 in MHCC‐97H cells suppressed Tregs migration (**Figure** **5**A). Either down‐regulation of CCL22 by lentivirus or blocking of CCR4 by its inhibitor C‐021 eliminated SOX12‐induced Tregs migration (Figure 5A). Inversely, overexpression of CCL22 reversed the suppression of Tregs migration caused by SOX12 knockout, and subsequent administration of C‐021 further inhibited the promoting effect of CCL22 overexpression on Treg migration (Figure 5A). In our in vivo experiments, we observed that HCC cells served as the principal source of CCL22 when SOX12 was overexpressed in Hepa1‐6 cells (Figure S9A,B, Supporting Information). This observation suggested that SOX12 primarily regulated intracellular CCL22 within HCC cells. Subsequently, treatment of orthotopic implantation mice with C‐021 prolonged their survival and inhibited SOX12‐induced lung metastasis (Figure S10A–D, Supporting Information). Administration of C‐021 also obstructed SOX12‐mediated Tregs enrichment and CD8^+^T‐cells reduction (Figure S10E,F, Supporting Information). To further investigate whether the upregulation of CCL22 by SOX12 enhances the infiltration of intratumoral Tregs by directly binding to the receptor CCR4 specifically expressed on Tregs, we generated Treg‐specific CCR4 knockout mice (*Ccr4* ^fl/fl^ *Foxp3* ^Cre^) (Figure S11A–C, Supporting Information) and constructed an orthotopic implantation model bearing Hepa1‐6‐SOX12 cells. Elimination of CCR4 in Tregs alleviated SOX12‐mediated HCC metastasis and improved the survival rate of these mice (Figure 5B–F). Flow cytometry and immunofluorescent staining revealed almost complete suppression of SOX12‐induced Tregs accumulation and a corresponding increase in effector CD8^+^T‐cells infiltration in the *Ccr4* ^fl/fl^ *Foxp3* ^Cre^ mice compared with the control group of *Foxp3* ^Cre^ mice (Figure 5G,H; Figure S11D,E, Supporting Information).

Responder T‐cells are the primary targets for Tregs to exert their immunosuppressive function.^[^ ^18^ ^]^ To detect the immunosuppressive activity of Tregs recruited by SOX12, we performed a T‐cell suppression assay (Figure S11F, Supporting Information). Results showed that Tregs isolated from tumors of syngeneic orthotopic transplantation mice bearing Hepa1‐6‐SOX12 cells exhibited enhanced suppressive ability to the proliferation of CD8^+^T‐cells compared with Tregs from control mice (Figure 5I; Figure S11G, Supporting Information). Moreover, the mRNA levels of IL‐10, IL‐35, and TGF‐β1 in Tregs from SOX12‐overexpressed tumors were higher than those in control tumors (Figure 5J). These results suggested that SOX12 also influenced the immunosuppressive function of Tregs. In a previous study, it was noted that inhibition of CCR4 led to a reduction in the proportion of PD‐1^+^CD8^+^T‐cell in HCC, which represents a subset of exhausted T‐cells.^[^ ^11^ ^]^ In addition, PD‐L1 is a key molecule that promotes T‐cell exhaustion.^[^ ^19^ ^]^ We hypothesized whether SOX12 plays a role in regulating T‐cell exhaustion. Indeed, we observed higher percentages of PD‐1^+^CD8^+^T‐cells and Tim‐3^+^CD8^+^T‐cells, indicative of T‐cell exhaustion, in Hepa1‐6‐SOX12 tumors compared to Hepa1‐6‐Control tumors in the orthotopic model (Figure 5K; Figure S11H, Supporting Information). RT‐qPCR analysis revealed elevated mRNA levels of PD‐1 and Tim‐3 in CD8^+^T‐cells isolated from Hepa1‐6‐SOX12 tumors in comparison to those from Hepa1‐6‐Control tumors (Figure S11I, Supporting Information). Consequently, overexpression of SOX12 in HCC cells triggered T‐cell exhaustion.

Subsequently, we assessed the role of SOX12‐mediated Tregs accumulation in HCC metastasis. Depletion of Tregs by anti‐CD25 antibodies remarkably restrained SOX12‐induced lung metastasis (Figure 5L–Q; Figure S11J–K, Supporting Information). Collectively, these studies suggested that overexpression of SOX12 in HCC cells promoted Tregs recruitment through the CCL22‐CCR4 axis and enhanced Tregs function, which suppressed CD8^+^T‐cells and facilitated HCC metastasis.

### 2.6 TGF‐β1 Upregulates SOX12 Expression Through the Smad2/Smad3/Smad4 Signaling Pathway

**Figure 6:**
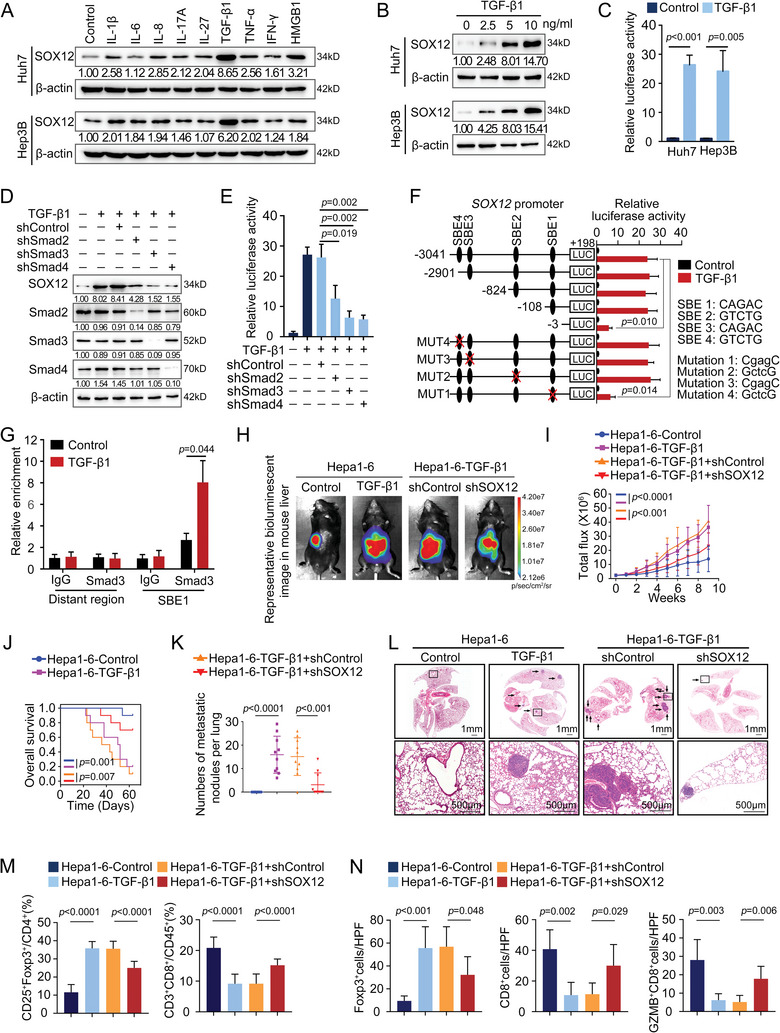
TGF‐β1‐Smad2/Smad3/Smad4 upregulates SOX12 expression and promotes HCC metastasis. A) The expression of SOX12 in HCC cells with treatment of cytokines for 24 h. B) The expression of SOX12 in HCC cells treated with different concentrations of TGF‐β1 for 24 h was detected. C) The activity of *SOX12* promoter in TGF‐β1‐treated HCC cells was detected by luciferase assay. D) The levels of SOX12 and Smad2/3/4 in TGF‐β1‐treated Huh7 cells with Smad2/3/4 knockdown were detected. E) The activity of *SOX12* promoter in TGF‐β1‐stimulated Huh7 cells with Smad2/3/4 knockdown was tested by luciferase assay. F) The activity of serially truncated or mutated *SOX12* promoter in TGF‐β1‐stimulated Huh7 cells was detected by luciferase assay. G) The binding of Smad3 on *SOX12* promotor in TGF‐β1‐stimulated Huh7 cells was tested by ChIP assay. H–N) Establishment of intrahepatic orthotopic models bearing Hepa1‐6‐TGF‐β1 or Hepa1‐6‐TGF‐β1‐shSOX12 cells (n = 10 per group). (H,I) The representative bioluminescent images and bioluminescence intensity of tumors, (J) overall survival, (K) lung metastatic nodule numbers, and (L) representative lung H&E staining were shown. (M,N) The intratumoral infiltration of CD25^+^Foxp3^+^Tregs and CD3^+^CD8^+^T‐cells was analyzed by flow cytometry (M) and immunofluorescent staining (N). For (C), (E), (K), (M,N), Unpaired *t*‐test. For (F–I), Two‐way ANOVA. For (J), Long‐rank test. HPF, High power field; GZMB, Granzyme B.

The precise mechanisms controlling SOX12 expression remain not fully straightforward. Inflammation is a feature of HCC and presents in the whole process from hepatitis to HCC. Inflammatory cytokines play pivotal roles in HCC initiation, progression, and metastasis.^[^ ^20^ ^]^ Therefore, we hypothesized whether inflammatory cytokines regulate SOX12 expression. For this purpose, HCC cells with low endogenous SOX12 expression were treated with various inflammatory cytokines (IL‐1β, IL‐6, IL‐8, IL‐17A, IL‐27, TGF‐β1, TNF‐α, IFN‐γ, and HMGB1) and the results showed that TGF‐β1 significantly induced SOX12 upregulation in a concentration‐dependent manner (**Figure** **6A,B**; Figure S12A,B, Supporting Information). Next, we treated TGF‐β1‐stimulated Huh7 cells with inhibitors targeting various TGF‐β1‐regulated signaling pathways.^[^ ^21^ ^]^ Interestingly, only the Smad3 inhibitor relieved the TGF‐β1‐induced SOX12 overexpression (Figure S12C, Supporting Information). Besides, TGF‐β1 sharply increased the activity of *SOX12* promoter in HCC cells (Figure 6C). Silencing of Smad2, Smad3, or Smad4 in Huh7 cells, which are classical transcriptional regulators of TGF‐β1 signaling, inhibited the upregulation and transcription activation of SOX12 induced by TGF‐β1 (Figure 6D,E; Figure S12D, Supporting Information). Truncation of the *SOX12* promoter from −108 to −3 bp region or mutation of the Smad binding element 1 (SBE1) sequence within this region suppressed TGF‐β1‐induced *SOX12* promoter activation (Figure 6F; Figure S13, Supporting Information). ChIP assay further verified the binding of the Smad2/3/4 complex to the *SOX12* promoter (Figure 6G). Hence, TGF‐β1‐Smad2/3/4‐induced transcriptional activation of *SOX12* was one of the essential mechanisms for the abnormal high‐expression of SOX12 in HCC.

### 2.7 SOX12 Is Essential for TGF‐β1‐Induced HCC Metastasis

Treg is one of the sources of TGF‐β1 and plays its immunosuppressive role partially through producing TGF‐β1.^[^ ^5^ ^]^ We detected a higher level of TGF‐β1 in the liver of the orthotopic model bearing Hepa1‐6‐SOX12 cells compared with control mice models (Figure S12E, Supporting Information). To investigate whether Treg is the cell repository that generates TGF‐β1 in the HCC immune microenvironment, we co‐cultured different treatments of Hepa1‐6 cells with Tregs isolated from tumors of syngeneic orthotopic mice bearing Hepa1‐6‐SOX12 cells (Figure S12F, Supporting Information). Hepa1‐6 cells co‐cultured with Tregs expressed significantly greater SOX12 levels (Figure S12G, Supporting Information). This upregulated expression of SOX12 was subsequently suppressed by a TGF‐β1 neutralizing antibody or the TGF‐β1 receptor I (TGFβR1) inhibitor galunisertib, indicating that Tregs‐secreted TGF‐β1 stimulated SOX12 overexpression in HCC (Figure S12G, Supporting Information). Furthermore, the orthotopic implantation model showed that the down‐regulation of SOX12 partly impeded the TGF‐β1‐induced HCC metastasis (Figure 6H–L; Figure S12H, Supporting Information). Concurrently, silencing of SOX12 attenuated the increase of Tregs abundance and the decrease of effector CD8^+^T‐cells abundance caused by TGF‐β1 (Figure 6M,N; Figure S12I,J, Supporting Information). These results indicated that SOX12 was essential for TGF‐β1‐induced immunosuppression and HCC metastasis.

### 2.8 SOX12 Expression Is Positively Correlated With Intratumoral Tregs Infiltration But Negatively Correlated With CD8^+^T‐Cells Infiltration in Human HCC Tissues

To further investigate the correlation between SOX12 expression and immune cell infiltration in human HCC, IHC staining was performed on two retrospective HCC cohorts (Figure S14A, Supporting Information). SOX12 expression was positively associated with the Foxp3 (representative of Treg) and CD11b (representative of MDSC, neutrophils, monocyte, and macrophage)^[^ ^22^, ^23^ ^]^ expression but negatively related to the CD8 (representative of CD8^+^T‐cell) expression (Figure S14B, Supporting Information). There was no statistical significance in the correlation between SOX12 expression and CD163 (representative of macrophage and monocyte)^[^ ^24^ ^]^ expression (Figure S14B, Supporting Information). Patients with positive SOX12, Foxp3, CD11b, and CD163 expression or negative CD8 expression showed tumor‐aggressive tendency (Tables S5–S9, Supporting Information) and poor prognosis (Figure S14C; Table S10, Supporting Information). HCC patients with SOX12^+^Foxp3^+^, SOX12^+^CD11b^+,^ or SOX12^+^CD8^−^ exhibited the worst prognosis (Figure S14D, Supporting Information).

### 2.9 Combination of CCR4 Inhibitor C‐021 or TGFβR1 Inhibitor Galunisertib With Anti‐PD‐L1 Hinders SOX12‐Mediated HCC Progression and Metastasis

**Figure 7:**
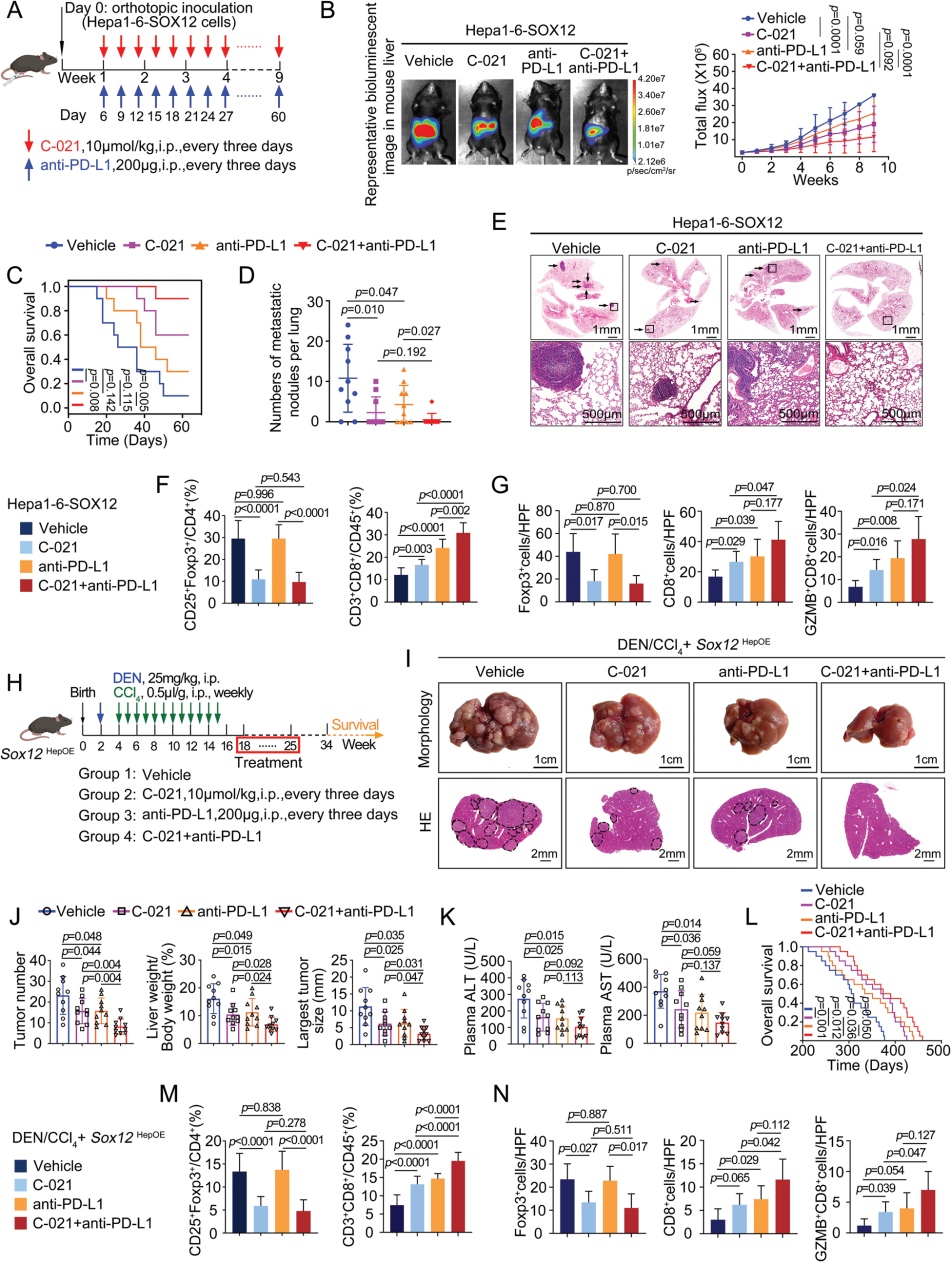
Combination of CCR4 inhibitor C‐021 with anti‐PD‐L1 hinders SOX12‐mediated HCC progression and metastasis. A) Schematic workflow of establishing intrahepatic orthotopic model and treating with vehicle, C‐021, and/or anti‐PD‐L1 (n = 10 per group). B) The representative bioluminescent images and bioluminescence intensity of tumors, C) overall survival, D) lung metastatic nodule numbers, and E) representative lung H&E staining were shown. F,G) The intratumoral infiltration of CD25^+^Foxp3^+^Tregs and CD3^+^CD8^+^T‐cells was analyzed by flow cytometry (F) and immunofluorescent staining (G). H) Schematic workflow of evaluating the antitumor effect of C‐021 in combination with anti‐PD‐L1 on the DEN/CCl_4_‐treated *Sox12* ^HepOE^ model (n = 10 per group). I) Typical appearances and H&E staining images of the livers from the DEN/CCl_4_‐treated *Sox12* ^HepOE^ mice with the administration of C‐021 and/or anti‐PD‐L1 (n = 10 per group). J) The tumor numbers, liver‐to‐body ratios, and largest tumor size of the indicated mice. K) The levels of plasma ALT and AST in the indicated mice. L) The overall survival of mice. M,N) The intrahepatic infiltration of CD25^+^Foxp3^+^Tregs and CD3^+^CD8^+^T‐cells was analyzed by flow cytometry (M) and immunofluorescent staining (N). For (B), Two‐way ANOVA. For (C) and (L), Long‐rank test. For (D), (F), (G), (J), (K), (M), and (N), Unpaired *t*‐test. HPF, High power field; GZMB, Granzyme B.

Considering the crucial effect of SOX12‐CCL22/CCR4 and SOX12‐PD‐L1 axis on the TIME remodeling and the HCC progression and metastasis, we evaluated the antitumor effect of combining CCR4 inhibitor (C‐021) with anti‐PD‐L1 in HCC models. Intrahepatic orthotopic implantation model showed that administration of either C‐021 or anti‐PD‐L1 decelerated tumor growth, improved mice survival, and relatively controlled lung metastasis compared with the vehicle group. However, the combination treatment of C‐021 and anti‐PD‐L1 dramatically inhibited these SOX12‐mediated tumor‐promoting roles compared with the two single‐drug groups (**Figure** **7**A–E; Figure S15A–D, Supporting Information). As illustrated in Figure 7F,G, Figures S15E,F and S16A,B (Supporting Information), combination treatment also significantly boosted the proportion of CD8^+^T‐cells compared with the two monotherapy groups. Besides, we assessed the efficacy of C‐021 combined with anti‐PD‐L1 in a DEN/CCl_4_‐treated *Sox12* ^HepOE^ mouse model (Figure 7H). Compared with the two monotherapy groups, mice in the combination therapy group showed a significant reduction of tumor burden, improvement of liver function, and restoration of the immunosuppressive microenvironment (Figure 7I–N; Figure S16C,D, Supporting Information).

**Figure 8:**
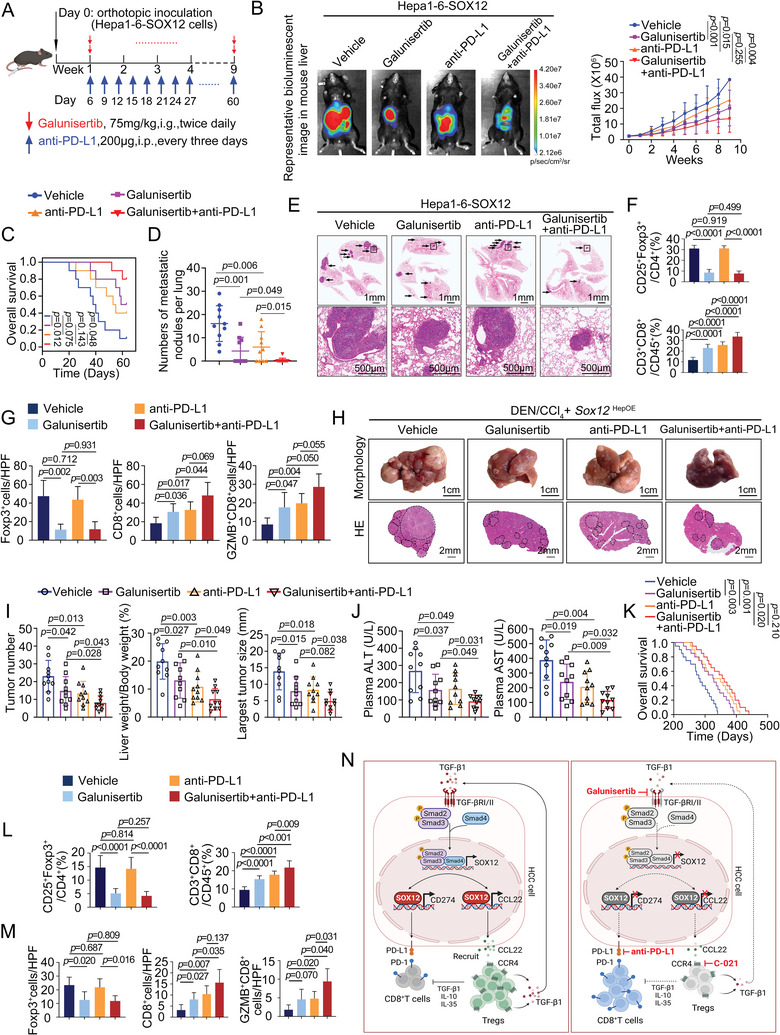
Combination of TGFβR1 inhibitor galunisertib with anti‐PD‐L1 suppresses SOX12‐mediated HCC progression and metastasis. A) Schematic workflow of establishing intrahepatic orthotopic model bearing Hepa1‐6‐SOX12 cells and treating with vehicle, galunisertib, and/or anti‐PD‐L1 (n = 10 per group). B) The representative bioluminescent images and bioluminescence intensity of tumors, C) overall survival, D) lung metastatic nodule numbers, and E) representative lung H&E staining were shown. F,G) The intratumoral infiltration of CD25^+^Foxp3^+^Tregs and CD3^+^CD8^+^T‐cells was analyzed by flow cytometry (F) and immunofluorescent staining (G). H) Typical appearances and H&E staining images of the livers from the DEN/CCl_4_‐treated *Sox12* ^HepOE^ mice with the administration of galunisertib and/or anti‐PD‐L1 (n = 10 per group). I) The tumor numbers, liver‐to‐body ratios, and largest tumor size of the indicated mice. J) The levels of plasma ALT and AST in the indicated mice. K) The overall survival of mice. L,M) The intrahepatic infiltration of CD25^+^Foxp3^+^Tregs and CD3^+^CD8^+^T‐cells was analyzed by flow cytometry (L) and immunofluorescent staining (M). N) Schematic illustration of liver immune microenvironment remodeling by the TGF‐β1‐SOX12‐CCL22/PD‐L1 axis. For (B), Two‐way ANOVA. For (C) and (K), Long‐rank test. For (D), (F), (G), (I), (J), (L), and (M), Unpaired *t*‐test. HPF, High power field; GZMB, Granzyme B.

In addition, previous studies demonstrated the promising antitumor effect of the TGFβR1 inhibitor galunisertib in combination with anti‐PD‐L1 in multiple tumors.^[^ ^25^, ^26^ ^]^ However, their effect on HCC remains unclear. For this purpose, we treated the orthotopic HCC models and the DEN/CCl_4_‐treated *Sox12* ^HepOE^ model with galunisertib and anti‐PD‐L1 (**Figure** **8**A; Figure S17A, Supporting Information). The combination therapy group was more effective in suppressing the progression and metastasis of tumor than either galunisertib or anti‐PD‐L1 monotherapy and had better survival rates (Figure 8B–E,H–K; Figure S18A–D, Supporting Information). Similar suppression of Tregs was observed in the galunisertib group and combination group. However, combination therapy dramatically boosted the infiltration of effector CD8^+^T‐cells compared with the other two monotherapies (Figure 8F,G,L,M; Figures S17B–E and S18E–F). These results suggested that combining C‐021 or galunisertib with anti‐PD‐L1 exhibited a more prominent ability to inhibit SOX12‐mediated HCC progression and metastasis.

Collectively, our study depicted a novel regulatory mechanism of transcription factor SOX12 on the HCC immune microenvironment and proposed two potential combination immunotherapy strategies for HCC (Figure 8N).

## 3 Discussion

Only 15–20% of HCC patients respond to single‐drug ICB therapy, calling for a comprehensive exploration of the effect and mechanism underlying the suppressive TIME formation and proposing new therapeutic strategies for HCC.^[^ ^2^ ^]^ Tregs are suppressive immune cells that often accumulate abnormally in tumors and antagonize the antitumor immunity of effector T‐cells.^[^ ^27^ ^]^ HCC is characterized by intratumoral Tregs enrichment, which is critical for HCC progression.^[^ ^20^ ^]^ Based on our previous findings that SOX12 functions as an oncogene in HCC^[^ ^16^ ^]^ and evidence that SOX12 in CD4^+^T‐cells suppresses T helper cell 2 (Th2) differentiation and promotes Treg development,^[^ ^28^, ^29^ ^]^ this study further investigated the effect of SOX12 on TIME of HCC. Here, we uncovered that SOX12 promoted Tregs infiltration, decreased CD8^+^T‐cells infiltration, and facilitated HCC progression and metastasis in orthotopic HCC models and DEN/CCl_4_‐treated *Sox12* ^△hep^ and *Sox12* ^HepOE^ models. SOX12 expression was positively associated with the Tregs infiltration, whereas negatively associated with CD8^+^T‐cells infiltration by analyzing IHC scores. Therefore, SOX12 was involved in the abnormal infiltration of Tregs and CD8^+^T‐cells in HCC.

Chemokines and cytokines are important factors leading to the accumulation and activation of Treg in HCC, such as CCL22 and IL‐33.^[^ ^30^, ^31^ ^]^ Among them, CCL22 and its receptor CCR4 are crucial for cell migration and homing of Tregs and Th2 cells, thus promoting HCC development and metastasis.^[^ ^30^, ^32^ ^]^ Inhibiting the CCL22/CCR4 axis impairs intratumoral Treg infiltration without affecting conventional CD4^+^T‐cells, underscoring the specific chemotactic role of CCL22/CCR4 in intratumoral Tregs.^[^ ^18^ ^]^ Here, we identified CCL22 as a key target of SOX12 through RNA‐sequencing and ChIP‐sequencing. Subsequent investigation demonstrated that SOX12 directly transactivated *CCL22* in HCC cells. Knockdown of CCL22 or administration of CCR4 inhibitor C‐021 suppressed SOX12‐mediated Tregs recruitment and HCC metastasis. Treg‐specific CCR4 knockout attenuated SOX12‐induced HCC metastasis. Elimination of Tregs by anti‐CD25 antibody also repressed SOX12‐mediated effects. A previous study revealed that CCR4‐positive Tregs in HCC exhibit enhanced suppressive function to CD8^+^T‐cells.^[^ ^11^ ^]^ Consistently, our T‐cell suppression assay and RT‐qPCR assay showed that Tregs from SOX12‐overexpressed tumors had reinforced immunosuppressive activity and expressed higher effector cytokines. These results indicated that overexpression of SOX12 in HCC cells promoted the secretion of CCL22 and the recruitment and function of Tregs, thereby facilitating HCC progression and metastasis. We depicted a new transcriptional mechanism that mediates communication between HCC cells and Tregs.

The recruitment of Tregs through the SOX12‐CCL22/CCR4 axis significantly contributed to the reduced infiltration of CD8^+^T‐cells in HCC. We were interested in exploring additional molecular mechanisms by which SOX12 regulates CD8^+^T‐cells. Our analysis of high‐throughput sequencing data revealed PD‐L1 as one of the candidate targets of SOX12. PD‐L1, a ligand for the co‐inhibitory receptor PD‐1, is expressed on various cells, especially tumor cells.^[^ ^3^ ^]^ The PD‐L1/PD‐1 interaction inhibits T‐cell proliferation, survival, and cytokine secretion as well as promotes T‐cell exhaustion, making it a key player in T‐cell suppression and a prominent target for antitumor immunotherapy, including in HCC.^[^ ^3^, ^33^ ^]^ Transcription factors are one of the major regulators of PD‐L1 abnormal expression in tumors. For example, FOXM1, IRF1, and STAT3 have been shown to bind to the *CD274* promoter and regulate the PD‐L1 transcription in tumor cells.^[^ ^34^, ^35^, ^36^ ^]^ Our study found that SOX12 upregulated the PD‐L1 expression by directly activating *CD274* promoter transcription in HCC cells. Down‐regulation of PD‐L1 impeded SOX12‐mediated HCC metastasis and relatively restored the unbalanced CD8^+^T‐cells. Furthermore, overexpression of SOX12 increased the intratumoral infiltration of PD‐1^+^CD8^+^T‐cells and Tim‐3^+^CD8^+^T‐cells, suggesting that SOX12 induced T‐cell exhaustion. The expression of PD‐L1 and CCL22 was positively correlated with SOX12 expression in HCC tissues. Therefore, we uncovered a novel molecular regulatory mechanism of PD‐L1 upregulation in HCC and provided a potential biomarker to guide the application of anti‐PD‐L1 in HCC therapy.

TGF‐β1 signaling is a crucial decider of cell fate in the development process and homeostasis maintenance. Despite the contradictory effect of TGF‐β1 on HCC initiation and progression, TGF‐β1 promotes HCC progression and metastasis by creating an inflammatory, fibrotic, and immunosuppressive environment that has been well documented.^[^ ^37^ ^]^ For Tregs, TGF‐β1 is indispensable, as it controls the differentiation and inhibitory function of Tregs.^[^ ^21^ ^]^ Our study screened that TGF‐β1‐Smad2/3/4 signaling was an upstream activator of *SOX12* transcription in HCC cells. Knockdown of SOX12 attenuated TGF‐β1‐induced HCC metastasis, Tregs enrichment, and CD8^+^T‐cells decrease, suggesting that SOX12 was a function mediator of TGF‐β1. Tregs play their suppressive role in part through secreting TGF‐β1. We also found that TGF‐β1 was partly derived from Tregs in the SOX12‐mediated HCC immune microenvironment. Therefore, we identified a positive feedback regulatory mechanism of communication between HCC cells and Tregs via the TGF‐β1‐SOX12‐CCL22/CCR4‐Tregs axis.

Combination immunotherapy is an optimal strategy to improve single‐agent ICB therapy's efficacy and response rate, and multiple new combinations of ICBs combined with different drugs have been proposed to treat tumors.^[^ ^38^ ^]^ Tregs are attractive immuno‐oncology targets, and strategies targeting Tregs are being evaluated in several studies. However, systemic Tregs elimination while rebooting antitumor immunity can also lead to autoimmune diseases since the Tregs in healthy tissues can also be impaired.^[^ ^39^ ^]^ Therefore, a strategy to specifically target intratumoral Tregs and bypass healthy tissues is needed. CCR4 is an intratumoral Treg‐specific marker. In HCC, ICB combined with C‐021, a small molecule inhibitor targeting CCR4, or neutralizing antibody against CCR4, exhibits a significant antitumor effect.^[^ ^8^, ^11^ ^]^ Our intrahepatic orthotopic HCC models and DEN/CCl_4_‐treated *Sox12* ^HepOE^ model demonstrated a significant advantage of C‐021 combined with anti‐PD‐L1 treatment in restoring antitumor immunity and suppressing the SOX12‐mediated HCC progression and metastasis. Additionally, TGF‐β1 signaling can induce PD‐L1 expression in cancer cells, and co‐treatment of TGF‐β1 inhibition and PD‐L1 blockade enhances antitumor immunity.^[^ ^40^, ^41^ ^]^ Thus, we evaluated the efficacy of galunisertib, a small molecule inhibitor targeting TGFβR1 kinase, in combination with anti‐PD‐L1 in these HCC models. We observed an apparent tumor repression and CD8^+^T‐cells increase in the combination treatment groups. Consequently, our study revealed two combination immunotherapy strategies for HCC using SOX12 as a biomarker in preclinical models.

Recently, our group identified another member of SOX family, SOX18, as a key driver in reshaping the suppressive TIME of HCC by regulating the infiltration of Tregs and TAMs.^[^ ^42^ ^]^ While SOX12 and SOX18 share functional similarities, they exhibit distinct characteristics with significant implications. First, these two transcription factors regulate distinct chemokines, influencing immune cells with varying types and functions, thereby shaping unique TIME in HCC. Specifically, SOX12 transactivated *CCL22*, which resulted in the accumulation and enhanced activity of CCR4^+^Tregs within the tumor, whereas SOX18 up‐regulated CXCL12 expression, attracting CXCR4^+^TAMs and CXCR4^+^Tregs into the tumor. Despite both SOX12 and SOX18 attracting Tregs, these Tregs consist of distinct subpopulations with diverse molecular characteristics and functions, resulting in a substantial divergence in the TIME induced by SOX12 compared to that induced by SOX18. Second, these two studies present distinct immunotherapy approaches tailored for HCC based on different molecular characteristics. This study investigated the efficacy of a combination therapy involving the CCR4 inhibitor C‐021 and anti‐PD‐L1 in mouse models of HCC with SOX12 overexpression. In contrast, another study evaluated the CXCR4 inhibitor AMD3100 in combination with anti‐PD‐L1 treatment in HCC models overexpressing SOX18. While both studies explored combination therapies with anti‐PD‐L1, they targeted distinct HCC patient subpopulations. Given the prevalent overexpression of SOX12 in HCC patients,^[^ ^16^ ^]^ our study provided a potential therapeutic strategy specifically for this subgroup, highlighting its significant clinical implications. Finally, the two studies demonstrated significant antitumor effects of inhibiting the TGF‐β1 receptor combined with anti‐PD‐L1 in HCC models with SOX12 or SOX18 overexpression. This indicates that the combination therapy of TGF‐β1 signaling inhibition and anti‐PD‐L1 is effective in treating HCC with various molecular characteristics.

In conclusion, we illustrated that the TGF‐β1/Smad2/Smad3/Smad4 signal induced SOX12 overexpression in HCC cells. Upregulated SOX12 remodeled the immunosuppressive microenvironment by promoting CCL22/CCR4‐mediated Treg recruitment and functional enhancement and inducing PD‐L1‐mediated immune evasion, facilitating HCC progression and metastasis. Our findings provided new insights into the communication among HCC cells, Tregs, and CD8^+^T‐cells. We also evaluated the optimistic antitumor effects of two combination immunotherapy strategies for SOX12‐mediated HCC and provided evidence for improving HCC immunotherapy.

## 4 Experimental Section

### Patients and Follow‐Up

This study was approved by the Ethics Committee of Tongji Hospital of Tongji Medical College, Huazhong University of Science and Technology (TJ‐IRB20220713). Cohort I included 260 adult patients with HCC who underwent curative resection between 2003 and 2005 at the Tongji Hospital of Tongji Medical College (Wuhan, China). Cohort II included 280 adult patients with HCC who underwent curative resection between 2006 and 2008 at the Tongji Hospital of Tongji Medical College (Wuhan, China). A preoperative clinical diagnosis of HCC was based on the diagnostic criteria of the American Association for the Study of Liver Diseases. The inclusion criteria were as follows: (a) distinctive pathologic diagnosis; (b) no preoperative anticancer treatment or distant metastases; (c) curative liver resection; and (d) complete clinicopathologic and follow‐up data. The differentiation statuses were graded according to the method of Edmondson and Steine. The pTNM classification for HCC was based on The American Joint Committee on Cancer/International Union Against Cancer staging system (6th edition, 2002). Follow‐up data were summarized at the end of December 2013 (Cohort I) and December 2016 (Cohort II, range 4–96 months), respectively. The patients were evaluated every 2–3 months during the first 2 years and every 3–6 months thereafter. All follow‐up examinations were performed by physicians who were blinded to the study. During each check‐up, the patients were monitored for tumor recurrence by measuring the serum AFP levels and by performing abdominal ultrasound examinations. Computed tomography and/or magnetic resonance imaging examination was performed every 3–6 months, together with a chest radiographic examination. The diagnostic criteria for HCC recurrence were the same as the preoperative criteria. The time to recurrence and overall survival were the primary endpoints. The time to recurrence was calculated from the date of resection to the date of a diagnosis with tumor recurrence. The overall survival was calculated from the date of resection to the date of death or of the last follow‐up. The study adhered to the principles outlined in the Declaration of Helsinki. Each patient provided written informed consent.

### Mice Models and Treatments

The C57BL/6J and BALB/C mice used in this study were male aged 6–7 weeks unless specified otherwise. All animals were housed under specific pathogen‐free and standard conditions and fed ad libitum. In addition, all experiments were approved by the ethics committee of Tongji Hospital of Tongji Medical College, Huazhong University of Science and Technology (TJH‐202109018).

A clear description of the orthotopic implantation model has been made in the previous study.^[^ ^43^ ^]^

To establish the DEN/CCl_4_‐induced HCC model, 2‐week‐old male C57BL/6J mice were intraperitoneally injected with 25 mg k^−1^g of DEN (Sigma‐Aldrich, MO). After 2 weeks, these mice were intraperitoneally injected into 0.5 µl g^−1^ of CCl_4_ (Sigma–Aldrich, MO) weekly until mice aged 15 weeks. Mice were sacrificed for observation and analysis at 34 weeks. Observation time was not limited for mice used for survival analysis.

To obtain a hepatocyte‐specific SOX12 knockout (*Sox12* ^△hep^) model, the *Alb‐Cre* mice were mated with *Sox12* ^flox/flox^ mice. The *Sox12* ^flox/flox^ mice were generated by inserting two *loxP* sites within the exon 1 of *Sox12* using a CRISPR/Cas‐mediated genome engineering system. In addition, another hepatocyte‐specific SOX12 knockout mice was constructed by intravenous injection of AAV8‐TBG‐Cre into *Sox12* ^flox/flox^ mice at the age of 6 weeks. To generate a hepatocyte‐specific SOX12 knock‐in (*Sox12* ^HepOE^) model, the *Alb‐Cre* mice was crossed with *RosaSox12* mice. The *RosaSox12* mice were constructed by cloning the *Rosa26‐pCAG‐loxp‐STOP‐loxp‐mSox12‐pA* cassette into intron 1 of *Rosa* in mice utilizing the CRISPR/Cas‐mediated genome engineering system. To generate Treg‐specific CCR4 knockout mice (*Ccr4* ^fl/fl^ *Foxp3* ^Cre^), the *Foxp3* ^Cre^ mice was crossed with *Ccr4* ^flox/flox^ mice. The *Ccr4* ^flox/flox^ mice were generated by inserting two *loxP* sites within the exon 2 of *Ccr4* using a CRISPR/Cas‐mediated genome engineering system. All these tool mice were C57BL/6J background. The genome‐PCR primers were listed in Table S11 (Supporting Information).

For Treg deletion, each mouse was intraperitoneally injected with anti‐CD25 monoclonal antibody (Bio X cell) 500 µg twice a week for 9 weeks. For drug treatment, C‐021 (Tocris Bioscience) was injected intraperitoneally at 10 µmol kg^−1^ every 3 days for in vivo experiments. Anti‐PD‐L1 (Bio X Cell) was injected intraperitoneally at 200 µg every 3 days. Galunisertib/LY2157299 (Selleck and Eli Lilly) was administered orally to mice at 75 mg k^−1^g twice daily.

### Chromatin Immunoprecipitation Assay (ChIP)

Cells were immersed in 1% formaldehyde for 10 min at 37 °C to stimulate cross‐linking. Then, glycine was used to quench the formaldehyde after cross‐linking to stop formaldehyde fixation. After washing with PBS, the cells were resuspended in lysis buffer (1 mM PMSF, 1% SDS, 10 mM EDTA, and 50 mM Tris (pH 8.1)—total volume 300 µl). Sonication was then performed to produce fragmented DNA. A slurry of protein G‐Sepharose and herring sperm DNA (Sigma–Aldrich) was used to clear the supernatant. The recovered supernatant was then subjected to a 2‐h incubation period with specific antibodies or an isotype control IgG in the presence of protein G‐Sepharose beads and herring sperm DNA, followed by antibody denaturation with 1% SDS in lysis buffer. Precipitated DNA was extracted from the beads by immersing them in a 1.1 M NaHCO_3_ solution and 1% SDS solution at 65 °C for 6 h. Immunoprecipitated DNA was retrieved from the beads by immersion in 1% SDS and a 1.1 M NaHCO_3_ solution at 65 °C for 6 h. The DNA was then purified using a PCR Purification Kit (QIAGEN, USA). The primers were shown in Table S11 (Supporting Information).

For ChIP assays of tissues, cells were first separated from fresh frozen HCC tissues and normal liver tissues collected after surgical resection. In detail, surgically extracted tumor tissues were first washed with 1×cold PBS, 5 min, three times and added to a medium supplemented with antibiotic and antifungal agents. Use a clean razor blade to cut a piece of tissue (around 5mm^3^) into small pieces (typically 1mm^3^ or smaller). Then, digestion of the tissues with DNase I (20 mg mL^−1^; Sigma–Aldrich) and collagenase (1.5 mg mL^−1^; Sigma–Aldrich) and placed on a table concentrator, 37 °C, for 1 h. At the end of the hour, the dissociated cells were filtered through 100‐µm‐pore filters rinsed with fresh media. The 1×red cell lysis was added to the tissues and incubated for 5 min to lysis the red blood cell, followed by another rinse. The dissociated cells were crosslinked using 1% formaldehyde for 10 min at 37 °C. After cell lysis, the DNA was fragmented by sonication. ChIP grade antibody or IgG (negative control) was used to immunoprecipitate the fragment DNA. Then, RT‐qPCR was used to amplify the corresponding binding site on the promoters.

### Chromatin Immunoprecipitation Sequencing (ChIP‐Sequencing) and Data Analysis

ChIP using anti‐Flag (F1804, Millipore Sigma) was performed as described above. Sequencing service was provided by Bioyi Biotechnology Co., Ltd., Wuhan, China. The method of data analysis has been introduced in the previous work.^[^ ^44^ ^]^ In brief, FASTQ reads were mapped to the human genome hg38 with Bowtie2 with default filtering. Resulted SAM files were converted to BAM files through Samtools. BAM files were sorted and indexed with Samtools. Binding peaks were called using MACS2 with default settings and further annotated by ChIPSeeker. HOMER was used for the motif analysis. Then, genome views were achieved with IGV.

### Statistical Analysis

All values were recorded as the mean ± standard deviation (s.d.). All experiments were repeated three or more independent biological replicates. Statistical significance between the means of two groups was determined using Student's *t*‐tests (normal distribution), Mann–Whitney U tests (abnormal distribution), or Wilcoxon signed rank test (matched pairs). The statistics of the means of multiple groups were performed using one‐ or two‐way ANOVA. The cumulative recurrence and survival curves were shown by the Kaplan–Meier method and the statistical significance were determined by log‐rank test. Multivariate analysis was performed by Cox regression analysis. Correlations were performed by using chi‐squared test. Statistical analysis was justified as appropriate among all figures. P values < 0.05 were considered to be statistically significant. Statistical values were calculated with SPSS software (Version 20.0) or GraphPad Prism 8.0 software.

## Conflict of Interest

The authors declare no conflict of interest.

## Author Contributions

X.L., W.H., and S.L. contributed equally to this work. Methodology was done by X.L., Y.W., Y.W., X.J., D.L., and L.X. Validation was done by X.L., S.L., M.S., D.H., and J.J. Formal analysis was done by X.L., S.L., M.S., and Z.Z. Investigation was done by X.L., S.L., M.S., D.H., J.J., Y.W., Y.W., J.Z., Z.W., X.J., and D.L. Resource was contributed by W.H. and L.X. Data curation was done by M.S. and Z.Z. Visualization was done by X.L., M.S., and Z.Z. Funding acquisition was done by W.H. and L.X. Supervision was done by W.H., S.W., X.X., Y.N., K.W., D.F., and L.X. Writing—original draft was done by X.L. Writing—review & editing was done by W.H., X.C., B.Z., H.L., Y.L., B.L., Y.N., K.W., D.F., and L.X. Conceptualization was done by L.X.

## Supporting information

Supporting Information

## Data Availability

The data that support the findings of this study are available from the corresponding author upon reasonable request.

## References

[1] H. Sung , J. Ferlay , R. L. Siegel , M. Laversanne , I. Soerjomataram , A. Jemal , F. Bray , CA Cancer J. Clin. 2021, 71, 209. 33538338 10.3322/caac.21660

[2] J. M. Llovet , F. Castet , M. Heikenwalder , M. K. Maini , V. Mazzaferro , D. J. Pinato , E. Pikarsky , A. X. Zhu , R. S. Finn , Nat. Rev. Clin. Oncol. 2022, 19, 151. 34764464 10.1038/s41571-021-00573-2

[3] C. Sun , R. Mezzadra , T. N. Schumacher , Immunity 2018, 48, 434. 29562194 10.1016/j.immuni.2018.03.014 PMC7116507

[4] T. F. Gajewski , H. Schreiber , Y. X. Fu , Nat. Immunol. 2013, 14, 1014. 24048123 10.1038/ni.2703 PMC4118725

[5] S. Z. Josefowicz , L. F. Lu , A. Y. Rudensky , Annu. Rev. Immunol. 2012, 30, 531. 22224781 10.1146/annurev.immunol.25.022106.141623 PMC6066374

[6] X. Liu , W. Mo , J. Ye , L. Li , Y. Zhang , E. C. Hsueh , D. F. Hoft , G. Peng , Nat. Commun. 2018, 9, 249. 29339767 10.1038/s41467-017-02689-5 PMC5770447

[7] Y. Togashi , K. Shitara , H. Nishikawa , Nat. Rev. Clin. Oncol. 2019, 16, 356. 30705439 10.1038/s41571-019-0175-7

[8] O. Yoshie , Cancers 2021, 13, 5542.34771703

[9] L. A. Marshall , S. Marubayashi , A. Jorapur , S. Jacobson , M. Zibinsky , O. Robles , D. X. Hu , J. J. Jackson , D. Pookot , J. Sanchez , M. Brovarney , A. Wadsworth , D. Chian , D. Wustrow , P. D. Kassner , G. Cutler , B. Wong , D. G. Brockstedt , O. Talay , J. Immunother. Cancer 2020, 8, e000764. 33243932 10.1136/jitc-2020-000764 PMC7692993

[10] H. Wang , H. Zhang , Y. Wang , Z. J. Brown , Y. Xia , Z. Huang , C. Shen , Z. Hu , J. Beane , E. A. Ansa‐Addo , H. Huang , D. Tian , A. Tsung , J. Hepatol. 2021, 75, 1271. 34363921 10.1016/j.jhep.2021.07.032 PMC12888775

[11] Y. Gao , M. You , J. Fu , M. Tian , X. Zhong , C. Du , Z. Hong , Z. Zhu , J. Liu , G. J. Markowitz , F. S. Wang , P. Yang , J. Hepatol. 2022, 76, 148. 34689996 10.1016/j.jhep.2021.08.029

[12] Y. Kamachi , H. Kondoh , Development 2013, 140, 4129. 24086078 10.1242/dev.091793

[13] D. Grimm , J. Bauer , P. Wise , M. Kruger , U. Simonsen , M. Wehland , M. Infanger , T. J. Corydon , Semin. Cancer Biol. 2020, 67, 122. 30914279 10.1016/j.semcancer.2019.03.004

[14] F. Du , W. Feng , S. Chen , S. Wu , T. Cao , T. Yuan , D. Tian , Y. Nie , K. Wu , D. Fan , L. Xia , Cancer Lett. 2019, 452, 103. 30922917 10.1016/j.canlet.2019.03.035

[15] F. Du , J. Chen , H. Liu , Y. Cai , T. Cao , W. Han , X. Yi , M. Qian , D. Tian , Y. Nie , K. Wu , D. Fan , L. Xia , Cell Death Dis. 2019, 10, 239. 30858360 10.1038/s41419-019-1481-9 PMC6412063

[16] W. Huang , Z. Chen , X. Shang , D. Tian , D. Wang , K. Wu , D. Fan , L. Xia , Hepatology 2015, 61, 1920. 25704764 10.1002/hep.27756

[17] Y. Fu , B. Mackowiak , D. Feng , H. Lu , Y. Guan , T. Lehner , H. Pan , X. W. Wang , Y. He , B. Gao , Gut 2023, 72, 1942. 36593103 10.1136/gutjnl-2022-327924 PMC11283862

[18] C. Tay , A. Tanaka , S. Sakaguchi , Cancer Cell 2023, 41, 450. 36917950 10.1016/j.ccell.2023.02.014

[19] X. Lin , K. Kang , P. Chen , Z. Zeng , G. Li , W. Xiong , M. Yi , B. Xiang , Mol. Cancer 2024, 23, 108. 38762484 10.1186/s12943-024-02023-w PMC11102195

[20] V. Leone , A. Ali , A. Weber , D. F. Tschaharganeh , M. Heikenwalder , Trends Cancer 2021, 7, 606. 33674229 10.1016/j.trecan.2021.01.012

[21] D. V. F. Tauriello , E. Sancho , E. Batlle , Nat. Rev. Cancer 2022, 22, 25. 34671117 10.1038/s41568-021-00413-6

[22] A. Mazzone , G. Ricevuti , Haematologica 1995, 80, 161. 7628754

[23] F. Veglia , E. Sanseviero , D. I. Gabrilovich , Nat. Rev. Immunol. 2021, 21, 485. 33526920 10.1038/s41577-020-00490-y PMC7849958

[24] A. Etzerodt , S. K. Moestrup , Antioxid. Redox Signal 2013, 18, 2352. 22900885 10.1089/ars.2012.4834 PMC3638564

[25] D. Melisi , D. Y. Oh , A. Hollebecque , E. Calvo , A. Varghese , E. Borazanci , T. Macarulla , V. Merz , C. Zecchetto , Y. Zhao , I. Gueorguieva , M. Man , L. Gandhi , S. T. Estrem , K. A. Benhadji , M. C. Lanasa , E. Avsar , S. C. Guba , R. Garcia‐Carbonero , J. Immunother. Cancer 2021, 9, e002068. 33688022 10.1136/jitc-2020-002068 PMC7944986

[26] R. B. Holmgaard , D. A. Schaer , Y. Li , S. P. Castaneda , M. Y. Murphy , X. Xu , I. Inigo , J. Dobkin , J. R. Manro , P. W. Iversen , D. Surguladze , G. E. Hall , R. D. Novosiadly , K. A. Benhadji , G. D. Plowman , M. Kalos , K. E. Driscoll , J. Immunother. Cancer 2018, 6, 47. 29866156 10.1186/s40425-018-0356-4 PMC5987416

[27] R. Khattri , T. Cox , S. A. Yasayko , F. Ramsdell , Nat. Immunol. 2003, 4, 337. 28115588

[28] S. Tanaka , A. Suto , T. Iwamoto , T. Kageyama , T. Tamachi , H. Takatori , K. Suzuki , K. Hirose , O. Ohara , V. Lefebvre , H. Nakajima , J. Exp. Med. 2018, 215, 2509. 30190287 10.1084/jem.20172082 PMC6170178

[29] K. I. Suehiro , A. Suto , K. Suga , H. Furuya , A. Iwata , T. Iwamoto , S. Tanaka , T. Kageyama , K. Suzuki , K. Hirose , V. Lefebvre , H. Nakajima , Cell Mol. Immunol. 2021, 18, 1729. 32152552 10.1038/s41423-020-0384-0 PMC8245422

[30] P. Yang , Q. J. Li , Y. Feng , Y. Zhang , G. J. Markowitz , S. Ning , Y. Deng , J. Zhao , S. Jiang , Y. Yuan , H. Y. Wang , S. Q. Cheng , D. Xie , X. F. Wang , Cancer Cell 2012, 22, 291. 22975373 10.1016/j.ccr.2012.07.023 PMC3443566

[31] R. Yamagishi , F. Kamachi , M. Nakamura , S. Yamazaki , T. Kamiya , M. Takasugi , Y. Cheng , Y. Nonaka , Y. Yukawa‐Muto , L. T. T. Thuy , Y. Harada , T. Arai , T. M. Loo , S. Yoshimoto , T. Ando , M. Nakajima , H. Taguchi , T. Ishikawa , H. Akiba , S. Miyake , M. Kubo , Y. Iwakura , S. Fukuda , W. Y. Chen , N. Kawada , A. Rudensky , S. Nakae , E. Hara , N. Ohtani , Sci. Immunol. 2022, 7, eabl7209. 35749514 10.1126/sciimmunol.abl7209

[32] O. Yoshie , K. Matsushima , Int. Immunol. 2015, 27, 11. 25087232 10.1093/intimm/dxu079

[33] L. Rimassa , R. S. Finn , B. Sangro , J. Hepatol. 2023, 79, 506. 36933770 10.1016/j.jhep.2023.03.003

[34] H. Madhi , J. S. Lee , Y. E. Choi , Y. Li , M. H. Kim , Y. Choi , S. H. Goh , Adv. Sci. 2022, 9, e2202702. 10.1002/advs.202202702 PMC9561767 35975458

[35] Z. Wang , B. Pan , J. Qiu , X. Zhang , X. Ke , S. Shen , X. Wu , Y. Yao , N. Tang , Sci. Signal 2023, 16, eabq3362. 36917642 10.1126/scisignal.abq3362

[36] M. Marzec , Q. Zhang , A. Goradia , P. N. Raghunath , X. Liu , M. Paessler , H. Y. Wang , M. Wysocka , M. Cheng , B. A. Ruggeri , M. A. Wasik , Proc. Natl. Acad. Sci. U. S. A. 2008, 105, 20852. 19088198 10.1073/pnas.0810958105 PMC2634900

[37] N. R. Gough , X. Xiang , L. Mishra , Gastroenterology 2021, 161, 434. 33940008 10.1053/j.gastro.2021.04.064 PMC8841117

[38] Y. Zheng , Y. Wang , Z. Lu , J. Wan , L. Jiang , D. Song , C. Wei , C. Gao , G. Shi , J. Zhou , J. Fan , A. Ke , L. Zhou , J. Cai , Adv. Sci. 2023, 10, e2301928. 10.1002/advs.202301928 PMC10582428 37705495

[39] J. Shimizu , S. Yamazaki , S. Sakaguchi , J. Immunol. 1999, 163, 5211. 10553041

[40] S. Funaki , Y. Shintani , T. Kawamura , R. Kanzaki , M. Minami , M. Okumura , Oncol. Rep. 2017, 38, 2277. 28849209 10.3892/or.2017.5894

[41] S. Mariathasan , S. J. Turley , D. Nickles , A. Castiglioni , K. Yuen , Y. Wang , E. E. Kadel III , H. Koeppen , J. L. Astarita , R. Cubas , S. Jhunjhunwala , R. Banchereau , Y. Yang , Y. Guan , C. Chalouni , J. Ziai , Y. Senbabaoglu , S. Santoro , D. Sheinson , J. Hung , J. M. Giltnane , A. A. Pierce , K. Mesh , S. Lianoglou , J. Riegler , R. A. D. Carano , P. Eriksson , M. Hoglund , L. Somarriba , D. L. Halligan , et al., Nature 2018, 554, 544. 29443960 10.1038/nature25501 PMC6028240

[42] J. Chen , W. Feng , M. Sun , W. Huang , G. Wang , X. Chen , Y. Yin , X. Chen , B. Zhang , Y. Nie , D. Fan , K. Wu , L. Xia , Gastroenterology 2024, 167, 264. 38417530 10.1053/j.gastro.2024.02.025

[43] Q. He , M. Liu , W. Huang , X. Chen , B. Zhang , T. Zhang , Y. Wang , D. Liu , M. Xie , X. Ji , M. Sun , D. Tian , L. Xia , Hepatology 2021, 74, 3174. 34288020 10.1002/hep.32062

[44] M. Xie , Z. Lin , X. Ji , X. Luo , Z. Zhang , M. Sun , X. Chen , B. Zhang , H. Liang , D. Liu , Y. Feng , Y. Wang , Y. Li , B. Liu , W. Huang , L. Xia , J. Hepatol. 2023, 79, 109. 36907560 10.1016/j.jhep.2023.02.036

