# Supplementary material for: SOX12 Facilitates Hepatocellular Carcinoma Progression and Metastasis through Promoting Regulatory T‐Cells Infiltration and Immunosuppression
Source: Adv Sci (Weinh). 2024 Jul 29;11(36):2310304. doi: 10.1002/advs.202310304 (PMC11423149; doi:10.1002/advs.202310304)
Supplement: Supplementary file 1 — Supporting Information [file ADVS-11-2310304-s001.docx]

**SOX12 Facilitates Hepatocellular Carcinoma Progression and Metastasis through Promoting Regulatory T cells Infiltration and Immunosuppression**

*Xiangyuan Luo^#^, Wenjie Huang^#^, Siwen Li^#^, Mengyu Sun, Dian Hu, Junqing Jiang, Zerui Zhang, Yijun Wang, Yufei Wang, Jiaqian Zhang, Zhangfan Wu, Xiaoyu Ji, Danfei Liu, Xiaoping Chen, Bixiang Zhang, Huifang Liang, Yiwei Li, Bifeng Liu, Shuai Wang, Xiao Xu, Yongzhan Nie, Kaichun Wu, Daiming Fan, Limin Xia^*^*

**Supplementary materials and methods**

**Cell lines and culture**

HCC cells (Huh7) and mouse HCC cells (Hepa1-6) were purchased from the Institute of Biochemistry and Cell Biology, Chinese Academy of Science, China. Human HCC cells (Hep3B) were purchased from the American Type Culture Collection. H22 cells were kindly provided by the State key Laboratory of Cancer Biology, National Clinical Research Center for Digestive Diseases and Xijing Hospital of Digestive Diseases, Fourth Military Medical University. Additional human HCC cells (MHCC97H and HCCLM3) were kindly provided by Dr. Tang ZY (Liver Cancer Institute, Zhongshan Hospital, Fudan University, Shanghai, China). Cells were cultured in Dulbecco’s Modified Eagle Medium (DMEM) or RPMI-1640 medium at 37°C in a 5% CO_2_ incubator. The medium was supplemented with 10% FBS, 100μg/ml penicillin, and 100μg/ml streptomycin. These above cell lines were authenticated by short tandem repeats (STRs) DNA profiling. All cells were tested for mycoplasma contamination before use with the Universal Mycoplasma Detection Kit (ATCC 30-1012K) and were not contaminated by mycoplasma.

**Plasmid construction**

Plasmid construction was performed according to standard procedures. The primers were shown in **Table S11**. For example, the *SOX12* gene complete CDS construct, pCMV-SOX12, was generated by using cDNA from human PBMCs. It was generated with forward and reverse primers incorporating *EcoRI* and *HindIII* sites at the 5’ and 3’-ends, respectively. The polymerase chain reaction (PCR) product was cloned into the *EcoRI* and *HindIII* sites of the pCMV-Tag2B vector. The *CCL22* promoter construct (-1127/+148) was generated from human genomic DNA. This construct corresponds to sequence from -1127 to +148 (relative to the transcriptional start site) of the 5’-flanking regions of human *CCL22* gene. It was generated with forward and reverse primers incorporating *KpnI* and *HindIII* sites at the 5’ and 3’-ends, respectively. The PCR product was cloned into the *KpnI* and *HindIII* sites of the pGL3-Basic vector (Promega). The 5’-flanking deletion constructs of the *CCL22* promoter, (-454/+148) CCL22, (-315/+148) CCL22 were similarly generated using the (-1127/+148) CCL22 construct as the template. The SOX12 binding sites in the *CCL22* promoter were mutated using the QuikChange II Site-Directed Mutagenesis Kit (Stratagene). The constructs were confirmed by DNA sequencing. Other promoter constructs were cloned in the same manner.

**Construction of stable cell lines**

Lentiviral vectors encoding shRNAs were generated using PLKO.1-TRC (Addgene) and designated as LV-shSOX12, LV-shCCL22, LV-shPD-L1, LV-shSmad2, LV-shSmad3, LV-shSmad4, and LV-shControl. “LV-shControl” is a non-target shRNA control. The vector “pLKO.1-puro Non-Target shRNA Control Plasmid DNA” (Sigma, SHC016) contains an shRNA insert that does not target any known genes from any species. The shRNA sequences can be found in **Table S12**. Lentiviral vectors encoding human or mouse *SOX12*, *CCL22*, and *TGFB1* genes were constructed in Plenti-CMV-Puro (Addgene plasmid #17448) and designated as LV-SOX12, LV-CCL22, and LV-TGF-β1. An empty vector was used as the negative control and was designated as LV-Control. The lentivirus and cell infection were produced according to the lentiviral vector protocol recommended by Addgene. Briefly, the lentiviral plasmid and packaging plasmids pMD2.G and psPAX2 (Addgene plasmid #12259 and #12260) were transfected into HEK-293T cells with transfection reagent PEI (Polyethylenimine, Thermo Fisher Scientific) and DMEM media for 72 hours. Viruses were filtered with a 0.45-μm filter and stored at -80 °C. Lentiviral infection of target cells was performed in cell culture media with 10 μg/ml polybrene (Sigma H9268) for 24 hours. After 48 hours of culture, cells were selected for one week using 2.5 μg/ml puromycin (OriGene). HCC cells expressing luciferase were constructed by infecting the specific cells with viruses expressing luciferase in the same way as described above, and then the cells were selected with 10 μg/ml blasticidin (Merck). Selected pools of cells were used for the following experiments.

For establishing SOX12 knockout cell lines, we utilized a lentivirus-based CRISPR/Cas9 system ^[1]^. Briefly, the sgRNA targeting SOX12 (5’-GCTGCAGGAAGAGCCCGCGG-3’) was designed by an online CRISPR Guide RNA Design Tool (https://www.benchling.com/crispr). The sgRNA was annealed and ligated into the LentiCRISPRv2 vector (Addgene plasmid #52961) and co-transfected with packaging plasmids pMD2.G and pxPAX2 into HEK-293T cells for 72 hours. The viral supernatant was collected and infected with targeted HCC cells. After one week of puromycin selection, the remaining cells were seeded into 96-well plates, and monoclonal cells were picked by limiting dilution. Western blot was used to confirm the knockout efficiency of the selected monoclonal cells.

**Transient transfection**

The cells were plated at a density of 1×10^5^ cells/well in a 24-well plate. After 12-24 hours, the cells were co-transfected with 0.6μg of expression vector plasmids, 0.18μg of promoter reporter plasmids, and 0.02μg of pRL-TK plasmids using Lipofectamine 2000 (Invitrogen, USA) according to the manufacturer’s instructions. After 6h of transfection, the cells were washed and allowed to recover overnight in fresh medium supplemented with 1% FBS for 48 h. Serum-starved cells were used for the assay.

**Luciferase reporter assay**

Luciferase activity was detected using the Dual Luciferase Assay (Promega, USA) according to the manufacturer’s instructions. The transfected cells were lysed in culture dishes containing a lysis buffer, and the resulting lysates were centrifuged at maximum speed for 1 min in a microcentrifuge. Relative luciferase activity was determined using a ModulusTM TD20/20 Luminometer (Turner Biosystems, USA), and the transfection efficiencies were normalized according to the Renilla activity.

**Western blot analysis**

Proteins from lysed cells were fractionated by SDS-PAGE and transferred to nitrocellulose membranes. Nonspecific binding sites were blocked with 5% bovine serum albumin (BSA) in TBST (120 mM Tris–HCl (pH 7.4), 150 mM NaCl, and 0.05% Tween 20) for 2 hours at room temperature. Blots were incubated with a specific antibody overnight at 4 °C. Western blotting of β-actin on the same membrane was used as a loading control. The membranes were then washed with TBST three times and incubated with an HRP-conjugated secondary antibody. Proteins were visualized using an ImmobilonTM Western Chemiluminescent HRP substrate (Millipore, USA).

The primary antibodies used are listed below.

| Antibodies | Source |
| --- | --- |
| anti-SOX12 | Proteintech, 23939-1-AP (1:1000) |
| anti-CCL22 | Affinity, DF7781 (1:1000) |
| anti-PD-L1 | Cell signaling technology, #13684 (1:1000) |
| anti-Smad2 | Cell signaling technology, #5339 (1:1000) |
| anti-Smad3 | Cell signaling technology, #9523 (1:1000) |
| anti-p-Smad3 (Ser423/425) | Cell signaling technology, #9520 (1:1000) |
| anti-Smad4 | Cell signaling technology, #46535 (1:1000) |
| anti-p-Smad2(Ser465/467)/p-Smad3 (Ser423/425) | Cell signaling technology, #8828 (1:1000) |
| anti-AKT | Cell signaling technology, #4685 (1:1000) |
| anti-p-AKT (Ser473) | Cell signaling technology, #4060 (1:1000) |
| anti-JNK | Abcam, ab179461 (1:1000) |
| anti-p-JNK (phosphorT183+T183+T221) | Abcam, ab124956 (1:1000) |
| anti-ERK1/2 | Cell Signaling Technology, #9102 (1:1000) |
| anti-p-ERK1/2 (T202/Y204) | Cell Signaling Technology, #4370 (1:1000) |
| anti-p38 | Cell Signaling Technology, #8690 (1:1000) |
| anti-p-p38 (Thr180/Tyr182) | Cell Signaling Technology, #4511 (1:1000) |
| Anti-Cofilin | Cell Signaling Technology, #5175 (1:1000) |
| Anti-p-Cofilin (Ser3) | Cell Signaling Technology, #3313 (1:1000) |
| anti-p65 | Cell signaling technology, #8242 (1:1000) |
| anti-p-p65 (Ser536) | Cell signaling technology, #3033 (1:1000) |
| anti-TGF-β1 | Cell signaling technology, #3711S (1:1000) |
| anti-β-actin | Proteintech, 66009-1-Ig (1:20000) |

**Quantitative Real-time PCR (RT-qPCR)**

The RNeasy Plus Mini Kit (50) kit (Qiagen, Hilden, Germany) was used to extract total RNA, which was then reverse transcribed with the Advantage RT-for-PCR Kit (Qiagen) in accordance with the manufacturer’s protocols. The target sequence was amplified with real-time PCR with the SYBR Green PCR Kit (Qiagen). The cycling parameters used were 95 °C for 15 s, 55-60 °C for 15 s, and 72 °C for 15 s for 45 cycles. Melting curve analyses were performed, and Ct values were determined during the exponential amplification phase of real-time PCR. SDS 1.9.1 software (Applied Biosystems, Massachusetts, USA) was used to evaluate amplification plots. The 2^–ΔΔCt^ method was used to determine relative fold changes in target gene expression in cell lines, which was normalized to expression levels in corresponding control cells (defined as 1.0). The equation used was 2^–ΔΔCt^ (ΔCt = Ct^target^ – Ct^ATCB^; ΔΔCt = ΔCt^expressing vector^ – ΔCt^control vector^). All experiments were performed in duplicate. The primer sequences were listed in **Table S11**.

**ELISA**

HCC cells were seeded in 96-well plates and cultured for 72 hours. Then we collected the supernates and used Human CCL22/MDC DuoSet ELISA (RD, #DY336) to detect CCL22 protein and used Mouse TGF-beta 1 DuoSet ELISA (RD, #DY1679) to detect TGF-β1 protein according to the manufacturer’s instructions. The culture medium was used as a control for normalization. Determine the optical density of each well immediately, using a microplate reader set to 450 nm.

**Cell treatment**

Established HCC cell lines were seeded in 6-well plates and allowed to attach overnight. Reagents used to treat these cells were listed below.

| Reagents | Source |
| --- | --- |
| Recombinant human IL-1β | PeproTech (10ng/mL) |
| Recombinant human IL-6 | PeproTech (50ng/mL) |
| Recombinant human IL-8 | PeproTech (50ng/mL) |
| Recombinant human IL-17A | PeproTech (50ng/mL) |
| Recombinant human IL-27 | PeproTech (25ng/mL) |
| Recombinant human TGF-β1 | PeproTech (5ng/mL) |
| Recombinant human TNF-α | PeproTech (10ng/mL) |
| Recombinant human IFN-γ | PeproTech (10ng/mL) |
| Recombinant human HMGB1 | Abcam (1 μg/mL) |
| ERK inhibitor U0126 | Selleck (10μM) |
| JNK inhibitor SP600125 | MedChemExpress (20μM) |
| p38 inhibitor SB203580 | MedChemExpress (20μM) |
| PI3K inhibitor LY294002 | MedChemExpress (10μM) |
| Smad3 inhibitor SIS3 | MedChemExpress (2μM) |
| ROCK inhibitor Y27632 | MedChemExpress (2.5μM) |
| NF-kB inhibitor BAY 11-7082 | MedChemExpress (10μM) |

**Construction of tissue microarrays and immunohistochemistry**

HCC samples and the corresponding adjacent liver tissues were used to construct a tissue microarray (Shanghai Biochip Co., Ltd. Shanghai, China). IHC was performed on 4-μm-thick, routinely processed paraffin-embedded sections. Briefly, the tissue sections were deparaffinized after baking at 60 °C for an hour. Endogenous peroxidase activity was blocked by 3% (vol/vol) hydrogen peroxide in methanol for 12 min and washes with phosphate-buffered saline (PBS). Then the slides were immersed in 0.01 mol/L citrate buffer solution (pH 6.0) and placed in a microwave oven for 30 min. After washed with PBS, the sections were incubated with BSA for 30 min, then incubated with the primary antibody diluted in PBS containing 1% (wt/vol) BSA in 4 °C for overnight. The tissue microarrays were stained for SOX12 (assaybiotech, C10082, 1:100), CCL22 (ABclonal, A1966, 1:100), PD-L1 (cell signaling technology, #13684, 1:100), Foxp3 (cell signaling technology, D2W8E, 1:100), CD163 (Abcam, ab87099, 1:400), CD11b (Abcam, ab133357, 1:4000), and CD8 (cell signaling technology, C8/144B, 1:200). Negative controls were performed by replacing the primary antibody with preimmune mouse serum. After washing with PBS, the sections were treated with a peroxidase-conjugated second antibody (Santa Cruz) for 30 min at room temperature and then washed with PBS. Reaction product was visualized with diaminobenzidine for 2 min. Images were obtained under a light microscope (Olympus, Japan) equipped with a DP70 digital camera.

Analyses were performed by two independent observers who were blinded to the clinical outcome. The immunostaining intensity was scored on a scale of 0 to 3: 0 (negative), 1 (weak), 2 (medium), or 3 (strong). The percentage of positive cells was evaluated on a scale of 0 to 4: 0 (negative), 1 (1%-25%), 2 (26%-50%), 3 (51%-75%), or 4 (76%-100%). The final immuno-activity scores were calculated by multiplying the above two scores, resulting in an overall score that ranges from 0~12. Each case was ultimately considered “negative” if the final score ranged from 0~3 and “positive” if the final score ranged from 4~12 as described previously ^[2]^.

**Preparation of Single Cell Suspensions**

Prior to flow cytometry analysis, single cell suspensions should be prepared. The method was used as described in the research paper ^[3]^. Briefly, after the anesthetization of mice, Hank’s buffer without calcium was first injected into the liver through the portal vein and then the Hank’s buffer including calcium, magnesium and collagenase IV (0.2 mg/mL, Sigma-Aldrich, C5138) followed. After separation of the liver and tumor, the tissues were made into small pieces about 1mm^3^. Mouse tumor dissociation buffer (Miltenyi, 130-096-730) was used to prepare the single cell by using the gentleMACS dissociator (Miltenyi) followed by filtrating through a 70μm cell mesh, lysing erythrocyte, centrifuging, and resuspending in Hank’s buffer.

**Flow cytometry**

After the anesthetization of mice, tumors were collected to prepare the single cell suspensions according to the procedure described above. Fc block was added to the cells at room temperature for 10 minutes and then incubated with primary antibodies or isotype antibodies at 4°C for 45 minutes. A FACS LSRFortessa and FlowJo software (BD Biosciences) were used to acquire and analyze the data, respectively.

Antibodies used in Flow Cytometric Analysis

| Antibodies | Source |
| --- | --- |
| anti-CD45 | BD, 559864 |
| anti-F4/80 | eBioscience, 45-4801-82 |
| anti-CD206 | eBioscience, 12-2061-82 |
| anti-CD3 | BD, 555275 |
| anti-CD8a | BD, 564459 |
| anti-PD1 | BD, 563059 |
| anti-Tim3 | BD, 747626 |
| anti-CD11b | BD, 564454 |
| anti-Gr-1 | BD, 553128 |
| FVS-510 | BD, 564406 |
| anti-CD4 | BD, 566407 |
| anti-CD25 | BD, 564424 |
| anti-Foxp3 | BD, 560401 |
| anti-CD11c | BD, 563057 |
| anti-NK1.1 | BD, 740853 |

**Immunofluorescence**

For slide preparation, the fixed tumor tissues of the mice were embedded, sectioned, dewaxed, and hydrated. The slides were blocked with 10% goat or donkey serum after epitope retrieval. Subsequently, the slides were incubated with different primary antibodies overnight. The following day, different fluorophore-labeled secondary antibodies were incubated. Finally, the slides were stained with DAPI and photographed under an Olympus microscope.

Antibodies used in Immunofluorescence

| Antibodies | Source |
| --- | --- |
| anti-Foxp3 | Cell signaling technology, #12653 (1:100) |
| anti-F4/80 | Cell signaling technology, #70076 (1:200) |
| anti-CD11b | Abcam, ab133357 (1:250) |
| anti-CD8a | Invitrogen, 4SM15 (1:100) |

| anti-Granzyme B | RD, AF1865 (1:100) |
| --- | --- |
| anti-PanCK | Cell signaling technology, #4545 (1:100) |
| anti-CD68 | ZSGB-BIO, ZM-0060 (1:570) |
| anti-CCL22 | ABclonal, A1966 (1:100) |

***In vitro* migration assay of Tregs**

Healthy volunteers-derived PBMCs were resuspended in PBS and incubated with CD4, CD8, and CD25 monoclonal antibodies for 15-30 mins. Then we used flow cytometry to sort the cells with CD4 positive, CD8 negative, and CD25 positive cells (CD4^+^CD8^-^CD25^+^) as Tregs ^[4]^. Tregs were resuspended in serum-free medium and added to the upper chamber of 5-μm pore Transwell inserts (Corning) for 2h to attach to the membrane. The Transwell was then moved to 24-well plates containing 600μl cell conditioned medium with or without C-021 (1μM, MCE) and incubated at 37°C for 24 h. Migrated Cells in the lower chambers were collected and counted by flow cytometry. Three independent experiments were performed for each assay.

**T-cell Suppression Assay**

CD4^+^CD25^+^ Tregs were isolated from liver orthotopic tumors by EasySep™ Mouse CD4^+^CD25^+^ Regulatory-T Cell Isolation Kit II (Stemcell). CD8^+^ T cells were isolated from normal mice spleen by CD8a (Ly-2) MicroBeads (Miltenyi) and Mini & Midi MACS Starting Kit (Miltenyi). Isolated CD8^+^ T cells were stained by CFSE (5uM, BD) for 10 mins at 37°C. Then isolated Tregs were co-cultured with CFSE-labeled CD8^+^ T cells at different ratios (0:1, 1:2, and 1:5) in 1640 with 10% FBS supplemented with anti-CD3 (2 μg/mL, BD), anti-CD28 (2 μg/mL, BD), and IL-2 (20 ng/mL, BD) in a 96-well plate for 72 hours. The purity of cells and proliferation of CD8^+^ T cells were analyzed by flow cytometry.

**RNA sequencing and bioinformatic analysis**

Total RNA was extracted from the negative control and SOX12 overexpression groups of Huh7 cells using TRIzol reagent (Invitrogen), and each group was prepared with three parallel replicates. The quality control, library construction, and RNA sequencing were all performed by Novogene Co., Ltd. For data analysis, differential expression analysis of the two groups was performed using the DESeq2 R package (1.20.0). The cutoff value of differentially expressed genes (DEGs) was set as |log2[fold change (FC)]| > 1 and p < 0.05. The volcano plot was performed using the ggplot2 R package. The enrichment analysis was used the R package clusterProfiler (version 3.14.3).

**Bioinformatics analysis**

The Timer2 web server was used to infer the correlation between immune cells infiltration and SOX12 expression levels in various tumors.

**Supplementary Reference**

[1] F. A. Ran, P. D. Hsu, J. Wright, V. Agarwala, D. A. Scott, F. Zhang. Genome engineering using the CRISPR-Cas9 system. *Nat Protoc* **2013**, *8* (11), 2281, https://doi.org/10.1038/nprot.2013.143.

[2] Q. He, M. Liu, W. Huang, X. Chen, B. Zhang, T. Zhang, Y. Wang, D. Liu, M. Xie, X. Ji, M. Sun, D. Tian, L. Xia. IL-1beta-Induced Elevation of Solute Carrier Family 7 Member 11 Promotes Hepatocellular Carcinoma Metastasis Through Up-regulating Programmed Death Ligand 1 and Colony-Stimulating Factor 1. *Hepatology* **2021**, *74* (6), 3174, https://doi.org/10.1002/hep.32062.

[3] Y. M. Li, Z. Y. Liu, J. C. Wang, J. M. Yu, Z. C. Li, H. J. Yang, J. Tang, Z. N. Chen. Receptor-Interacting Protein Kinase 3 Deficiency Recruits Myeloid-Derived Suppressor Cells to Hepatocellular Carcinoma Through the Chemokine (C-X-C Motif) Ligand 1-Chemokine (C-X-C Motif) Receptor 2 Axis. *Hepatology* **2019**, *70* (5), 1564, https://doi.org/10.1002/hep.30676.

[4] S. Huo, Y. Luo, R. Deng, X. Liu, J. Wang, L. Wang, B. Zhang, F. Wang, J. Lu, X. Li. EBV-EBNA1 constructs an immunosuppressive microenvironment for nasopharyngeal carcinoma by promoting the chemoattraction of Treg cells. *J Immunother Cancer* **2020**, *8* (2), https://doi.org/10.1136/jitc-2020-001588.

**Figure S1**

**
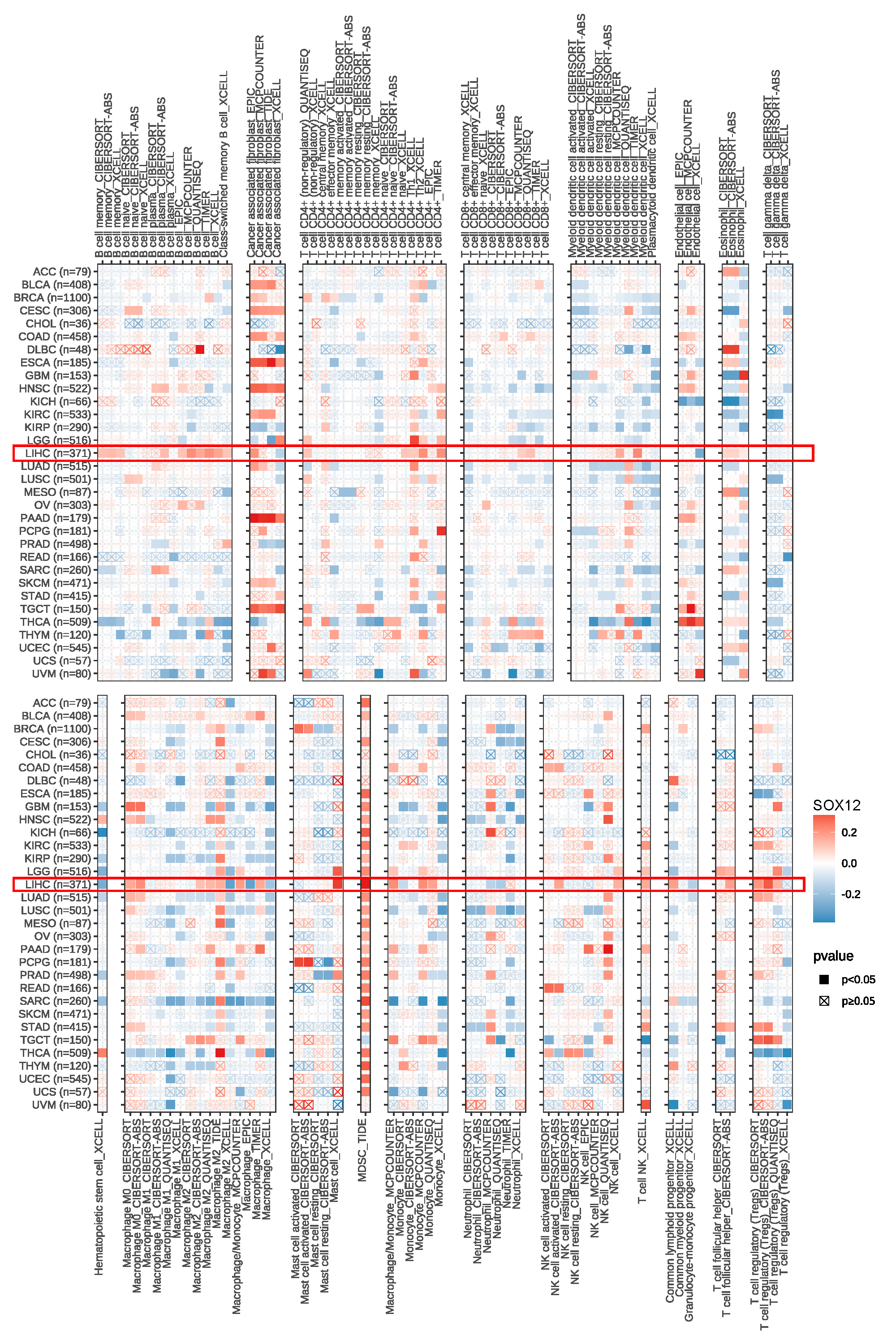
**

**Figure S1.** The relevance of SOX12 expression and immune infiltrates in pan-cancer.

**
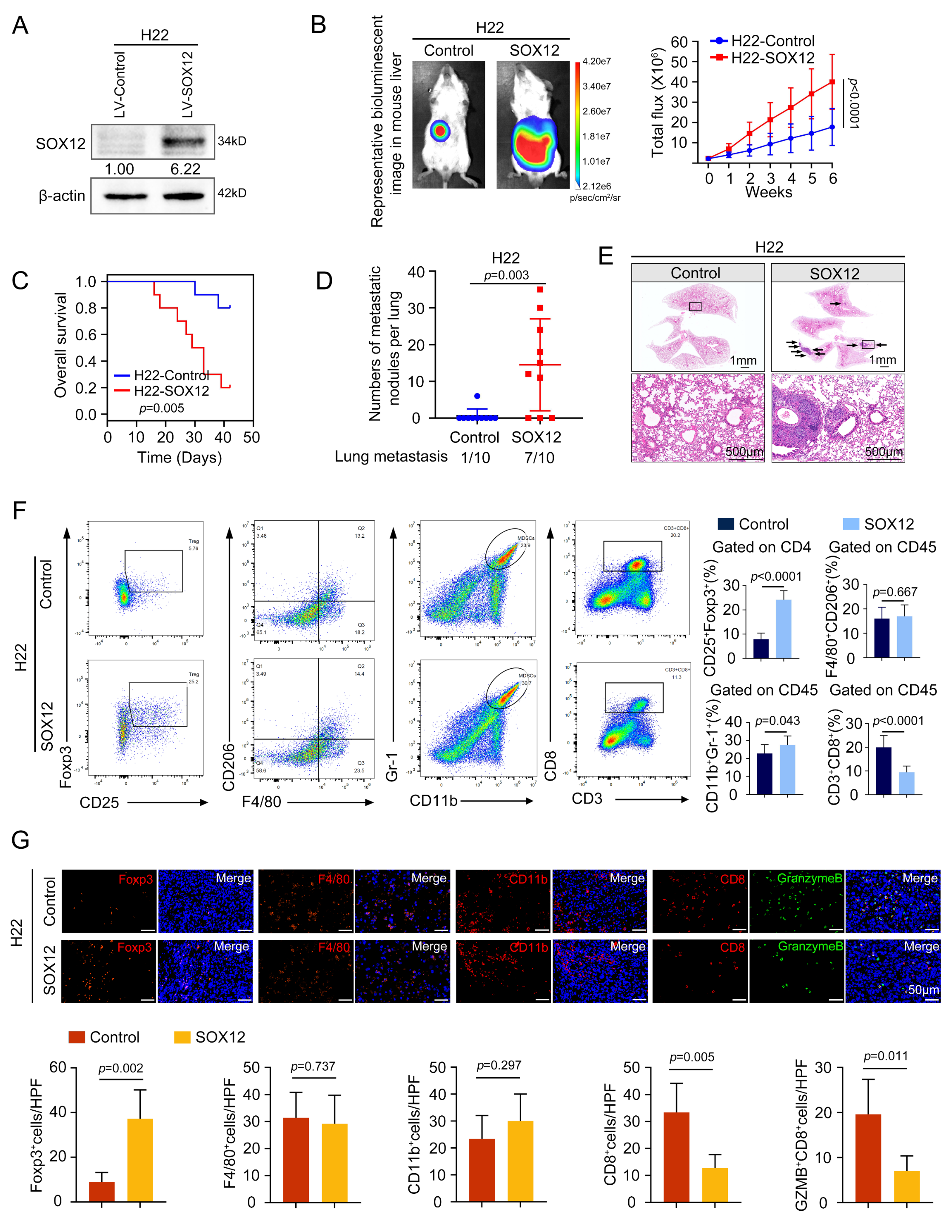
Figure S2**

**Figure S2.** (A) The efficiency of SOX12 overexpression was validated by western blot. (B-G) Construction of intrahepatic orthotopic models bearing H22-SOX12 cells (n=10/group). (B) The representative bioluminescent images and bioluminescence intensity of tumor, (C) overall survival, (D) lung metastatic nodule numbers, and (E) representative lung H&E staining were shown. (F-G) The intratumoral infiltration of CD25^+^Foxp3^+^Tregs, F4/80^+^CD206^+^TAMs, CD11b^+^Gr1^+^MDSCs, and CD3^+^CD8^+^T cell was analyzed by flow cytometry (F) and immunofluorescent staining (G). For (B), Two-way ANOVA. For (C), Long-rank test. For (D), (F), and (G), Unpaired t-test.

**
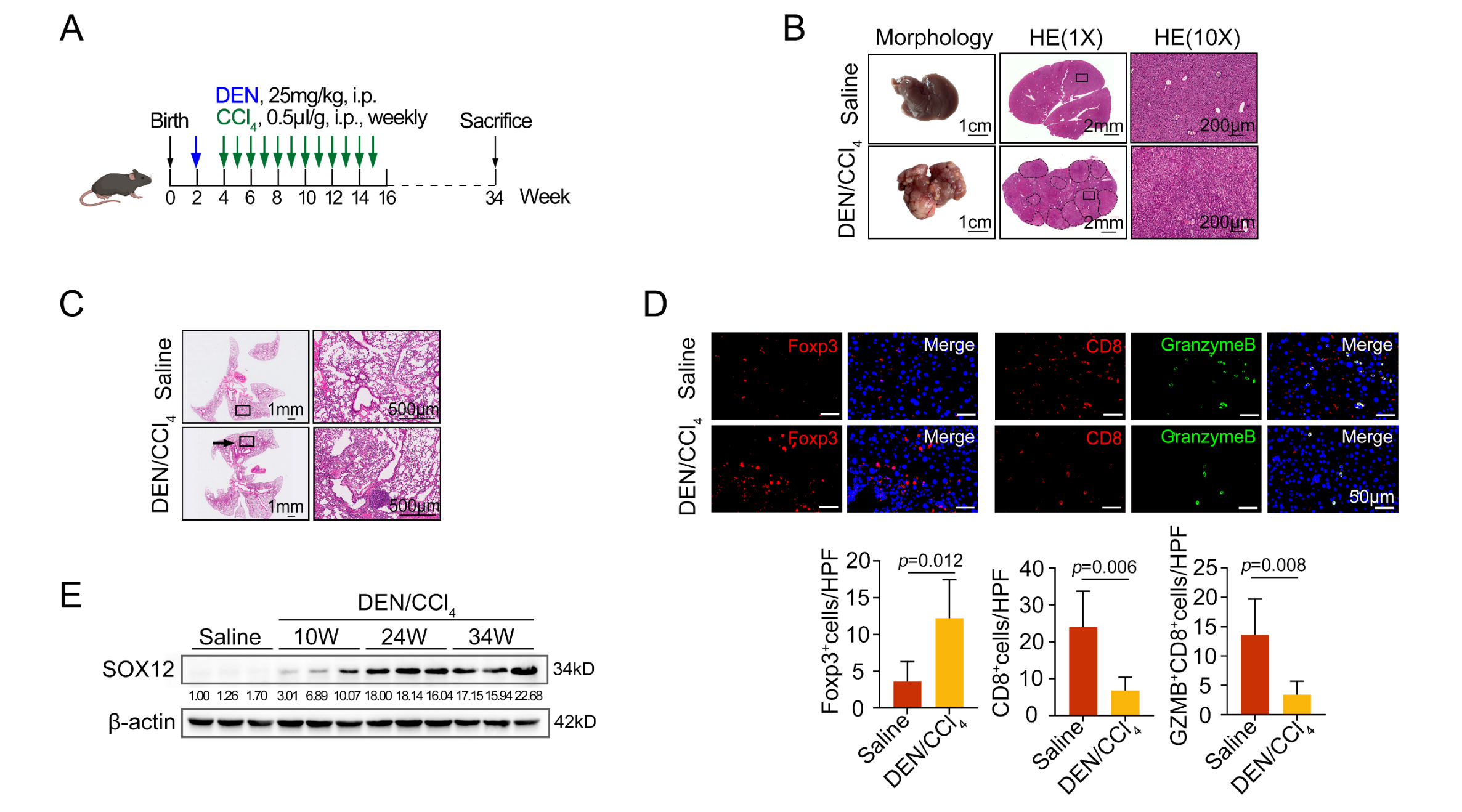
Figure S3**

**Figure S3.** (A) Schematic diagram of the DEN/CCl_4_-induced HCC model. (B) Typical appearances and H&E staining images of the liver from the DEN/CCl_4_ mice or control mice at 34 weeks. (C) Representative lung H&E staining images of the indicated mice. (D) Representative immunofluorescent staining images and histograms of intrahepatic Foxp3^+^cells and CD8^+^GranzymeB^+^ cells in the indicated mice. The significance was determined by Unpaired t-test. (E) The expression of SOX12 in the liver tumors of the DEN/CCl_4_ mice at the indicated times.

**
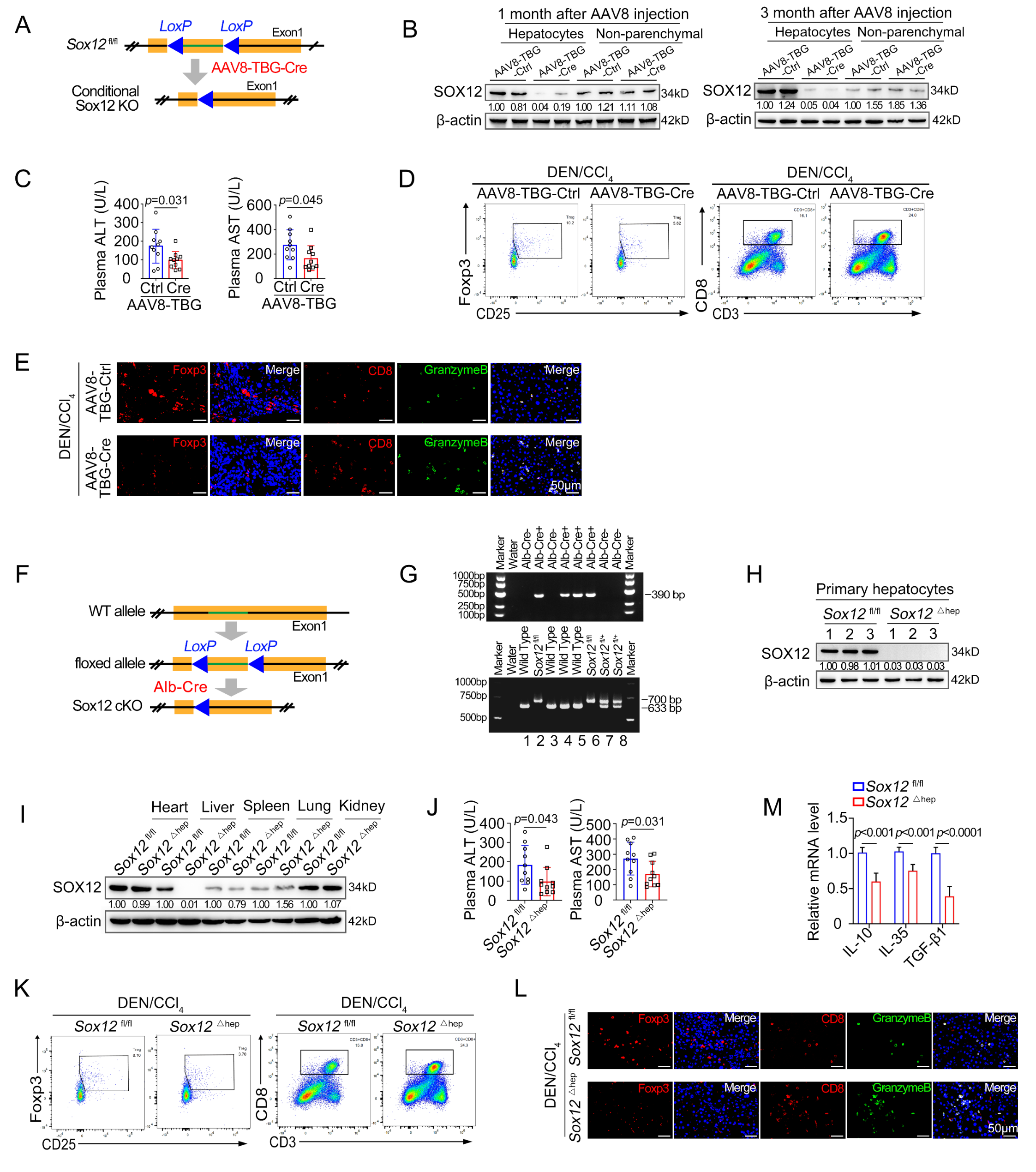
Figure S4**

**Figure S4.** (A) Schematic workflow of establishing the hepatocyte-specific SOX12 knockout mice using the AAV8-TBG-Cre system. (B) The protein levels of SOX12 in hepatocyte and non-parenchymal cells from the indicated mice after 1 or 3 months of AAV8-TBG-Cre injection. (C) The levels of plasma ALT and AST in the indicated mice were detected. The significance was determined by Unpaired t-test. (D-E) Representative flow cytometry images (D) and immunofluorescent staining images (E) of intrahepatic Tregs and CD8^+^ T cells in the indicated mice. (F) Schematic workflow of establishing the hepatocyte-specific SOX12 knockout (*Sox12*^△hep^) mice. (G) Genomic PCR analysis for identifying *Sox12*^△hep^ mice (n=8). (H) The protein level of SOX12 in hepatocytes isolated from the indicated mice. (I) The protein level of SOX12 in five organs of the indicated mice. (J) The levels of plasma ALT and AST in the indicated mice were detected. The significance was determined by Unpaired t-test. (K-L) Representative flow cytometry images (K) and immunofluorescent staining images (L) of intrahepatic Tregs and CD8^+^ T cells in the indicated mice. (M) The mRNA levels of IL-10, IL-35, and TGF-β1 in Tregs from the indicated mice. The significance was determined by Unpaired t-test. ALT, alanine transaminase; AST, aspartate transaminase.


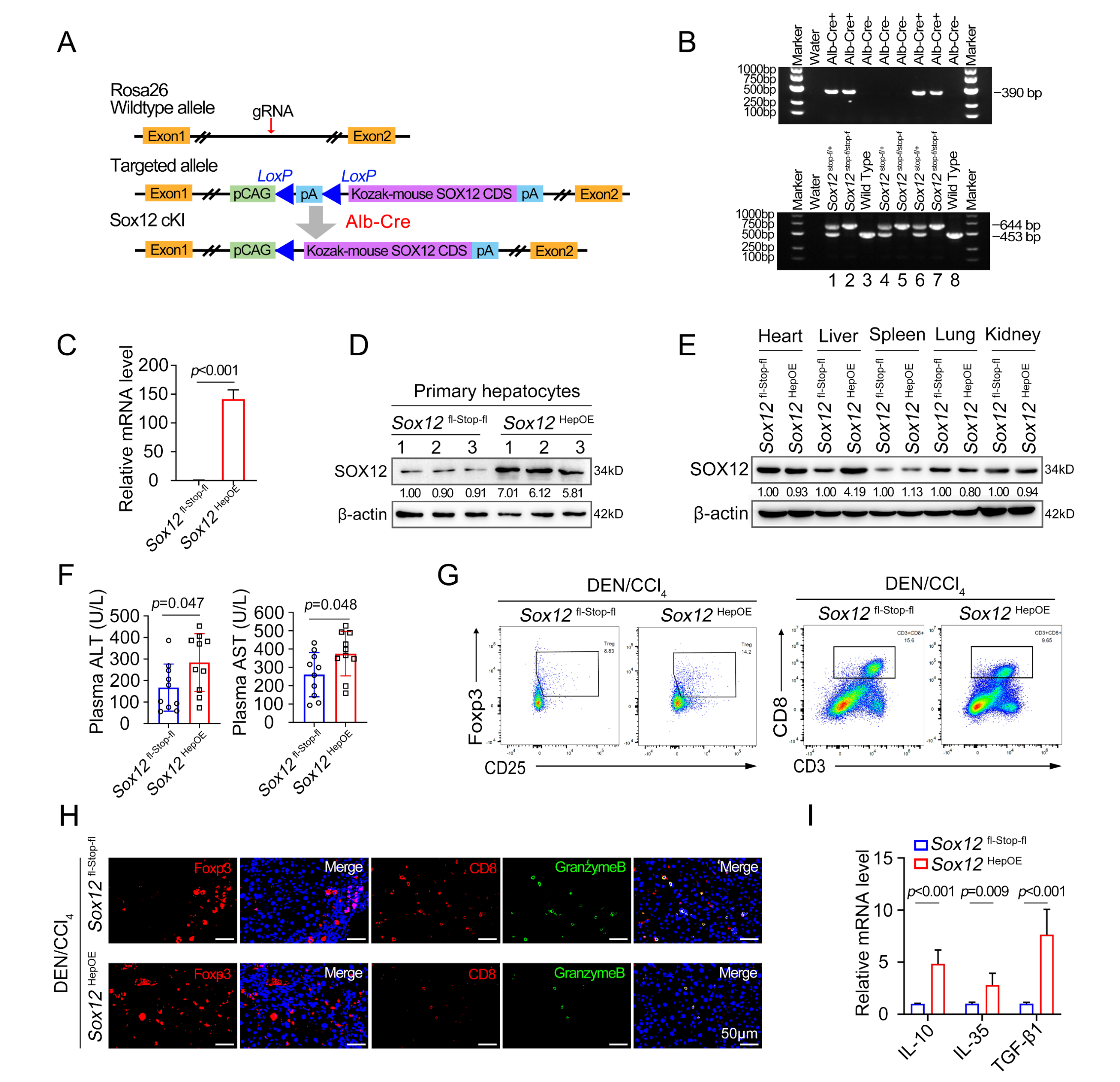
**Figure S5**

**Figure S5.** (A) Schematic workflow of establishing the hepatocyte-specific SOX12 knock-in (*Sox12*^HepOE^) mice. (B) Genomic PCR analysis for identifying *Sox12*^HepOE^ mice (n=8). (C-D) The mRNA (C) and protein level (D) of SOX12 in the hepatocyte of the indicated mice were detected. The significance was determined by Unpaired t-test. (E) The protein level of SOX12 in five organs of the indicated mice. (F) The levels of plasma ALT and AST in the indicated mice were detected. The significance was determined by Unpaired t-test. (G-H) Representative flow cytometry images (G) and immunofluorescent staining images (H) of intrahepatic Tregs and CD8^+^ T cells in the indicated mice. (I) The mRNA levels of IL-10, IL-35, and TGF-β1 in Tregs from the indicated mice. The significance was determined by Unpaired t-test. ALT, alanine transaminase; AST, aspartate transaminase.

**Figure S6**

**
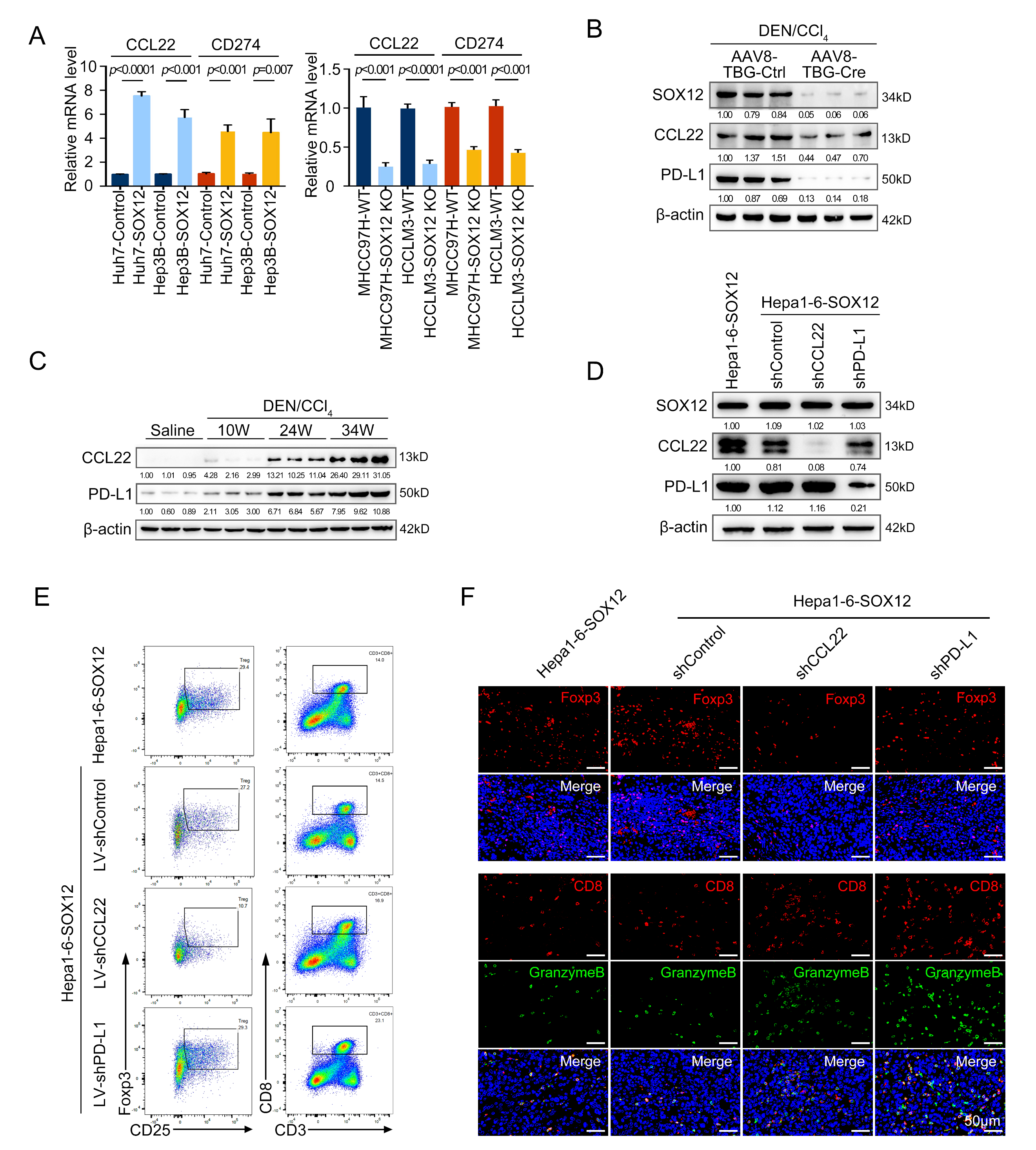
**

**Figure S6.** (A) The expression of CCL22 and PD-L1 in HCC cells with SOX12 overexpression or knockout. The significance was determined by Unpaired t-test. (B) The expression of SOX12, CCL22, and PD-L1 in the AAV8-TBG-Cre mediated hepatocyte-specific SOX12 knockout mice. (C) The expression of CCL22 and PD-L1 in the liver tumors of the DEN/CCl_4_ mice at the indicated times. (D) The knockdown efficiency of CCL22 and PD-L1 in Hepa1-6-SOX12 cells was validated. (E-F) Representative flow cytometry images (E) and immunofluorescent staining images (F) of intrahepatic Tregs and CD8^+^ T cells in the indicated mice.

**Figure S7**


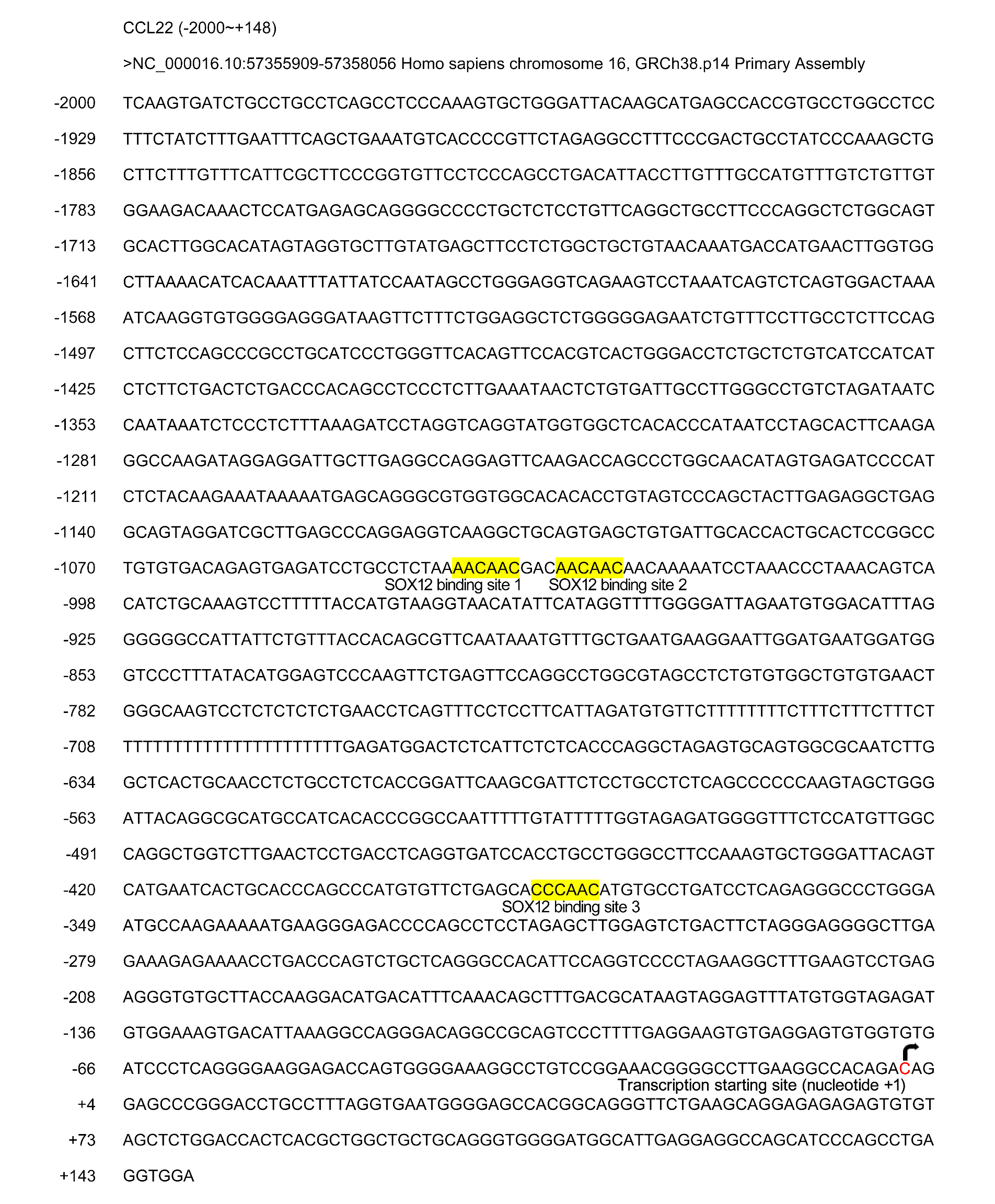


**Figure S7**. The sequence of human *CCL22* promoter. The SOX12 binding sites were highlighted in yellow, and the transcription start site was highlighted in red.


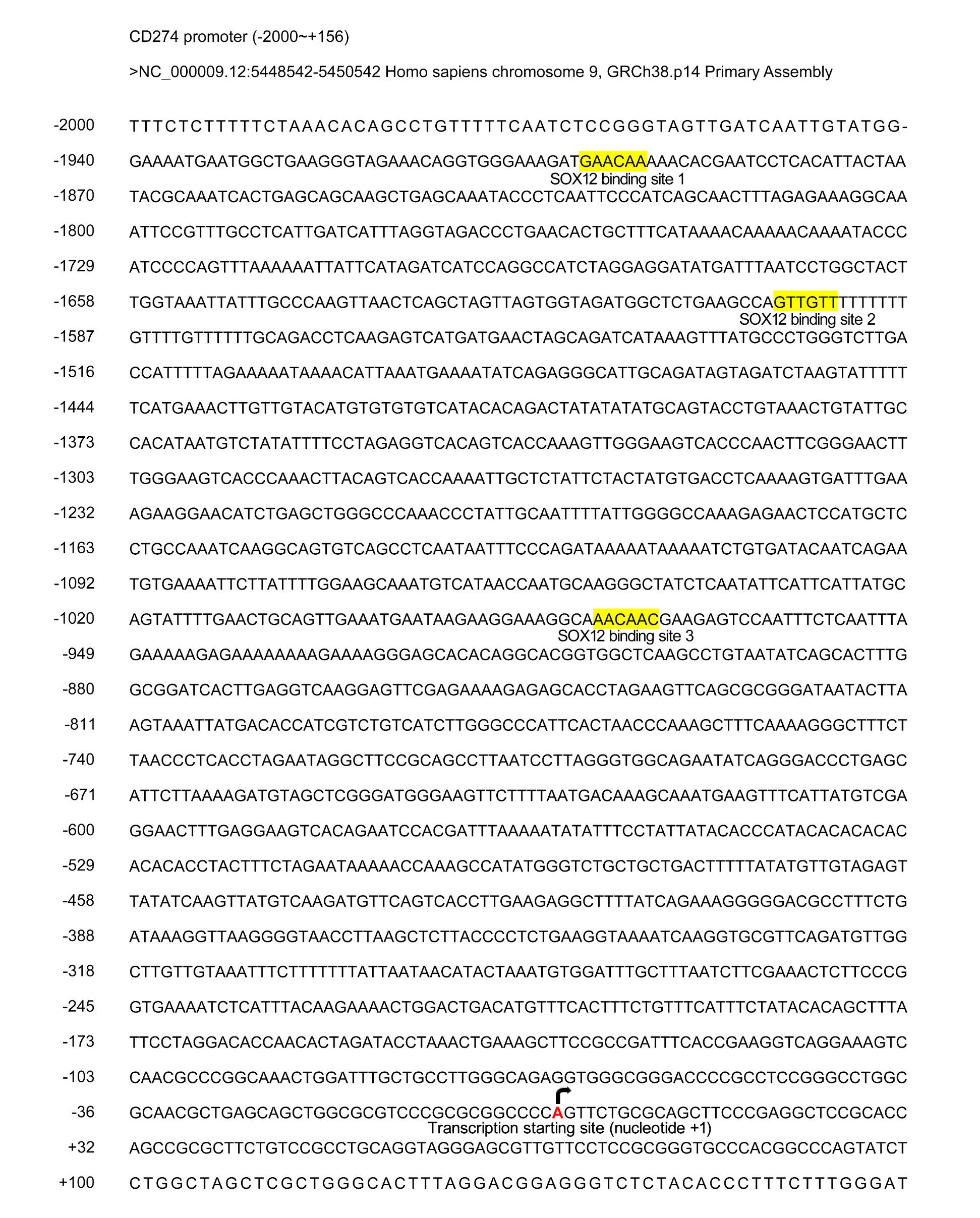
**Figure S8**

**Figure S8**. The sequence of human *CD274* promoter. The SOX12 binding sites were highlighted in yellow, and the transcription start site was highlighted in red.

**Figure S9**

**
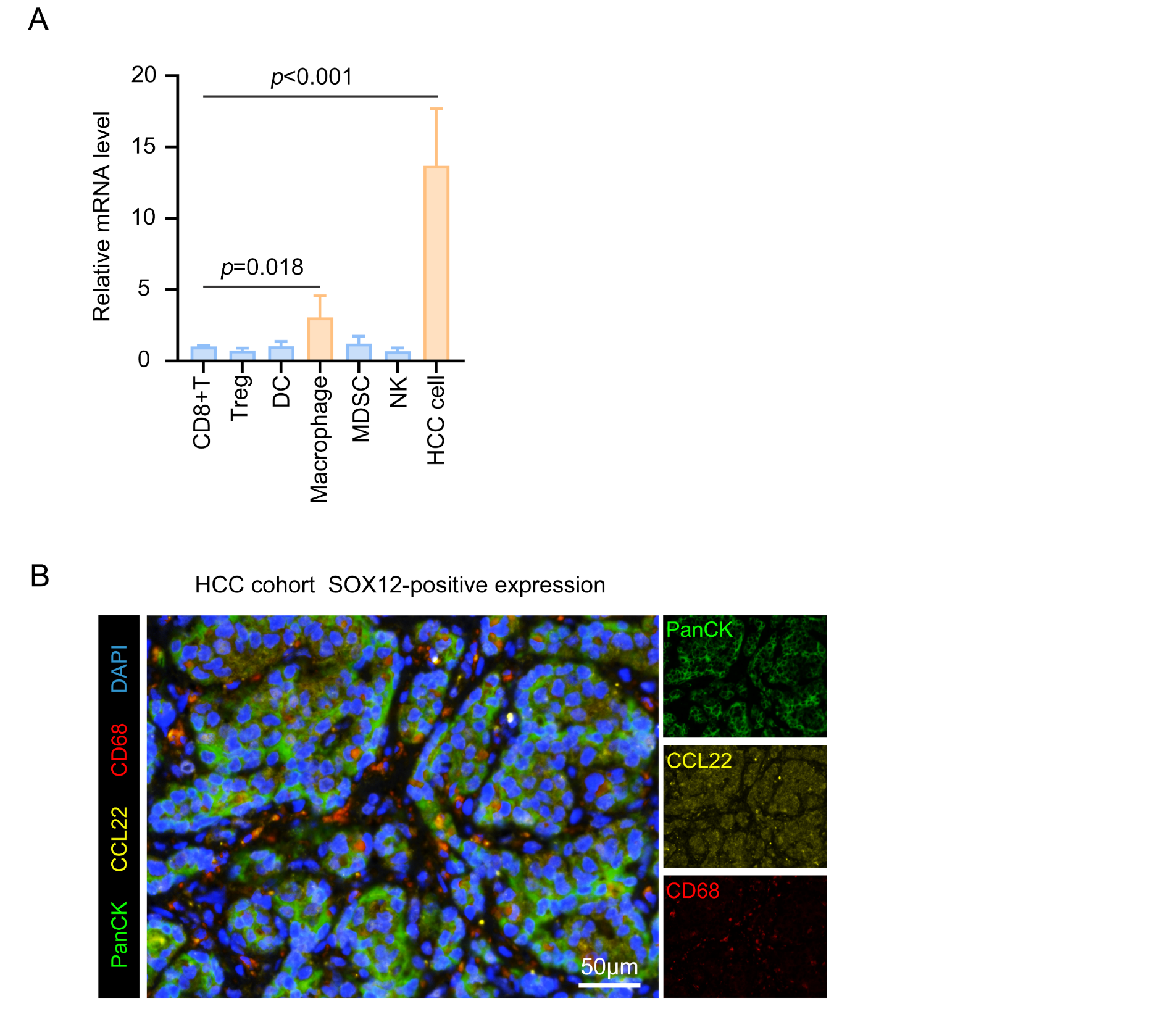
**

**Figure S9**. (A) Flow cytometry was utilized to isolate and characterize various immune cell populations within the liver tumors of mice after the orthotopic injection of Hepa1-6-SOX12 cells for two weeks, including CD8^+^T cells, CD25^+^Foxp3^+^Tregs, F4/80^-^CD11c^+^DCs, F4/80^+^macrophages, CD11b^+^Gr-1^+^MDSCs, and CD3^-^NK1.1^+^NKs. Tumor cells within the mouse liver tumor tissues were isolated using the mouse Tumor Cell Isolation Kit. RT-qPCR was used to detect the level of CCL22 in these immune cells and tumor cells (n=5/group). (B) Multiplex immunofluorescence was performed in the human HCC tissues with SOX12-positive expression, and representative images are presented. Green for tumor cells, yellow for CCL22^+^cells, and red for macrophages.

**
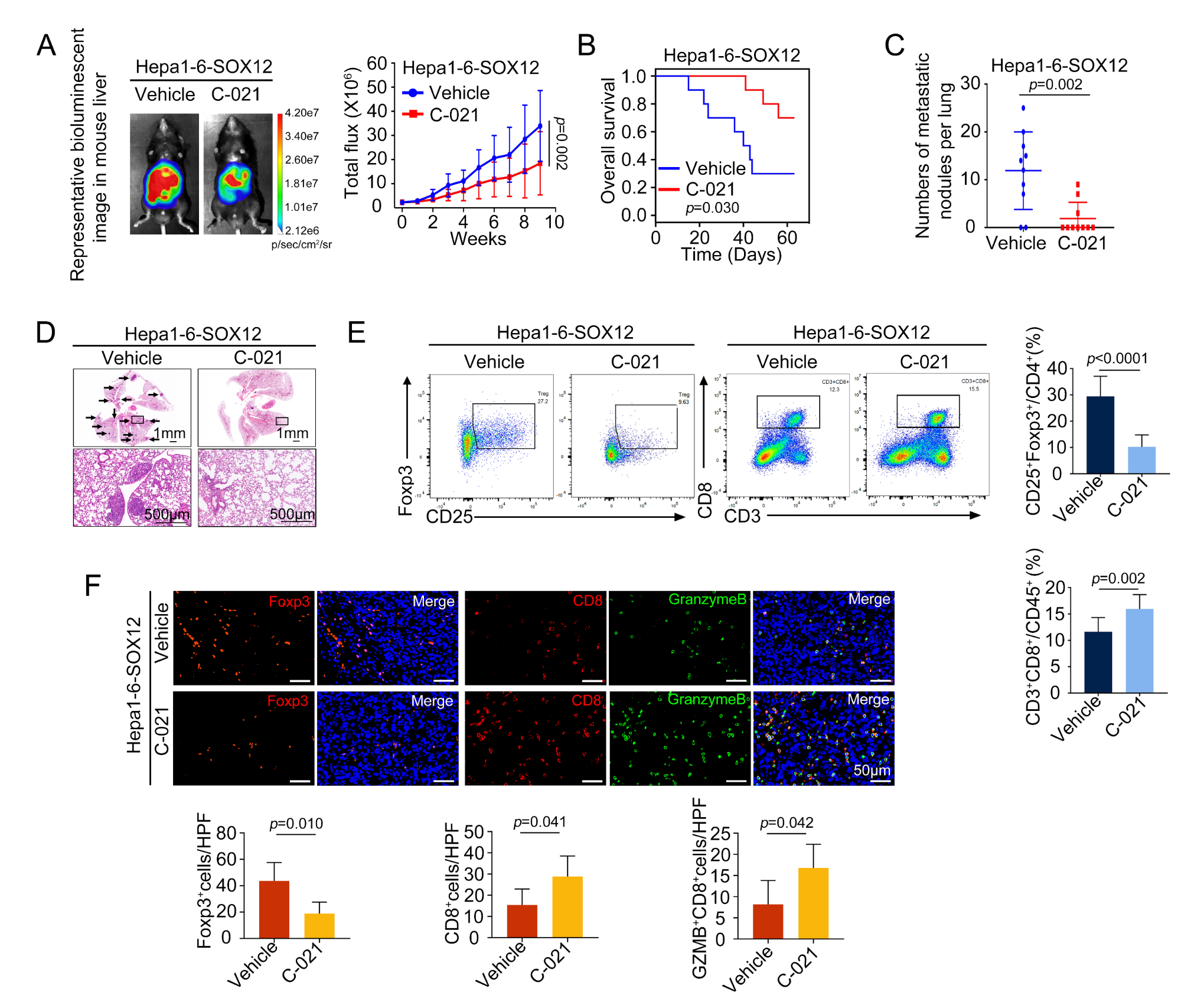
Figure S10**

**Figure S10.** (A-F) Administration of C-021 to Hepa1-6-SOX12 cells-established intrahepatic orthotopic model (n=10/group). (A) The representative bioluminescent images and bioluminescence intensity of tumors, (B) overall survival, (C) lung metastatic nodule numbers, and (D) representative lung H&E staining were shown. (E-F) The intratumoral infiltration of CD25^+^Foxp3^+^Tregs and CD3^+^CD8^+^T cells was analyzed by flow cytometry (E) and immunofluorescent staining (F). For (A), Two-way ANOVA. For (B), Long-rank test. For (C), (E), and (F), Unpaired t-test.

**
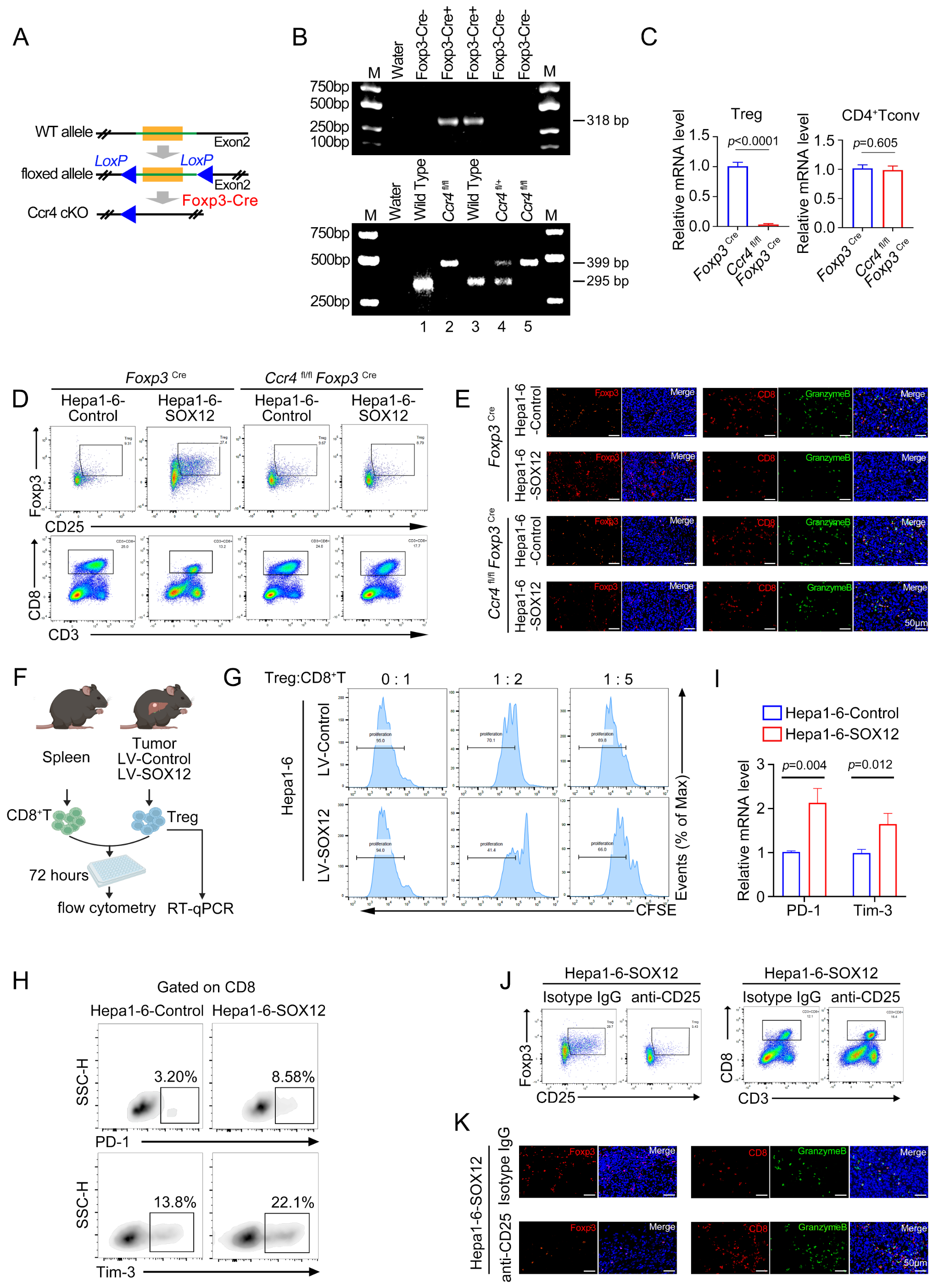
Figure S11**

**Figure S11.** (A) Schematic workflow of generating the Treg-specific CCR4 knockout (*Ccr4*^fl/fl^*Foxp3*^Cre^) mice. (B) Genomic PCR analysis for identifying *Ccr4*^fl/fl^*Foxp3*^Cre^ mice (n=5). (C) The mRNA levels of CCR4 in CD4^+^Foxp3^+^Tregs and CD4^+^Foxp3^-^conventional T cell (Tconv) of Treg-specific CCR4 knockout mice and control mice were detected by RT-qPCR. The significance was determined by Unpaired t-test. (D-E) Representative flow cytometry images (D) and immunofluorescent staining images (E) of intrahepatic Tregs and CD8^+^ T cells in the indicated mice. (F) Schematic diagram of T-cell suppression assay. (G) Representative flow cytometry images of the proliferation of CFSE-labeled CD8^+^T cells. (H) Representative flow cytometry images of PD-1^+^/CD8^+^T cells and Tim-3^+^/CD8^+^T cells in the liver tumors of mice after the orthotopic injection of specific HCC cells for two weeks. (I) RT-qPCR was utilized to detect the mRNA levels of PD-1 and Tim-3 in CD8^+^T cells within the liver tumors of mice after the orthotopic injection of specific HCC cells for two weeks. The significance was determined by Unpaired t-test. (J-K) Representative flow cytometry images (J) and immunofluorescent staining images (K) of intrahepatic Tregs and CD8^+^ T cells in the indicated mice.

**
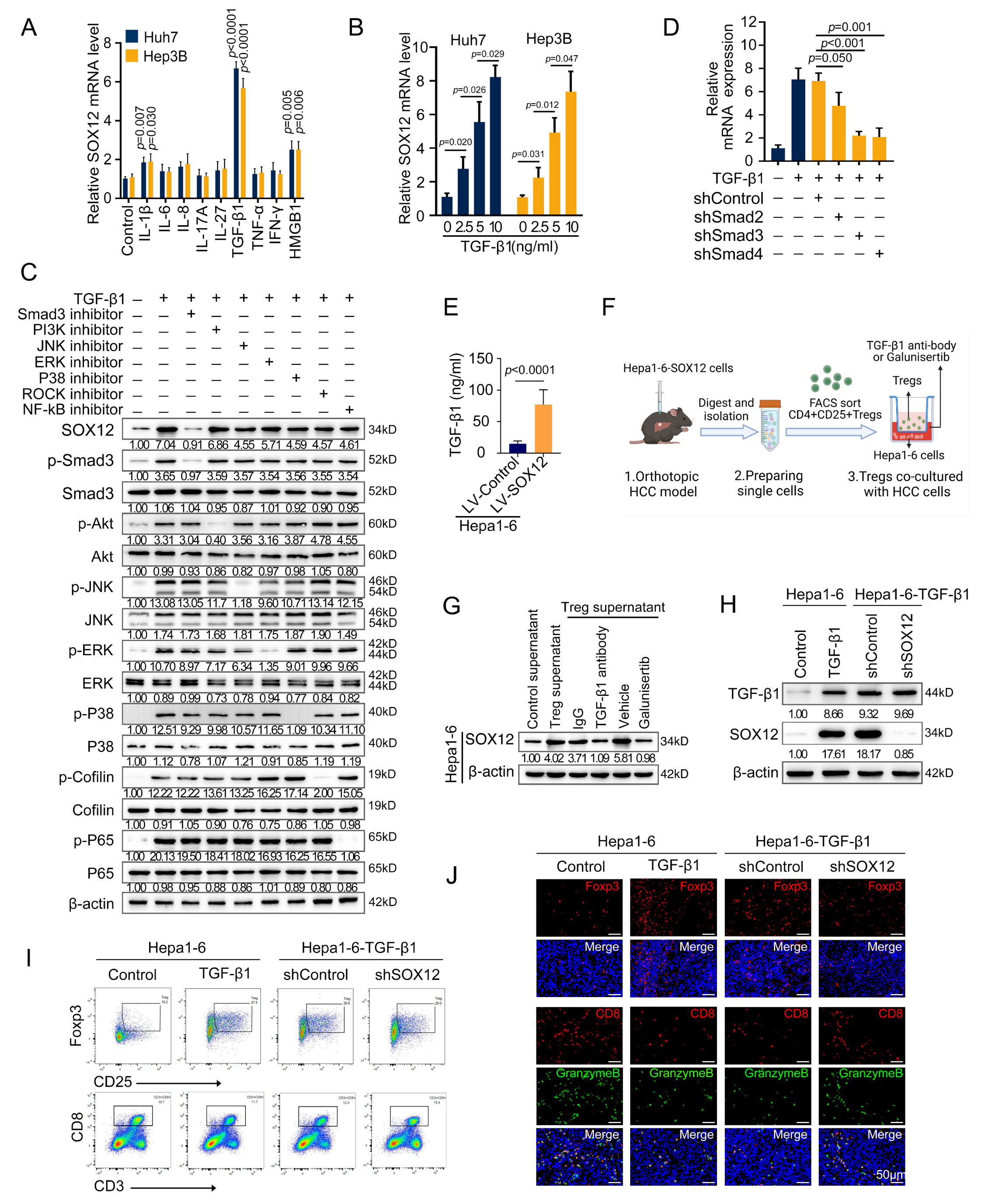
Figure S12**

**Figure S12.** (A) The expression of SOX12 in HCC cells with treatment of cytokines for 24 hours. (B) The expression of SOX12 in HCC cells treated with different concentrations of TGF-β1 for 24 hours was detected. (C) The levels of SOX12 and TGF-β1-regulated molecules in TGF-β1-treated Huh7 cells with the administration of corresponding inhibitors were detected. (D) The levels of SOX12 and Smad2/3/4 in TGF-β1-treated Huh7 cells with Smad2/3/4 knockdown were detected. (E) The secretory level of TGF-β1 in the liver from orthotopic HCC mice bearing Hepa1-6-SOX12/Control cells. (F) Schematic diagram of the isolation, sorting, co-culture, and treatment of Tregs. (G) The expression of SOX12 in Hepa1-6 cells with the administration of anti-TGF-β1 neutralizing antibody (10 μg/ml) or galunisertib (10 µM) for 24 hours. (H) The efficiency of TGF-β1 overexpression and SOX12 knockdown in Hepa1-6 cells was validated. (I-J) Representative flow cytometry images (I) and immunofluorescent staining images (J) of intrahepatic Tregs and CD8^+^ T cells in the indicated mice. For (A), (B), (D), and (E), Unpaired t-test.

**Figure S13**


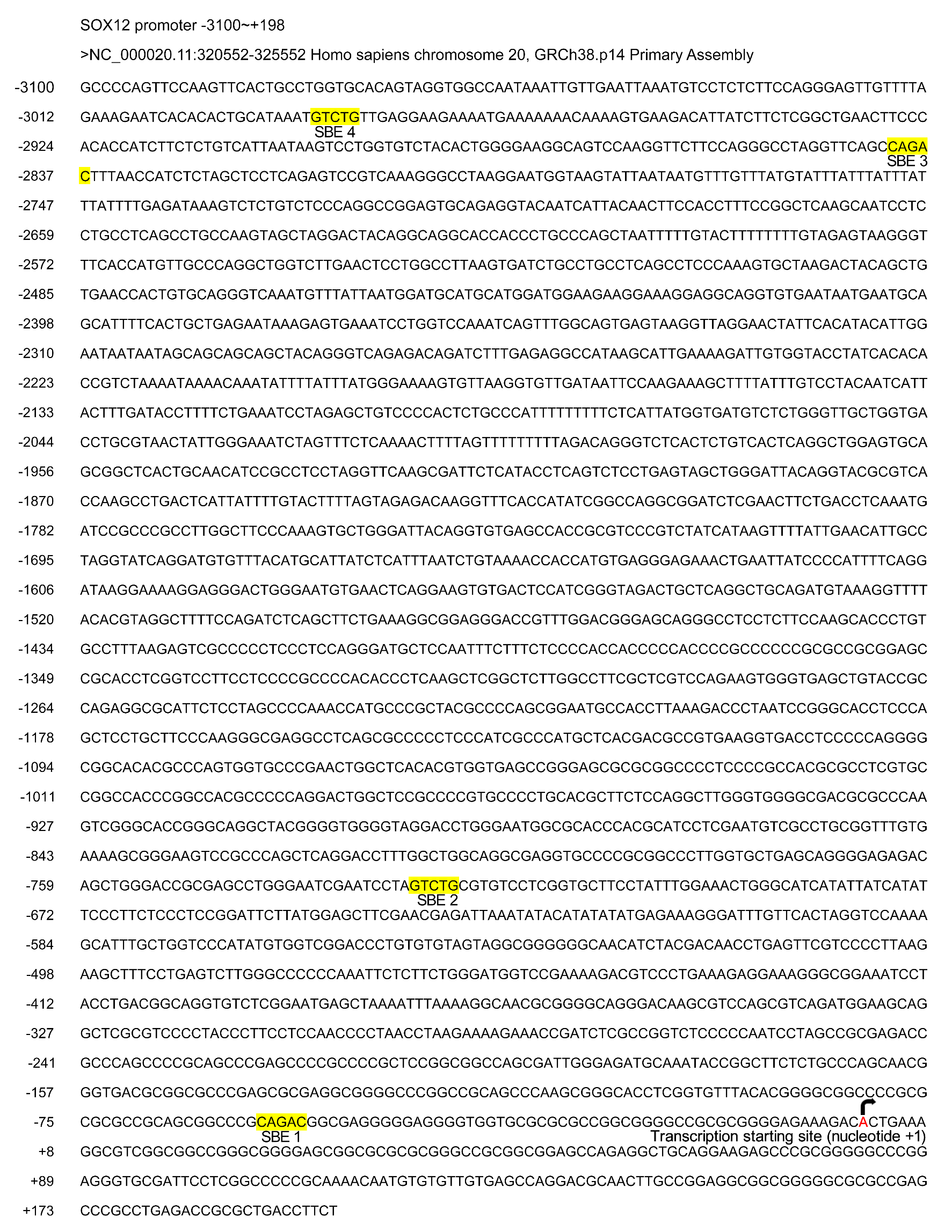
**Figure S13.** The sequence of human *SOX12* promoter. The Smad2/3/4 binding sites (SBE) were highlighted in yellow, and the transcription start site was highlighted in red.


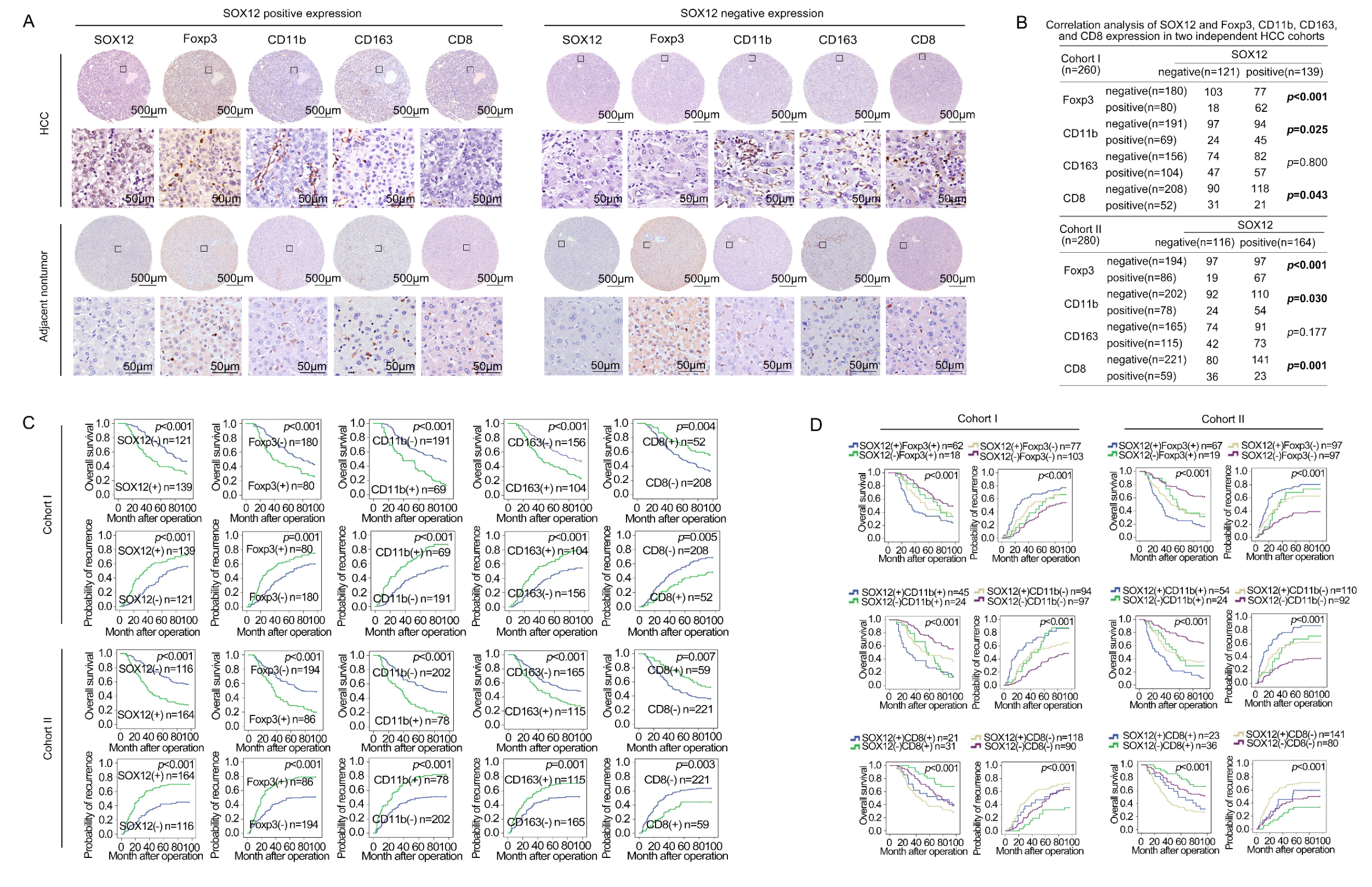
**Figure S14**

**Figure S14**. (A) Representative IHC staining images of SOX12, Foxp3, CD11b, CD163, and CD8 expression in adjacent nontumor tissues and HCC tissues in tissue microarray. The scale bars display 500 μm (low magnification) and 50 μm (high magnification). (B) Association analysis of SOX12 expression and Foxp3, CD11b, CD163, and CD8 expression in two cohorts. (C) Association of the recurrence rate or overall survival time and the expression of SOX12, Foxp3, CD11b, CD163, or CD8 in two cohorts. (D) Association of the recurrence rate or overall survival time and the co-expression of SOX12/Foxp3, SOX12/CD11b, or SOX12/CD8 in two cohorts. For (B), Chi-squared test. For (C) and (D), Long-rank test.


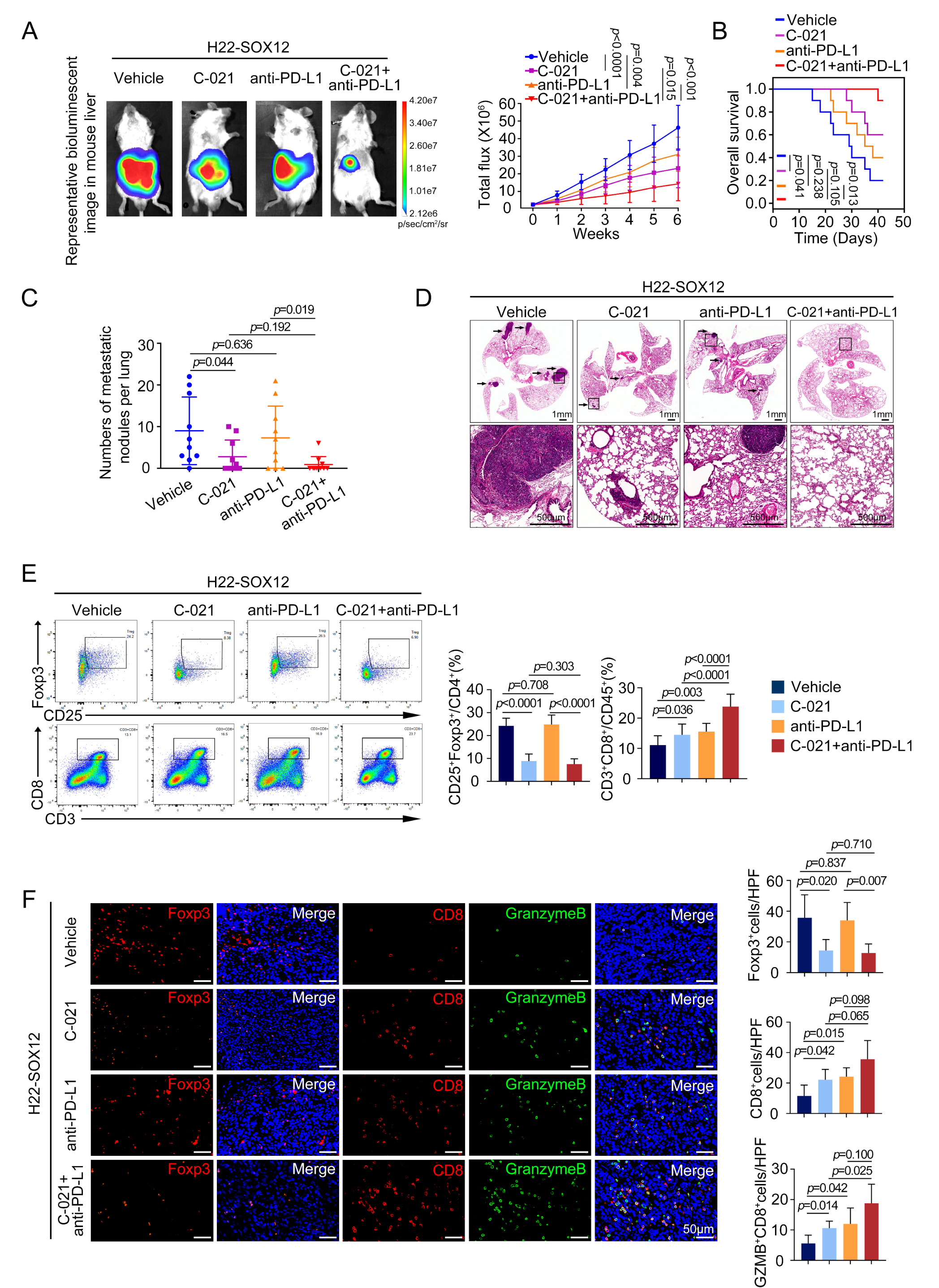
**Figure S15**

**Figure S15**. (A-F) Evaluation of the anti-tumor activity of C-021 in combination with anti-PD-L1 on the intrahepatic orthotopic models bearing H22-SOX12 cells (n=10/group). (A) The representative bioluminescent images and bioluminescence intensity of tumors, (B) overall survival, (C) lung metastatic nodule numbers, and (D) representative lung H&E staining were shown. (E-F) The intratumoral infiltration of Tregs and CD8^+^T cells was analyzed by flow cytometry (E) and immunofluorescent staining (F). For (A), Two-way ANOVA. For (B), Long-rank test. For (C), (E), and (F), Unpaired t-test.


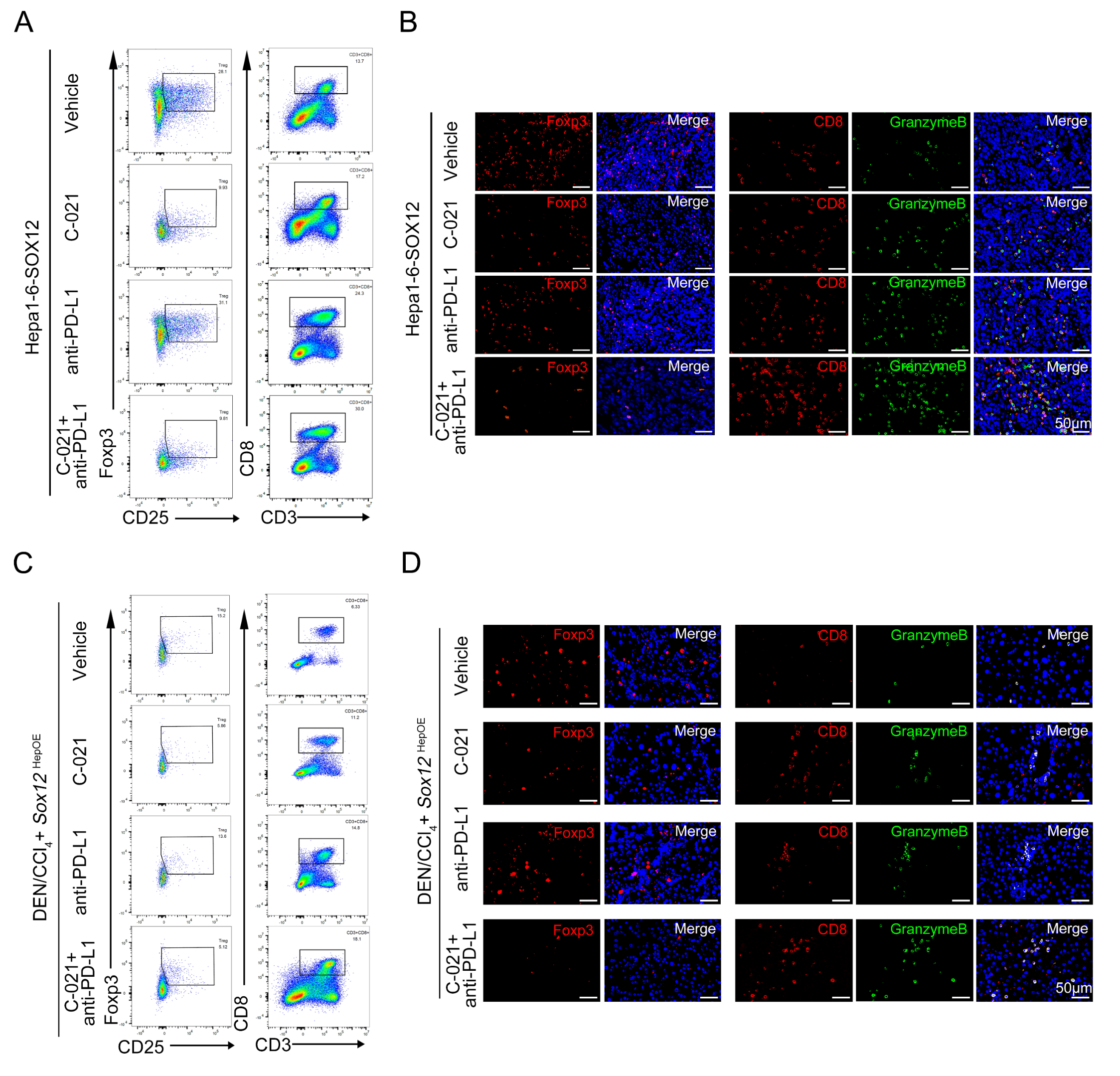
**Figure S16**

**Figure S16.** (A-D) Representative flow cytometry images (A, C) and immunofluorescent staining images (B, D) of intrahepatic Tregs and CD8^+^ T cells in the indicated mice.

**
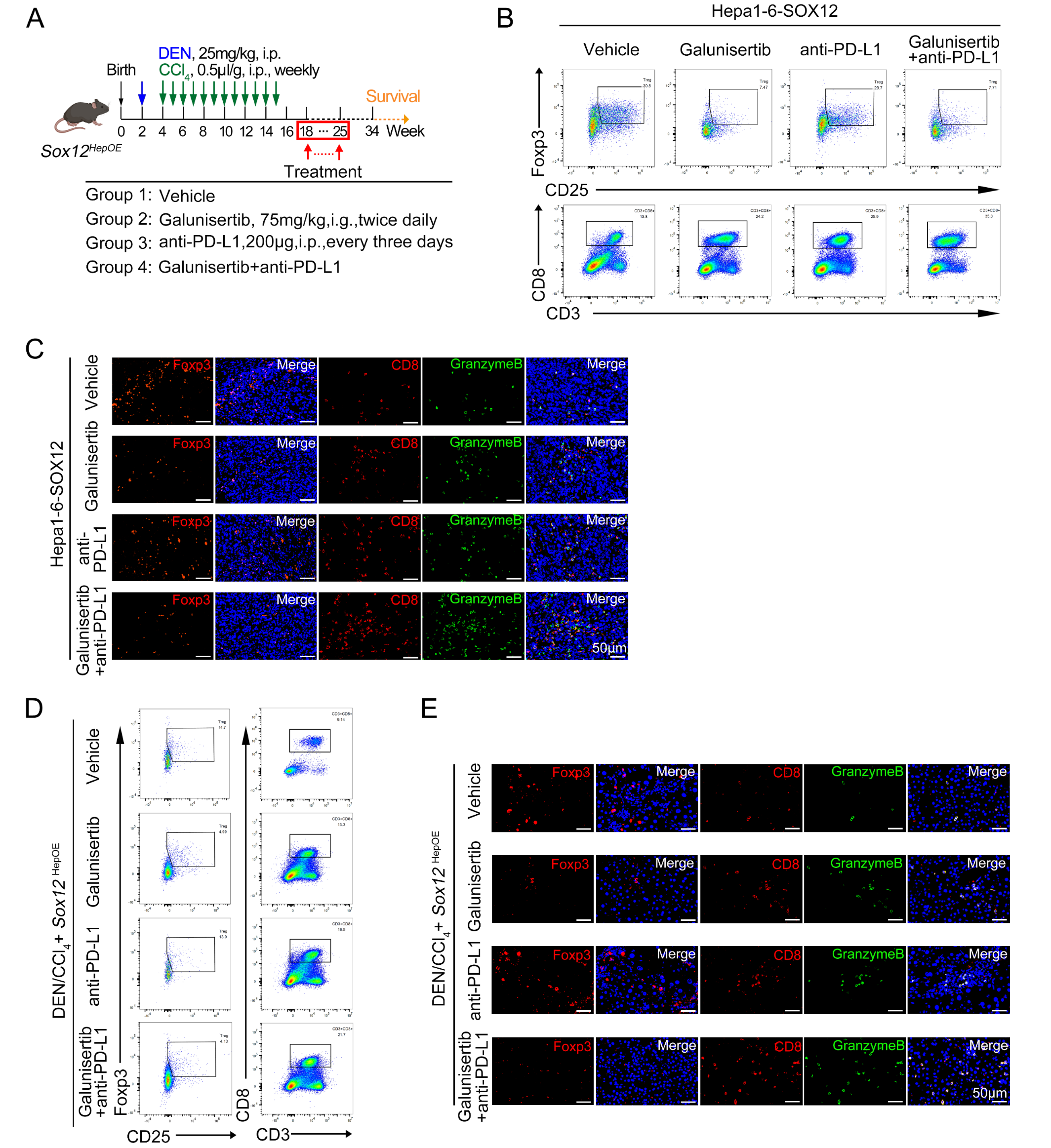
Figure S17**

**Figure S17.** (A) Schematic workflow of evaluating the antitumor effect of galunisertib in combination with anti-PD-L1 on the DEN/CCl_4_-treated *Sox12*^HepOE^ model. (B-E) Representative flow cytometry images (B, D) and immunofluorescent staining images (C, E) of intrahepatic Tregs and CD8^+^ T cells in the indicated mice.


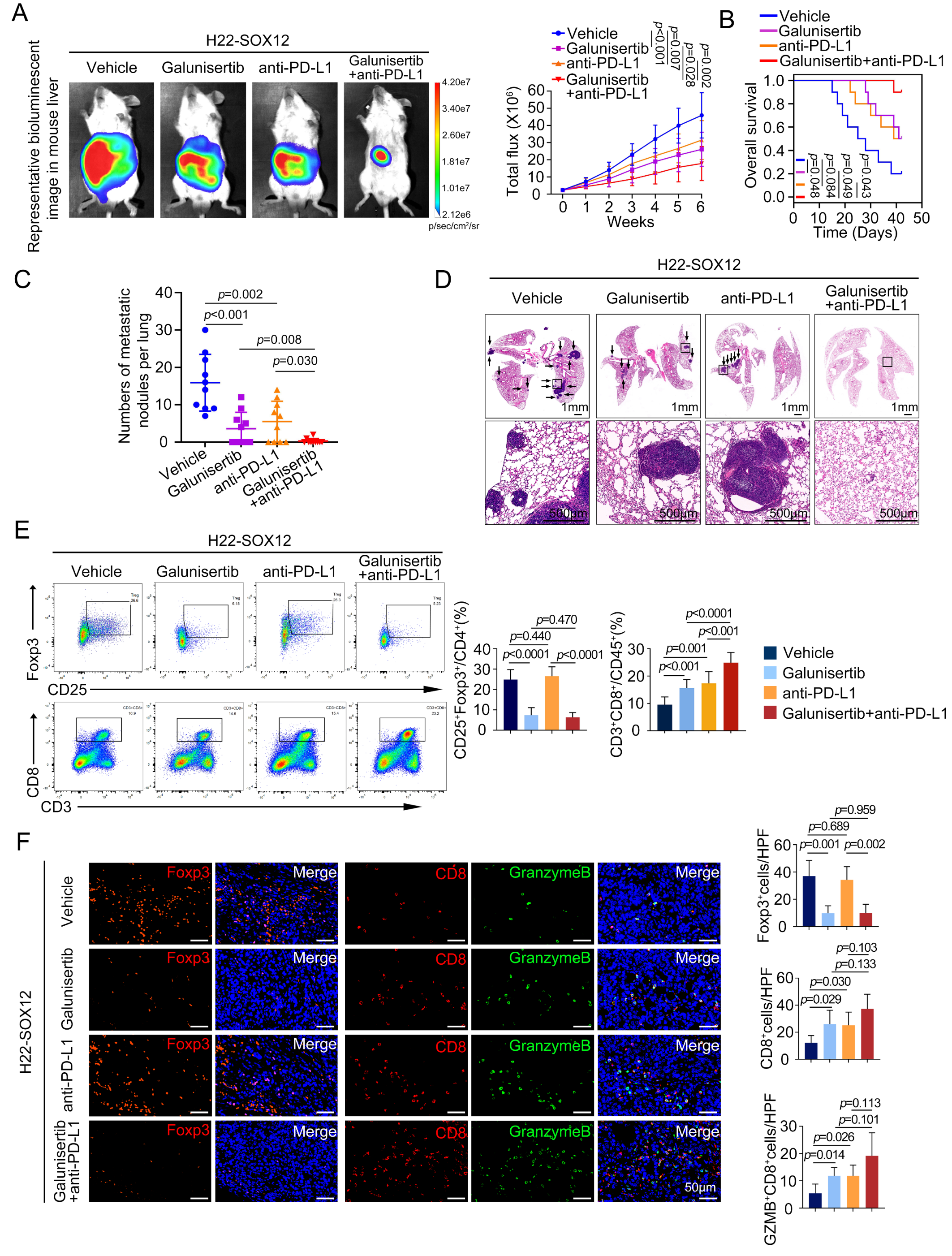
**Figure S18**

**Figure S18**. (A-F) Evaluation of the anti-tumor activity of galunisertib in combination with anti-PD-L1 on the intrahepatic orthotopic models bearing H22-SOX12 cells (n=10/group). (A) The representative bioluminescent images and bioluminescence intensity of tumors, (B) overall survival, (C) lung metastatic nodule numbers, and (D) representative lung H&E staining were shown. (E-F) The intratumoral infiltration of Tregs and CD8^+^T cells was analyzed by flow cytometry (E) and immunofluorescent staining (F). For (A), Two-way ANOVA. For (B), Long-rank test. For (C), (E), and (F), Unpaired t-test.

**Supplementary Tables**

**Table S1**. List of genes differentially expressed in Huh7-SOX12 versus Huh7-Control cells using RNA-seq.

| genes | baseMean | log2FoldChange | p.value | p.adj |
| --- | --- | --- | --- | --- |
| RPS28P7 | 35.71 | 7.57 | 3.07E-12 | 1.85E-11 |
| SLITRK1 | 18.32 | 6.52 | 2.02E-09 | 9.71E-09 |
| MICOS10-NBL1 | 17.86 | 6.46 | 3.20E-09 | 1.51E-08 |
| BGN | 67.71 | 6.23 | 6.82E-18 | 6.12E-17 |
| NPPB | 602.29 | 6.13 | 1.24E-161 | 3.40E-159 |
| DCX | 69.26 | 6.01 | 4.54E-20 | 4.63E-19 |
| MAMDC2 | 12.58 | 5.81 | 7.08E-08 | 2.94E-07 |
| KISS1 | 11.26 | 5.58 | 1.85E-07 | 7.39E-07 |
| SPDYE2B | 10.27 | 5.29 | 7.08E-07 | 2.66E-06 |
| LIN28A | 54.08 | 5.17 | 1.81E-18 | 1.69E-17 |
| GSTP1 | 8.90 | 5.04 | 1.30E-06 | 4.75E-06 |
| TMSB15A | 46.87 | 4.94 | 2.52E-17 | 2.17E-16 |
| CD24 | 100.32 | 4.48 | 9.19E-36 | 2.00E-34 |
| DOCK2 | 82.60 | 4.46 | 8.15E-31 | 1.44E-29 |
| TNNT2 | 345.92 | 4.35 | 2.91E-116 | 4.66E-114 |
| VTCN1 | 10.84 | 4.15 | 9.35E-06 | 3.11E-05 |
| FXYD6 | 114.74 | 4.12 | 3.94E-40 | 1.07E-38 |
| IL11 | 19.58 | 4.10 | 2.25E-08 | 9.80E-08 |
| NYNRIN | 16.62 | 4.06 | 3.18E-07 | 1.24E-06 |
| ZNF676 | 11.03 | 4.04 | 1.45E-05 | 4.71E-05 |
| PCSK2 | 6.22 | 3.95 | 2.27E-05 | 7.20E-05 |
| SMO | 6.21 | 3.92 | 2.51E-05 | 7.90E-05 |
| TIE1 | 66.94 | 3.83 | 5.13E-24 | 6.41E-23 |
| MEX3A | 988.49 | 3.72 | 4.49E-277 | 4.12E-274 |
| ABI3 | 16.38 | 3.72 | 3.68E-07 | 1.43E-06 |
| GAGE12F | 58.92 | 3.62 | 5.99E-21 | 6.39E-20 |
| MYLK2 | 42.91 | 3.61 | 5.06E-15 | 3.76E-14 |
| NELL2 | 96.81 | 3.60 | 5.77E-33 | 1.12E-31 |
| B3GALT5 | 33.03 | 3.60 | 1.59E-12 | 9.79E-12 |
| FGF1 | 794.98 | 3.60 | 3.02E-213 | 1.47E-210 |
| EMILIN2 | 269.06 | 3.59 | 1.68E-59 | 9.12E-58 |
| PLGLB2 | 5.59 | 3.53 | 5.40E-05 | 1.63E-04 |
| PEAR1 | 50.90 | 3.53 | 2.10E-18 | 1.95E-17 |
| MYOCD | 5.55 | 3.53 | 5.46E-05 | 1.65E-04 |
| PGM5P2 | 8.88 | 3.52 | 4.11E-05 | 1.26E-04 |
| NTN1 | 27.59 | 3.52 | 1.10E-10 | 5.89E-10 |
| PBDC1 | 11.66 | 3.50 | 2.01E-05 | 6.42E-05 |
| SYTL5 | 13.09 | 3.49 | 5.65E-06 | 1.93E-05 |
| FRMPD3 | 22.18 | 3.46 | 4.01E-09 | 1.87E-08 |
| SSX2B | 57.55 | 3.45 | 2.70E-19 | 2.66E-18 |
| MARCKSL1 | 456.96 | 3.38 | 1.42E-107 | 2.00E-105 |
| MARCHF4 | 625.49 | 3.33 | 1.63E-169 | 4.90E-167 |
| CDH5 | 8.30 | 3.33 | 6.02E-05 | 1.81E-04 |
| CICP21 | 35.88 | 3.33 | 2.78E-13 | 1.81E-12 |
| VCAN | 8.28 | 3.33 | 6.05E-05 | 1.82E-04 |
| PLEKHA6 | 41.83 | 3.31 | 6.25E-14 | 4.27E-13 |
| CORO1A | 26.01 | 3.28 | 1.11E-09 | 5.47E-09 |
| RTL8B | 16.95 | 3.27 | 4.31E-07 | 1.66E-06 |
| AOX1 | 78.49 | 3.27 | 7.85E-26 | 1.08E-24 |
| VGLL2 | 12.03 | 3.25 | 1.55E-05 | 5.02E-05 |
| FAT3 | 14.67 | 3.21 | 2.45E-06 | 8.69E-06 |
| DKK1 | 78.77 | 3.20 | 8.61E-08 | 3.55E-07 |
| KLHL26 | 14.42 | 3.20 | 1.77E-06 | 6.40E-06 |
| SYT16 | 5.09 | 3.19 | 1.01E-04 | 2.95E-04 |
| LOXL1 | 16.05 | 3.17 | 8.00E-07 | 2.99E-06 |
| OR7E19P | 12.49 | 3.14 | 6.90E-06 | 2.33E-05 |
| MEGF10 | 39.86 | 3.11 | 1.51E-13 | 1.00E-12 |
| KCNH2 | 64.92 | 3.11 | 8.92E-21 | 9.39E-20 |
| CDH8 | 268.16 | 3.09 | 6.52E-73 | 4.98E-71 |
| PAGE5 | 21.45 | 3.07 | 4.18E-08 | 1.77E-07 |
| EDNRB | 9.91 | 3.06 | 6.26E-05 | 1.88E-04 |
| PLEKHA7 | 1253.91 | 3.05 | 5.08E-232 | 3.00E-229 |
| C13orf42 | 38.16 | 3.05 | 1.95E-13 | 1.29E-12 |
| TNNI1 | 13.88 | 3.04 | 5.70E-06 | 1.94E-05 |
| MYO7B | 13.49 | 3.04 | 4.53E-06 | 1.56E-05 |
| ZNF354C | 19.21 | 3.03 | 1.05E-07 | 4.30E-07 |
| SP140 | 100.58 | 3.02 | 1.72E-30 | 3.00E-29 |
| COL8A1 | 321.16 | 2.98 | 9.65E-81 | 8.34E-79 |
| APOE | 167.81 | 2.98 | 8.47E-44 | 2.64E-42 |
| ADAMTSL1 | 33.16 | 2.95 | 2.36E-11 | 1.33E-10 |
| CADM4 | 967.98 | 2.93 | 1.14E-185 | 4.02E-183 |
| PAPPA | 52.58 | 2.93 | 1.77E-14 | 1.26E-13 |
| ZNF208 | 63.40 | 2.88 | 6.12E-19 | 5.91E-18 |
| CICP3 | 7.36 | 2.85 | 1.60E-04 | 4.52E-04 |
| TOX | 43.91 | 2.80 | 1.59E-13 | 1.05E-12 |
| GPC2 | 49.33 | 2.79 | 3.37E-15 | 2.53E-14 |
| MAGEB6 | 4.60 | 2.77 | 2.16E-04 | 5.99E-04 |
| THBS1 | 3043.10 | 2.72 | 0.00E+00 | 0.00E+00 |
| SALL4 | 11.96 | 2.70 | 2.57E-05 | 8.07E-05 |
| WNT7B | 155.50 | 2.69 | 1.56E-33 | 3.11E-32 |
| HRNR | 7.05 | 2.68 | 2.21E-04 | 6.12E-04 |
| TMSB4XP4 | 60.07 | 2.67 | 2.73E-16 | 2.22E-15 |
| TNS1 | 162.85 | 2.67 | 1.50E-32 | 2.87E-31 |
| RPL7AP22 | 18.76 | 2.65 | 7.24E-07 | 2.71E-06 |
| COMMD3-BMI1 | 9.20 | 2.65 | 1.75E-04 | 4.93E-04 |
| UHRF1 | 3583.17 | 2.62 | 0.00E+00 | 0.00E+00 |
| CX3CL1 | 35.28 | 2.61 | 1.81E-10 | 9.49E-10 |
| MYL4 | 4.45 | 2.59 | 2.97E-04 | 8.11E-04 |
| F2RL2 | 6.71 | 2.58 | 2.66E-04 | 7.31E-04 |
| CDK5R1 | 98.76 | 2.58 | 5.56E-23 | 6.64E-22 |
| ZNF518B | 19.47 | 2.57 | 9.08E-07 | 3.37E-06 |
| GRPR | 51.69 | 2.56 | 7.50E-14 | 5.09E-13 |
| CICP16 | 20.88 | 2.54 | 2.56E-07 | 1.01E-06 |
| IGFBP4 | 34.72 | 2.53 | 3.15E-10 | 1.62E-09 |
| GAGE2A | 166.67 | 2.51 | 3.68E-36 | 8.24E-35 |
| KIAA1549L | 49.56 | 2.51 | 1.77E-12 | 1.09E-11 |
| KRT17 | 272.94 | 2.51 | 1.98E-60 | 1.10E-58 |
| ZNF804A | 8.80 | 2.49 | 1.42E-04 | 4.07E-04 |
| CD37 | 116.02 | 2.48 | 3.12E-25 | 4.16E-24 |
| ENC1 | 1202.89 | 2.45 | 1.46E-162 | 4.10E-160 |
| RPS8P5 | 4.14 | 2.44 | 3.82E-04 | 1.03E-03 |
| ENG | 119.93 | 2.40 | 5.42E-27 | 8.04E-26 |
| ADAM33 | 4.29 | 2.39 | 4.23E-04 | 1.13E-03 |
| LRRC15 | 8.65 | 2.39 | 1.93E-04 | 5.39E-04 |
| STYXL2 | 10.51 | 2.37 | 9.04E-05 | 2.65E-04 |
| MYRF | 328.59 | 2.36 | 5.38E-57 | 2.64E-55 |
| TWIST1 | 72.83 | 2.34 | 9.62E-16 | 7.51E-15 |
| CDC27P1 | 31.65 | 2.33 | 1.44E-08 | 6.37E-08 |
| ITIH3 | 115.47 | 2.33 | 1.05E-24 | 1.38E-23 |
| LZTS1 | 146.81 | 2.30 | 1.62E-24 | 2.10E-23 |
| EDN1 | 160.31 | 2.29 | 9.39E-32 | 1.73E-30 |
| GPR75 | 68.15 | 2.29 | 2.30E-14 | 1.63E-13 |
| MAGEB17 | 132.19 | 2.28 | 1.40E-26 | 2.03E-25 |
| EXOC3L2 | 9.55 | 2.27 | 2.00E-04 | 5.59E-04 |
| PTGFRN | 3.99 | 2.26 | 5.23E-04 | 1.38E-03 |
| COL5A3 | 314.30 | 2.25 | 4.29E-45 | 1.40E-43 |
| EPS8L3 | 7.88 | 2.25 | 4.08E-04 | 1.09E-03 |
| F2R | 1846.54 | 2.25 | 7.85E-196 | 3.09E-193 |
| RASEF | 360.69 | 2.24 | 4.18E-60 | 2.32E-58 |
| KIAA0408 | 3.99 | 2.24 | 5.41E-04 | 1.42E-03 |
| TLL1 | 7.65 | 2.24 | 4.14E-04 | 1.11E-03 |
| UCN2 | 18.48 | 2.24 | 4.10E-06 | 1.42E-05 |
| MYL7 | 8.50 | 2.23 | 3.02E-04 | 8.23E-04 |
| SPSB4 | 151.34 | 2.23 | 3.36E-29 | 5.56E-28 |
| BTNL12P | 42.13 | 2.22 | 2.66E-09 | 1.27E-08 |
| ALPP | 273.04 | 2.22 | 5.18E-40 | 1.39E-38 |
| F2RL1 | 9.78 | 2.20 | 1.48E-04 | 4.22E-04 |
| AWAT2 | 9.89 | 2.20 | 1.64E-04 | 4.64E-04 |
| UNC5B | 52.88 | 2.20 | 2.19E-11 | 1.24E-10 |
| GAGE12J | 165.41 | 2.18 | 5.50E-31 | 9.78E-30 |
| CPA4 | 98.98 | 2.17 | 5.21E-19 | 5.04E-18 |
| ADAM19 | 416.15 | 2.17 | 7.67E-62 | 4.40E-60 |
| RELN | 17.04 | 2.17 | 9.52E-06 | 3.16E-05 |
| FLT1 | 12.88 | 2.17 | 7.07E-05 | 2.10E-04 |
| UROC1 | 3.83 | 2.15 | 6.42E-04 | 1.67E-03 |
| NES | 1002.68 | 2.15 | 5.55E-91 | 5.72E-89 |
| TIMP3 | 809.84 | 2.15 | 8.43E-103 | 1.07E-100 |
| SERHL2 | 52.73 | 2.14 | 7.46E-12 | 4.38E-11 |
| TFCP2L1 | 5.73 | 2.13 | 6.33E-04 | 1.65E-03 |
| HNF4A | 33.26 | 2.12 | 3.30E-08 | 1.41E-07 |
| ACTG2 | 464.33 | 2.11 | 2.31E-67 | 1.57E-65 |
| PAX6 | 12.78 | 2.11 | 1.05E-04 | 3.05E-04 |
| SOX12 | 3091.54 | 2.09 | 5.07E-232 | 3.00E-229 |
| EPPK1 | 1878.38 | 2.08 | 1.71E-141 | 3.76E-139 |
| BGLAP | 3.97 | 2.06 | 7.60E-04 | 1.95E-03 |
| CRISPLD2 | 60.61 | 2.06 | 3.18E-12 | 1.92E-11 |
| RPL13AP20 | 49.70 | 2.06 | 6.38E-11 | 3.48E-10 |
| HMGA2 | 559.77 | 2.06 | 1.69E-81 | 1.50E-79 |
| LSM12P1 | 120.39 | 2.06 | 1.11E-15 | 8.65E-15 |
| SMAD6 | 811.88 | 2.04 | 1.67E-56 | 8.00E-55 |
| TET3 | 490.82 | 2.04 | 1.13E-61 | 6.44E-60 |
| SOX5 | 21.77 | 2.03 | 6.60E-06 | 2.24E-05 |
| EIF5AL1 | 14.50 | 2.03 | 4.52E-05 | 1.38E-04 |
| GLS | 556.79 | 2.02 | 1.99E-78 | 1.67E-76 |
| CYP26A1 | 3.66 | 2.01 | 8.24E-04 | 2.11E-03 |
| NOSTRIN | 9.56 | 2.01 | 3.27E-04 | 8.86E-04 |
| RAB3B | 351.54 | 2.01 | 5.20E-48 | 1.86E-46 |
| GNG13 | 3.65 | 2.00 | 8.47E-04 | 2.16E-03 |
| CAPN6 | 75.00 | 2.00 | 3.62E-14 | 2.52E-13 |
| LYPD1 | 35.71 | 1.99 | 2.97E-08 | 1.27E-07 |
| B4GALNT3 | 73.81 | 1.99 | 3.19E-14 | 2.23E-13 |
| SLC6A16 | 33.04 | 1.99 | 1.01E-07 | 4.12E-07 |
| SLAMF7 | 46.52 | 1.98 | 6.77E-09 | 3.10E-08 |
| TRIM54 | 251.04 | 1.96 | 3.92E-31 | 7.03E-30 |
| MFSD14CP | 30.77 | 1.95 | 2.66E-07 | 1.05E-06 |
| GLIPR1 | 800.48 | 1.95 | 8.55E-103 | 1.08E-100 |
| GPM6B | 33.56 | 1.92 | 8.58E-08 | 3.54E-07 |
| DUSP9 | 61.21 | 1.92 | 9.29E-11 | 5.00E-10 |
| CTNND2 | 11.04 | 1.92 | 2.91E-04 | 7.94E-04 |
| NXPH2 | 8.37 | 1.92 | 5.39E-04 | 1.41E-03 |
| KIF17 | 37.38 | 1.92 | 1.76E-07 | 7.02E-07 |
| SYCE1 | 22.83 | 1.91 | 4.30E-06 | 1.49E-05 |
| REEP1 | 54.55 | 1.91 | 5.10E-10 | 2.58E-09 |
| THSD7B | 25.06 | 1.90 | 5.06E-06 | 1.73E-05 |
| CD163L1 | 30.84 | 1.90 | 3.05E-07 | 1.19E-06 |
| INSYN2B | 119.44 | 1.90 | 3.53E-18 | 3.23E-17 |
| GAGE13 | 20.41 | 1.90 | 1.27E-05 | 4.15E-05 |
| TSSK5P | 15.48 | 1.90 | 5.42E-05 | 1.64E-04 |
| POU6F2 | 58.25 | 1.89 | 6.17E-11 | 3.37E-10 |
| SEMA3E | 24.55 | 1.89 | 5.33E-06 | 1.82E-05 |
| ANO2 | 138.36 | 1.88 | 1.07E-21 | 1.18E-20 |
| CCND1 | 1719.67 | 1.87 | 2.02E-141 | 4.38E-139 |
| ADRA2A | 22.66 | 1.87 | 8.63E-06 | 2.88E-05 |
| OXTR | 66.60 | 1.86 | 1.66E-10 | 8.74E-10 |
| HNRNPL | 3625.02 | 1.86 | 9.00E-22 | 1.01E-20 |
| SHANK3 | 42.48 | 1.85 | 5.10E-08 | 2.15E-07 |
| CCL22 | 346.16 | 1.85 | 3.36E-30 | 5.79E-29 |
| MATN2 | 1274.41 | 1.84 | 1.16E-110 | 1.72E-108 |
| TRIM58 | 3357.97 | 1.84 | 1.23E-110 | 1.80E-108 |
| SLC7A7 | 32.52 | 1.84 | 4.42E-07 | 1.70E-06 |
| TERT | 160.93 | 1.84 | 2.05E-23 | 2.49E-22 |
| SYTL2 | 70.99 | 1.83 | 2.57E-12 | 1.56E-11 |
| BMP4 | 379.27 | 1.83 | 2.26E-49 | 8.34E-48 |
| ADAMTS17 | 41.64 | 1.82 | 3.30E-08 | 1.41E-07 |
| DBN1 | 4955.95 | 1.82 | 1.38E-217 | 7.58E-215 |
| FAM124A | 174.87 | 1.80 | 5.80E-24 | 7.22E-23 |
| TAGLN | 8639.65 | 1.80 | 9.55E-280 | 9.28E-277 |
| TP53I11 | 766.00 | 1.79 | 2.87E-85 | 2.72E-83 |
| VCX | 135.69 | 1.79 | 2.84E-19 | 2.79E-18 |
| SDC1 | 260.38 | 1.79 | 2.73E-29 | 4.52E-28 |
| ACTC1 | 56.69 | 1.79 | 2.32E-07 | 9.16E-07 |
| ADRB2 | 65.94 | 1.79 | 2.85E-10 | 1.47E-09 |
| CDC25A | 804.10 | 1.78 | 6.67E-88 | 6.59E-86 |
| FOXR2 | 203.33 | 1.77 | 4.97E-24 | 6.22E-23 |
| PHRF1 | 77.39 | 1.77 | 2.05E-08 | 8.94E-08 |
| SYK | 146.05 | 1.76 | 2.39E-18 | 2.22E-17 |
| ZEB2 | 328.52 | 1.76 | 8.38E-28 | 1.30E-26 |
| CREB5 | 118.93 | 1.76 | 3.64E-17 | 3.10E-16 |
| PTPN6 | 3.49 | 1.75 | 1.36E-03 | 3.37E-03 |
| CDC27P3 | 6.58 | 1.75 | 1.28E-03 | 3.17E-03 |
| LPAL2 | 7.69 | 1.75 | 8.68E-04 | 2.21E-03 |
| RPSAP52 | 7.86 | 1.74 | 8.96E-04 | 2.28E-03 |
| PPP1R14A | 3.34 | 1.74 | 1.39E-03 | 3.42E-03 |
| TNC | 745.46 | 1.74 | 5.97E-65 | 3.72E-63 |
| CCNJL | 442.71 | 1.74 | 5.29E-51 | 2.04E-49 |
| EFNA2 | 32.72 | 1.74 | 8.56E-07 | 3.18E-06 |
| SSX3 | 4.94 | 1.73 | 1.42E-03 | 3.50E-03 |
| PDGFRB | 90.89 | 1.73 | 3.30E-13 | 2.13E-12 |
| MARCKS | 2607.53 | 1.72 | 3.70E-163 | 1.07E-160 |
| EFR3B | 309.55 | 1.72 | 5.73E-37 | 1.33E-35 |
| SPRN | 97.91 | 1.71 | 4.43E-13 | 2.83E-12 |
| DHRS2 | 41.88 | 1.71 | 3.48E-07 | 1.35E-06 |
| RPSA2 | 551.59 | 1.70 | 6.77E-60 | 3.75E-58 |
| PMEPA1 | 147.03 | 1.70 | 1.44E-18 | 1.35E-17 |
| POLR1G | 1682.59 | 1.69 | 9.86E-126 | 1.83E-123 |
| SHISAL1 | 120.10 | 1.69 | 5.43E-16 | 4.33E-15 |
| SDK1 | 40.41 | 1.69 | 4.13E-07 | 1.59E-06 |
| VGLL3 | 61.51 | 1.69 | 6.98E-10 | 3.48E-09 |
| RPL12P38 | 6.40 | 1.69 | 1.47E-03 | 3.62E-03 |
| MAMLD1 | 366.32 | 1.68 | 3.84E-39 | 9.89E-38 |
| STXBP5L | 13.95 | 1.68 | 2.90E-04 | 7.92E-04 |
| ZFTA | 680.33 | 1.67 | 4.27E-66 | 2.79E-64 |
| RASSF2 | 112.71 | 1.67 | 6.48E-15 | 4.77E-14 |
| HS3ST5 | 20.12 | 1.67 | 3.90E-05 | 1.20E-04 |
| GNG2 | 14.95 | 1.67 | 1.63E-04 | 4.61E-04 |
| CCDC86 | 1413.60 | 1.67 | 1.89E-100 | 2.35E-98 |
| ZNF850 | 654.23 | 1.66 | 3.41E-65 | 2.14E-63 |
| ENAM | 14.94 | 1.66 | 1.70E-04 | 4.78E-04 |
| MEX3B | 405.86 | 1.66 | 3.60E-45 | 1.17E-43 |
| CICP27 | 171.27 | 1.66 | 2.22E-20 | 2.29E-19 |
| TRPC4 | 75.81 | 1.65 | 2.17E-10 | 1.13E-09 |
| MBL1P | 13.52 | 1.64 | 4.34E-04 | 1.15E-03 |
| TUBB3 | 1420.28 | 1.64 | 2.39E-104 | 3.13E-102 |
| LSP1P4 | 173.28 | 1.63 | 1.32E-20 | 1.38E-19 |
| NCOA5 | 924.71 | 1.62 | 4.34E-86 | 4.24E-84 |
| SHISA9 | 288.91 | 1.62 | 1.34E-31 | 2.44E-30 |
| PET100 | 150.30 | 1.61 | 1.27E-18 | 1.20E-17 |
| PCSK9 | 50.41 | 1.61 | 1.62E-07 | 6.49E-07 |
| SLFN12 | 305.55 | 1.60 | 3.36E-33 | 6.53E-32 |
| ZFHX4 | 55.02 | 1.60 | 4.49E-08 | 1.90E-07 |
| PLK2 | 758.91 | 1.60 | 1.01E-59 | 5.52E-58 |
| ZNF334 | 3.17 | 1.60 | 1.87E-03 | 4.51E-03 |
| MYB | 48.16 | 1.60 | 1.23E-07 | 4.98E-07 |
| H2AB3 | 9.01 | 1.59 | 1.04E-03 | 2.63E-03 |
| ANKRD2 | 76.60 | 1.59 | 3.06E-10 | 1.57E-09 |
| GPRIN1 | 565.11 | 1.58 | 1.03E-54 | 4.53E-53 |
| POU2F2 | 54.79 | 1.58 | 2.47E-08 | 1.07E-07 |
| ST6GAL1 | 460.42 | 1.58 | 1.10E-38 | 2.77E-37 |
| MAGEA1 | 169.34 | 1.58 | 5.00E-18 | 4.54E-17 |
| KCNK2 | 5.90 | 1.58 | 1.92E-03 | 4.62E-03 |
| POTEC | 166.03 | 1.57 | 4.43E-17 | 3.76E-16 |
| ADGRA3 | 1642.25 | 1.57 | 1.06E-111 | 1.57E-109 |
| PRRG3 | 8.91 | 1.57 | 1.43E-03 | 3.51E-03 |
| CHD5 | 30.39 | 1.57 | 4.55E-06 | 1.57E-05 |
| NAV3 | 151.06 | 1.57 | 7.25E-17 | 6.10E-16 |
| GSG1 | 36.03 | 1.56 | 1.61E-06 | 5.83E-06 |
| CD248 | 5.93 | 1.56 | 1.98E-03 | 4.76E-03 |
| MMP7 | 571.24 | 1.55 | 2.02E-38 | 5.05E-37 |
| FJX1 | 1154.71 | 1.55 | 5.68E-80 | 4.81E-78 |
| RBM14-RBM4 | 230.99 | 1.55 | 3.31E-15 | 2.49E-14 |
| AADAT | 123.24 | 1.55 | 1.74E-14 | 1.25E-13 |
| ADAMTS12 | 1391.61 | 1.55 | 4.63E-63 | 2.76E-61 |
| EPHB3 | 10.96 | 1.55 | 7.39E-04 | 1.90E-03 |
| COL13A1 | 115.17 | 1.54 | 2.62E-14 | 1.84E-13 |
| CCBE1 | 133.34 | 1.54 | 5.40E-15 | 3.99E-14 |
| ZNF124 | 279.97 | 1.54 | 8.63E-28 | 1.34E-26 |
| ADORA1 | 103.62 | 1.54 | 1.07E-12 | 6.66E-12 |
| ZNF738 | 436.24 | 1.53 | 8.46E-37 | 1.94E-35 |
| ADM | 1247.28 | 1.52 | 6.03E-55 | 2.66E-53 |
| IL2RB | 17.56 | 1.52 | 1.48E-04 | 4.21E-04 |
| RHOBTB1 | 646.01 | 1.51 | 7.40E-47 | 2.55E-45 |
| FAM241B | 231.46 | 1.51 | 2.12E-22 | 2.44E-21 |
| GASK1B | 127.38 | 1.51 | 5.48E-14 | 3.76E-13 |
| VCX3A | 46.81 | 1.50 | 4.79E-07 | 1.83E-06 |
| ZBTB45P2 | 120.73 | 1.50 | 2.05E-10 | 1.07E-09 |
| DDX12P | 95.33 | 1.50 | 2.28E-11 | 1.29E-10 |
| JAG1 | 1558.37 | 1.50 | 6.60E-52 | 2.63E-50 |
| VWF | 19.03 | 1.50 | 1.55E-04 | 4.41E-04 |
| OR10G3 | 9.63 | 1.50 | 1.37E-03 | 3.39E-03 |
| ADAMTS7 | 27.10 | 1.50 | 2.85E-05 | 8.88E-05 |
| ANKRD1 | 4640.10 | 1.49 | 1.67E-97 | 1.96E-95 |
| LY6K | 151.31 | 1.49 | 1.27E-16 | 1.05E-15 |
| FHL2 | 1505.57 | 1.49 | 1.42E-92 | 1.50E-90 |
| KRT7 | 62.48 | 1.48 | 2.33E-08 | 1.01E-07 |
| BMF | 62.04 | 1.48 | 2.59E-08 | 1.12E-07 |
| INAVA | 60.40 | 1.48 | 1.89E-08 | 8.26E-08 |
| PHB1P6 | 3.21 | 1.48 | 2.39E-03 | 5.67E-03 |
| NGF | 130.26 | 1.48 | 6.63E-15 | 4.88E-14 |
| DAAM2 | 128.67 | 1.47 | 3.80E-14 | 2.64E-13 |
| TAS2R1 | 49.58 | 1.47 | 1.75E-07 | 6.99E-07 |
| HBEGF | 1185.82 | 1.46 | 1.95E-84 | 1.83E-82 |
| ABHD12B | 52.75 | 1.46 | 1.93E-07 | 7.68E-07 |
| UTP20 | 1247.96 | 1.46 | 3.07E-53 | 1.30E-51 |
| ZNF431 | 607.41 | 1.45 | 7.36E-47 | 2.54E-45 |
| CRACD | 836.62 | 1.45 | 1.84E-39 | 4.82E-38 |
| NAT8L | 383.06 | 1.44 | 4.01E-32 | 7.50E-31 |
| CCL20 | 16.30 | 1.44 | 4.33E-04 | 1.15E-03 |
| N4BP3 | 360.16 | 1.44 | 2.52E-31 | 4.53E-30 |
| FAM111B | 842.22 | 1.44 | 6.73E-54 | 2.89E-52 |
| TATDN2P2 | 57.65 | 1.43 | 6.76E-07 | 2.54E-06 |
| CCDC88C | 374.89 | 1.43 | 9.97E-24 | 1.23E-22 |
| MKRN3 | 234.81 | 1.43 | 2.60E-19 | 2.57E-18 |
| CDK6 | 1682.96 | 1.43 | 4.06E-53 | 1.70E-51 |
| NUAK1 | 1293.01 | 1.43 | 4.74E-63 | 2.82E-61 |
| IGFN1 | 7491.97 | 1.43 | 4.89E-81 | 4.28E-79 |
| ATP8A2 | 26.18 | 1.42 | 4.46E-05 | 1.36E-04 |
| CCDC150 | 251.93 | 1.42 | 2.07E-23 | 2.52E-22 |
| IRS1 | 901.86 | 1.42 | 8.39E-44 | 2.62E-42 |
| ESR2 | 8.99 | 1.42 | 1.65E-03 | 4.03E-03 |
| FREM2 | 78.00 | 1.42 | 1.87E-08 | 8.20E-08 |
| CARD9 | 33.28 | 1.42 | 1.15E-05 | 3.80E-05 |
| DLX2 | 172.69 | 1.41 | 2.37E-16 | 1.94E-15 |
| FOXO3B | 76.57 | 1.41 | 5.89E-09 | 2.72E-08 |
| ADARB1 | 528.24 | 1.41 | 5.98E-40 | 1.59E-38 |
| PRAG1 | 328.94 | 1.40 | 2.94E-26 | 4.18E-25 |
| ICAM4 | 6.92 | 1.40 | 2.55E-03 | 6.01E-03 |
| SNAI2 | 71.83 | 1.40 | 1.36E-08 | 6.05E-08 |
| CICP15 | 30.55 | 1.40 | 2.98E-05 | 9.28E-05 |
| XAGE1A | 169.74 | 1.39 | 2.31E-16 | 1.89E-15 |
| HIC2 | 205.96 | 1.39 | 3.21E-18 | 2.95E-17 |
| DOK7 | 21.87 | 1.39 | 1.75E-04 | 4.94E-04 |
| PM20D2 | 834.10 | 1.38 | 3.12E-54 | 1.35E-52 |
| HSPA8 | 25728.87 | 1.38 | 5.51E-163 | 1.57E-160 |
| CCNJ | 594.85 | 1.38 | 3.48E-38 | 8.54E-37 |
| ACE2 | 34.72 | 1.38 | 2.14E-05 | 6.80E-05 |
| PDCD11 | 2834.89 | 1.38 | 2.21E-68 | 1.56E-66 |
| DNER | 50.01 | 1.38 | 3.68E-06 | 1.28E-05 |
| GARIN1A | 26.93 | 1.38 | 8.13E-05 | 2.40E-04 |
| GOT2 | 1360.60 | 1.38 | 1.08E-74 | 8.61E-73 |
| SPDYE6 | 14.23 | 1.38 | 9.86E-04 | 2.49E-03 |
| ANGPTL2 | 4.31 | 1.38 | 3.17E-03 | 7.37E-03 |
| HNRNPA1L3 | 25.37 | 1.38 | 1.47E-04 | 4.18E-04 |
| TGFB2 | 1622.78 | 1.38 | 7.86E-78 | 6.46E-76 |
| PCDH7 | 454.09 | 1.37 | 4.25E-31 | 7.61E-30 |
| IGF1 | 688.21 | 1.37 | 2.31E-49 | 8.50E-48 |
| GOLGA8A | 299.94 | 1.37 | 1.28E-22 | 1.50E-21 |
| IL18BP | 240.73 | 1.37 | 1.46E-20 | 1.52E-19 |
| CXADRP3 | 85.67 | 1.37 | 1.02E-09 | 5.02E-09 |
| CRYBG2 | 51.91 | 1.36 | 4.88E-07 | 1.87E-06 |
| FHOD3 | 292.91 | 1.36 | 1.50E-23 | 1.84E-22 |
| DOK3 | 35.34 | 1.36 | 1.95E-05 | 6.23E-05 |
| CHD7 | 1504.67 | 1.36 | 1.04E-59 | 5.64E-58 |
| GRID1 | 3.00 | 1.35 | 3.19E-03 | 7.42E-03 |
| ARVCF | 636.16 | 1.35 | 4.41E-41 | 1.24E-39 |
| ITGAL | 14.53 | 1.35 | 6.64E-04 | 1.72E-03 |
| MYH2 | 8.34 | 1.35 | 3.01E-03 | 7.02E-03 |
| INCENP | 2072.75 | 1.35 | 3.33E-86 | 3.27E-84 |
| ZNF729 | 10.12 | 1.35 | 1.74E-03 | 4.24E-03 |
| ASB2 | 2.86 | 1.35 | 3.28E-03 | 7.60E-03 |
| SMPD3 | 29.89 | 1.34 | 7.79E-05 | 2.31E-04 |
| RUBCNL | 65.35 | 1.34 | 5.33E-08 | 2.25E-07 |
| NT5DC2 | 484.09 | 1.34 | 1.13E-35 | 2.47E-34 |
| HNRNPAB | 7046.31 | 1.34 | 1.68E-132 | 3.38E-130 |
| EFHD1 | 4.15 | 1.34 | 3.51E-03 | 8.09E-03 |
| PCDHGB3 | 26.71 | 1.34 | 1.68E-04 | 4.75E-04 |
| UBL5 | 1396.68 | 1.34 | 3.14E-63 | 1.88E-61 |
| GALNT16 | 30.09 | 1.34 | 1.37E-04 | 3.91E-04 |
| EPHA2 | 1658.70 | 1.33 | 2.29E-85 | 2.20E-83 |
| PARD6G | 283.41 | 1.33 | 3.27E-21 | 3.55E-20 |
| EVA1A | 331.48 | 1.33 | 3.93E-22 | 4.48E-21 |
| FGF9 | 24.62 | 1.33 | 1.27E-04 | 3.65E-04 |
| IL31RA | 66.96 | 1.32 | 8.77E-08 | 3.61E-07 |
| MYLK3 | 325.54 | 1.32 | 2.11E-22 | 2.44E-21 |
| SOX15 | 42.38 | 1.32 | 5.11E-06 | 1.75E-05 |
| GLB1L3 | 271.17 | 1.32 | 8.70E-18 | 7.72E-17 |
| CALD1 | 7320.68 | 1.32 | 1.61E-121 | 2.84E-119 |
| RPL13P12 | 108.80 | 1.32 | 1.34E-09 | 6.52E-09 |
| SLC6A10P | 3.14 | 1.32 | 3.44E-03 | 7.95E-03 |
| RELT | 430.73 | 1.32 | 2.13E-31 | 3.86E-30 |
| PPFIA1 | 302.14 | 1.31 | 4.18E-06 | 1.45E-05 |
| ABCC5 | 1242.07 | 1.31 | 1.43E-48 | 5.18E-47 |
| SLIT2 | 579.76 | 1.31 | 1.40E-32 | 2.68E-31 |
| FAM171A1 | 888.91 | 1.31 | 4.06E-53 | 1.70E-51 |
| CADM3 | 4.27 | 1.31 | 3.77E-03 | 8.65E-03 |
| SLC43A3 | 1540.14 | 1.31 | 1.53E-76 | 1.24E-74 |
| WTIP | 637.64 | 1.30 | 8.05E-41 | 2.24E-39 |
| CENPV | 786.71 | 1.30 | 3.31E-45 | 1.08E-43 |
| SH3BGRL2 | 289.50 | 1.30 | 1.39E-21 | 1.53E-20 |
| BRME1 | 56.43 | 1.29 | 3.14E-06 | 1.10E-05 |
| MCRIP2P1 | 30.94 | 1.29 | 7.95E-05 | 2.35E-04 |
| S100A2 | 150.49 | 1.29 | 1.69E-11 | 9.67E-11 |
| TENM2 | 277.26 | 1.29 | 7.78E-18 | 6.95E-17 |
| POLR3K | 698.09 | 1.29 | 1.56E-42 | 4.63E-41 |
| NMNAT2 | 63.05 | 1.29 | 4.95E-07 | 1.89E-06 |
| ZNF473 | 485.39 | 1.29 | 1.23E-26 | 1.79E-25 |
| SULT1A4 | 42.99 | 1.29 | 1.68E-05 | 5.41E-05 |
| ZNF708 | 198.84 | 1.28 | 1.36E-15 | 1.05E-14 |
| NFASC | 48.71 | 1.28 | 7.51E-06 | 2.53E-05 |
| EPHB2 | 354.66 | 1.28 | 2.97E-24 | 3.77E-23 |
| SIM2 | 59.54 | 1.28 | 2.28E-06 | 8.13E-06 |
| IP6K2 | 730.49 | 1.27 | 3.42E-44 | 1.08E-42 |
| SGK1 | 178.17 | 1.27 | 4.10E-15 | 3.06E-14 |
| FAM86C1P | 26.97 | 1.27 | 1.61E-04 | 4.57E-04 |
| PPME1 | 3287.06 | 1.27 | 2.47E-109 | 3.58E-107 |
| RPS3AP5 | 38.47 | 1.27 | 6.16E-05 | 1.85E-04 |
| GLI2 | 325.14 | 1.27 | 2.33E-21 | 2.55E-20 |
| ANKRD63 | 124.65 | 1.26 | 1.61E-09 | 7.76E-09 |
| KLHL25 | 141.86 | 1.26 | 1.38E-11 | 7.93E-11 |
| POLE | 1630.06 | 1.26 | 6.57E-57 | 3.20E-55 |
| CITED2 | 1275.69 | 1.26 | 8.66E-60 | 4.76E-58 |
| TMPRSS9 | 56.84 | 1.26 | 2.12E-05 | 6.77E-05 |
| KLF5 | 812.33 | 1.25 | 8.92E-40 | 2.35E-38 |
| AMOTL1 | 1476.85 | 1.25 | 4.16E-44 | 1.31E-42 |
| QRICH2 | 31.18 | 1.25 | 7.61E-05 | 2.25E-04 |
| IFNLR1 | 102.30 | 1.25 | 9.69E-10 | 4.79E-09 |
| IGF1R | 1269.06 | 1.25 | 3.65E-51 | 1.42E-49 |
| SLC44A3-AS1 | 522.56 | 1.25 | 1.79E-29 | 2.98E-28 |
| SMARCB1 | 136.21 | 1.25 | 5.03E-08 | 2.12E-07 |
| SPINDOC | 1332.13 | 1.25 | 3.23E-57 | 1.60E-55 |
| FUS | 8293.70 | 1.25 | 3.32E-74 | 2.61E-72 |
| ZNF492 | 282.89 | 1.25 | 1.16E-20 | 1.21E-19 |
| POLR2F | 626.82 | 1.25 | 5.17E-37 | 1.20E-35 |
| PRKX | 753.95 | 1.24 | 1.93E-34 | 4.02E-33 |
| NANOS1 | 173.51 | 1.24 | 1.22E-12 | 7.60E-12 |
| COPS9 | 473.53 | 1.24 | 9.87E-30 | 1.66E-28 |
| RCBTB2 | 75.70 | 1.24 | 6.91E-08 | 2.87E-07 |
| OLIG2 | 3.99 | 1.24 | 4.49E-03 | 1.02E-02 |
| TCF3 | 4540.06 | 1.23 | 8.94E-132 | 1.78E-129 |
| SMG1P6 | 18.45 | 1.23 | 1.01E-03 | 2.55E-03 |
| BDNF | 102.15 | 1.23 | 2.72E-09 | 1.29E-08 |
| PLXNB1 | 551.79 | 1.23 | 8.80E-32 | 1.62E-30 |
| ZBTB39 | 410.42 | 1.23 | 1.03E-18 | 9.79E-18 |
| GPR19 | 35.94 | 1.23 | 5.74E-05 | 1.73E-04 |
| HNRNPA0 | 6041.20 | 1.23 | 8.01E-107 | 1.10E-104 |
| BEND3 | 398.16 | 1.22 | 3.51E-21 | 3.80E-20 |
| SAPCD2 | 1849.26 | 1.22 | 5.59E-77 | 4.55E-75 |
| DNLZ | 215.47 | 1.22 | 3.51E-13 | 2.26E-12 |
| DOHH | 866.68 | 1.22 | 5.73E-42 | 1.66E-40 |
| FILIP1L | 325.78 | 1.22 | 1.31E-21 | 1.45E-20 |
| LHX4 | 147.15 | 1.22 | 1.02E-11 | 5.94E-11 |
| EID3 | 90.43 | 1.22 | 1.80E-08 | 7.89E-08 |
| ASNS | 1894.86 | 1.22 | 6.02E-73 | 4.62E-71 |
| ZBED4 | 1043.69 | 1.22 | 1.11E-43 | 3.43E-42 |
| PSD3 | 208.97 | 1.22 | 1.67E-13 | 1.11E-12 |
| CCN2 | 2386.71 | 1.22 | 6.14E-62 | 3.55E-60 |
| SERPINB8 | 246.92 | 1.22 | 9.89E-16 | 7.71E-15 |
| PRR22 | 92.81 | 1.22 | 5.42E-08 | 2.28E-07 |
| HAS3 | 56.96 | 1.22 | 3.58E-06 | 1.25E-05 |
| TLR4 | 718.82 | 1.22 | 1.17E-32 | 2.24E-31 |
| CD274 | 1517.08 | 1.22 | 7.67E-65 | 4.74E-63 |
| PDE4A | 18.28 | 1.21 | 8.50E-04 | 2.17E-03 |
| ONECUT2 | 297.40 | 1.21 | 1.87E-17 | 1.63E-16 |
| NOP16 | 1230.12 | 1.21 | 1.48E-52 | 6.12E-51 |
| DGCR8 | 819.62 | 1.21 | 4.83E-38 | 1.18E-36 |
| GASK1A | 2356.48 | 1.21 | 4.36E-40 | 1.18E-38 |
| HCFC1 | 4874.79 | 1.21 | 7.26E-50 | 2.74E-48 |
| ADGRG2 | 19.42 | 1.21 | 6.94E-04 | 1.79E-03 |
| CCNE2 | 251.74 | 1.21 | 8.62E-17 | 7.22E-16 |
| CSTF2 | 784.82 | 1.21 | 2.59E-39 | 6.70E-38 |
| CSRP2 | 271.71 | 1.20 | 1.45E-17 | 1.27E-16 |
| TNFRSF10D | 99.41 | 1.20 | 2.56E-08 | 1.11E-07 |
| SAFB2 | 1753.27 | 1.20 | 5.90E-54 | 2.54E-52 |
| ARID3B | 96.80 | 1.20 | 1.24E-08 | 5.54E-08 |
| F3 | 461.96 | 1.20 | 5.62E-22 | 6.35E-21 |
| FRAS1 | 488.84 | 1.20 | 1.92E-19 | 1.91E-18 |
| C14orf132 | 216.00 | 1.20 | 4.18E-16 | 3.34E-15 |
| SYNGR1 | 26.49 | 1.19 | 3.71E-04 | 1.00E-03 |
| TCOF1 | 8718.28 | 1.19 | 4.14E-124 | 7.44E-122 |
| ITGBL1 | 5.61 | 1.19 | 5.24E-03 | 1.17E-02 |
| ZNF782 | 113.52 | 1.19 | 4.69E-09 | 2.18E-08 |
| C2CD3 | 1997.61 | 1.19 | 2.43E-57 | 1.21E-55 |
| FOXD4L1 | 5.60 | 1.19 | 5.24E-03 | 1.17E-02 |
| FGF5 | 7.04 | 1.19 | 4.76E-03 | 1.07E-02 |
| ADGRV1 | 187.03 | 1.19 | 2.54E-13 | 1.66E-12 |
| POLR2J2 | 68.51 | 1.19 | 2.33E-06 | 8.30E-06 |
| CTPS1 | 3762.70 | 1.19 | 9.43E-90 | 9.49E-88 |
| PIF1 | 313.35 | 1.19 | 3.42E-20 | 3.51E-19 |
| POLR2A | 30.52 | 1.18 | 3.27E-04 | 8.87E-04 |
| DAW1 | 13.87 | 1.18 | 1.89E-03 | 4.57E-03 |
| TXK | 35.98 | 1.18 | 6.68E-05 | 2.00E-04 |
| TMEM131L | 447.32 | 1.18 | 6.26E-24 | 7.78E-23 |
| ROR1 | 367.94 | 1.18 | 1.66E-22 | 1.94E-21 |
| PRICKLE1 | 234.90 | 1.18 | 1.82E-15 | 1.40E-14 |
| C4BPB | 163.68 | 1.18 | 2.76E-12 | 1.67E-11 |
| RRM2 | 3368.97 | 1.18 | 3.64E-96 | 4.20E-94 |
| CICP8 | 3.84 | 1.18 | 5.32E-03 | 1.19E-02 |
| NUTM2G | 39.22 | 1.18 | 5.39E-05 | 1.63E-04 |
| TEN1-CDK3 | 50.50 | 1.18 | 2.10E-05 | 6.70E-05 |
| COL12A1 | 3251.03 | 1.17 | 2.67E-13 | 1.74E-12 |
| CBX2 | 1142.76 | 1.17 | 4.00E-52 | 1.61E-50 |
| FBLIM1 | 1057.67 | 1.17 | 1.24E-46 | 4.25E-45 |
| DOCK4 | 179.01 | 1.17 | 1.51E-12 | 9.35E-12 |
| PSMC3IP | 524.57 | 1.17 | 1.64E-25 | 2.23E-24 |
| SEMA4D | 49.58 | 1.17 | 4.01E-05 | 1.23E-04 |
| SRRT | 2755.86 | 1.16 | 2.69E-78 | 2.25E-76 |
| NT5C1A | 23.48 | 1.16 | 4.29E-04 | 1.14E-03 |
| ZNF480 | 290.63 | 1.16 | 2.89E-17 | 2.48E-16 |
| NOP56 | 3044.58 | 1.16 | 6.42E-100 | 7.85E-98 |
| MEX3D | 2692.52 | 1.16 | 5.69E-90 | 5.77E-88 |
| EN2 | 13.73 | 1.16 | 2.17E-03 | 5.18E-03 |
| NLRP3P1 | 5.61 | 1.16 | 5.89E-03 | 1.30E-02 |
| NKX2-1 | 11.64 | 1.15 | 2.66E-03 | 6.26E-03 |
| ZNF141 | 404.69 | 1.15 | 3.96E-21 | 4.28E-20 |
| GPRC5D | 16.34 | 1.15 | 1.32E-03 | 3.28E-03 |
| SACS | 1282.08 | 1.15 | 9.75E-45 | 3.13E-43 |
| LFNG | 64.55 | 1.15 | 2.52E-06 | 8.95E-06 |
| ZWINT | 1392.16 | 1.15 | 1.36E-60 | 7.62E-59 |
| CYBA | 18.42 | 1.15 | 1.21E-03 | 3.03E-03 |
| PTPRO | 34.22 | 1.15 | 2.02E-04 | 5.62E-04 |
| LMNB1 | 4616.20 | 1.15 | 9.69E-86 | 9.41E-84 |
| SBK1 | 36.76 | 1.15 | 1.32E-04 | 3.80E-04 |
| LMNB2 | 10450.55 | 1.15 | 2.98E-141 | 6.38E-139 |
| ITGB3 | 475.11 | 1.14 | 2.11E-21 | 2.32E-20 |
| DAAM1 | 150.99 | 1.14 | 2.12E-10 | 1.11E-09 |
| KLF2P4 | 81.50 | 1.14 | 6.37E-07 | 2.40E-06 |
| MLLT6 | 519.34 | 1.14 | 6.25E-05 | 1.87E-04 |
| HHIP | 27.49 | 1.14 | 3.09E-04 | 8.42E-04 |
| NRGN | 303.63 | 1.14 | 4.82E-18 | 4.38E-17 |
| AGAP9 | 62.91 | 1.14 | 9.67E-06 | 3.21E-05 |
| RAB15 | 501.04 | 1.14 | 3.72E-28 | 5.87E-27 |
| POLR1A | 1738.84 | 1.14 | 3.22E-52 | 1.30E-50 |
| CALML4 | 33.57 | 1.14 | 1.90E-04 | 5.32E-04 |
| YJEFN3 | 177.77 | 1.14 | 9.00E-11 | 4.85E-10 |
| MMP24 | 29.75 | 1.14 | 6.38E-04 | 1.66E-03 |
| ZNF714 | 1064.10 | 1.14 | 1.06E-31 | 1.94E-30 |
| TAF15 | 1889.61 | 1.13 | 2.27E-34 | 4.70E-33 |
| TMSB15B | 53.17 | 1.13 | 3.71E-05 | 1.14E-04 |
| MAML1 | 1201.64 | 1.13 | 2.01E-33 | 3.97E-32 |
| POTEE | 110.58 | 1.13 | 2.91E-07 | 1.14E-06 |
| SPOCD1 | 211.29 | 1.13 | 4.48E-14 | 3.09E-13 |
| CHST3 | 808.39 | 1.13 | 2.81E-37 | 6.64E-36 |
| RBM19 | 959.62 | 1.13 | 5.06E-43 | 1.54E-41 |
| BCOR | 1009.30 | 1.13 | 9.20E-41 | 2.54E-39 |
| MYH10 | 3945.86 | 1.13 | 2.14E-58 | 1.12E-56 |
| TGIF2-RAB5IF | 2.71 | 1.13 | 5.60E-03 | 1.24E-02 |
| ZNF728 | 39.44 | 1.13 | 3.25E-04 | 8.82E-04 |
| FSD2 | 52.38 | 1.13 | 2.14E-05 | 6.82E-05 |
| SYN3 | 1551.89 | 1.13 | 8.46E-46 | 2.86E-44 |
| SLC16A12 | 9.67 | 1.13 | 3.88E-03 | 8.87E-03 |
| BRI3BP | 1013.01 | 1.12 | 1.26E-45 | 4.22E-44 |
| TBC1D16 | 1899.21 | 1.12 | 5.62E-52 | 2.25E-50 |
| EFNB2 | 89.17 | 1.12 | 1.05E-06 | 3.88E-06 |
| POLR1E | 733.04 | 1.12 | 6.02E-36 | 1.32E-34 |
| SEPTIN14P19 | 11.04 | 1.12 | 3.55E-03 | 8.18E-03 |
| CXCL8 | 39.35 | 1.12 | 2.92E-04 | 7.97E-04 |
| TDRD9 | 2.54 | 1.12 | 5.87E-03 | 1.30E-02 |
| CHEK1 | 1033.48 | 1.12 | 2.54E-44 | 8.04E-43 |
| SEPTIN3 | 236.64 | 1.11 | 3.25E-14 | 2.27E-13 |
| GATA2 | 497.13 | 1.11 | 1.24E-24 | 1.61E-23 |
| ZNF112 | 398.81 | 1.11 | 2.09E-22 | 2.42E-21 |
| ZNF195 | 829.77 | 1.11 | 3.09E-37 | 7.28E-36 |
| ANKRD13B | 495.46 | 1.11 | 6.83E-25 | 9.03E-24 |
| CCDC77 | 539.87 | 1.11 | 9.87E-29 | 1.60E-27 |
| KRT10 | 358.10 | 1.11 | 3.94E-20 | 4.03E-19 |
| ATAD3B | 2059.03 | 1.11 | 1.15E-41 | 3.28E-40 |
| B4GALNT4 | 146.74 | 1.11 | 6.46E-10 | 3.24E-09 |
| CNTF | 37.21 | 1.11 | 2.71E-04 | 7.43E-04 |
| ZNF232 | 446.82 | 1.11 | 1.72E-23 | 2.10E-22 |
| KIF1C | 3095.00 | 1.11 | 7.36E-56 | 3.42E-54 |
| CCDC134 | 338.56 | 1.11 | 7.71E-20 | 7.77E-19 |
| LAT | 68.23 | 1.11 | 1.62E-06 | 5.85E-06 |
| BYSL | 826.73 | 1.11 | 4.23E-36 | 9.40E-35 |
| AMIGO2 | 359.70 | 1.11 | 1.96E-20 | 2.03E-19 |
| IL7R | 1224.71 | 1.11 | 3.65E-38 | 8.94E-37 |
| ANKRD30B | 72.66 | 1.11 | 1.55E-06 | 5.62E-06 |
| C3orf52 | 208.97 | 1.11 | 1.18E-12 | 7.32E-12 |
| LRFN4 | 219.36 | 1.11 | 9.37E-13 | 5.88E-12 |
| PNN | 2241.56 | 1.11 | 5.77E-56 | 2.69E-54 |
| ZNF696 | 531.53 | 1.11 | 2.39E-24 | 3.05E-23 |
| CASP2 | 1133.77 | 1.10 | 7.34E-43 | 2.22E-41 |
| MDN1 | 2292.37 | 1.10 | 7.23E-32 | 1.34E-30 |
| MARCHF1 | 59.89 | 1.10 | 1.96E-05 | 6.28E-05 |
| PHF14 | 706.15 | 1.10 | 2.18E-32 | 4.15E-31 |
| NME3 | 50.86 | 1.10 | 4.65E-05 | 1.41E-04 |
| ATP5ME | 517.58 | 1.10 | 1.08E-20 | 1.13E-19 |
| ZNF285BP | 8.38 | 1.10 | 5.26E-03 | 1.17E-02 |
| ODC1 | 3614.11 | 1.10 | 9.67E-91 | 9.86E-89 |
| SKP2 | 2890.88 | 1.10 | 1.89E-84 | 1.79E-82 |
| TEAD4 | 395.26 | 1.10 | 4.61E-22 | 5.25E-21 |
| UPF1 | 2631.95 | 1.10 | 6.74E-67 | 4.50E-65 |
| KIF18B | 1346.56 | 1.10 | 8.96E-52 | 3.56E-50 |
| ZBTB40 | 1602.22 | 1.09 | 1.48E-38 | 3.72E-37 |
| GAL3ST3 | 8.85 | 1.09 | 4.94E-03 | 1.11E-02 |
| SLC28A3 | 16.01 | 1.09 | 2.04E-03 | 4.88E-03 |
| SRCAP | 1398.70 | 1.09 | 1.68E-08 | 7.41E-08 |
| PODXL | 1618.86 | 1.09 | 4.21E-48 | 1.51E-46 |
| PLAGL2 | 1098.15 | 1.09 | 1.46E-44 | 4.67E-43 |
| PFAS | 2553.76 | 1.09 | 1.95E-66 | 1.29E-64 |
| ID2 | 361.08 | 1.09 | 3.18E-17 | 2.72E-16 |
| DYSF | 188.59 | 1.08 | 2.32E-11 | 1.31E-10 |
| MCM10 | 954.34 | 1.08 | 2.71E-32 | 5.12E-31 |
| BRIP1 | 1045.63 | 1.08 | 1.62E-40 | 4.44E-39 |
| CLCF1 | 117.27 | 1.08 | 8.71E-09 | 3.96E-08 |
| MCM7 | 7638.14 | 1.08 | 5.03E-109 | 7.17E-107 |
| MCM4 | 5884.92 | 1.08 | 2.20E-105 | 2.95E-103 |
| SLFN13 | 39.66 | 1.08 | 9.59E-05 | 2.80E-04 |
| ZNF695 | 172.96 | 1.08 | 3.48E-11 | 1.94E-10 |
| PRDM15 | 347.53 | 1.08 | 7.72E-17 | 6.48E-16 |
| BCL7A | 505.11 | 1.08 | 2.38E-24 | 3.05E-23 |
| RGL4 | 90.04 | 1.08 | 4.42E-07 | 1.70E-06 |
| SDR42E1 | 162.09 | 1.07 | 2.48E-10 | 1.29E-09 |
| ILF3 | 9398.87 | 1.07 | 1.35E-106 | 1.84E-104 |
| ZNF519 | 375.96 | 1.07 | 1.17E-19 | 1.17E-18 |
| SLC5A6 | 1513.39 | 1.07 | 5.09E-55 | 2.26E-53 |
| FGFBP1 | 2062.14 | 1.07 | 3.35E-67 | 2.26E-65 |
| C17orf107 | 17.02 | 1.07 | 1.96E-03 | 4.71E-03 |
| ARHGEF26 | 167.37 | 1.07 | 4.84E-07 | 1.85E-06 |
| MCM2 | 5865.04 | 1.07 | 3.91E-98 | 4.65E-96 |
| C2orf50 | 7.30 | 1.07 | 6.89E-03 | 1.50E-02 |
| FRMD8 | 890.64 | 1.07 | 2.71E-35 | 5.83E-34 |
| RPL9 | 875.52 | 1.07 | 1.09E-38 | 2.76E-37 |
| SPRY4 | 193.41 | 1.07 | 1.32E-10 | 7.01E-10 |
| UNG | 879.28 | 1.07 | 4.40E-37 | 1.03E-35 |
| PDE3B | 183.55 | 1.07 | 2.49E-11 | 1.41E-10 |
| C19orf48P | 778.77 | 1.07 | 7.62E-29 | 1.24E-27 |
| LTO1 | 270.94 | 1.07 | 3.55E-15 | 2.66E-14 |
| BAZ1A | 1734.87 | 1.06 | 3.19E-55 | 1.44E-53 |
| LPAR1 | 54.63 | 1.06 | 4.20E-05 | 1.29E-04 |
| CALB1 | 404.58 | 1.06 | 1.71E-11 | 9.77E-11 |
| AKAP12 | 3346.84 | 1.06 | 2.94E-54 | 1.28E-52 |
| GRIN2B | 2.53 | 1.06 | 6.83E-03 | 1.49E-02 |
| CD33 | 92.77 | 1.06 | 4.53E-07 | 1.74E-06 |
| CENPO | 1040.02 | 1.06 | 4.65E-38 | 1.13E-36 |
| SGPP2 | 11.15 | 1.06 | 4.44E-03 | 1.00E-02 |
| PLCXD2 | 163.19 | 1.06 | 9.96E-09 | 4.50E-08 |
| RGS7 | 44.21 | 1.06 | 9.40E-05 | 2.75E-04 |
| FAT1 | 2418.48 | 1.06 | 1.34E-09 | 6.51E-09 |
| VIM | 103.22 | 1.06 | 2.95E-07 | 1.16E-06 |
| AMOT | 529.57 | 1.05 | 3.10E-21 | 3.37E-20 |
| PUS7 | 507.32 | 1.05 | 4.61E-22 | 5.24E-21 |
| FN1 | 10785.07 | 1.05 | 1.08E-12 | 6.75E-12 |
| RGS12 | 349.73 | 1.05 | 1.49E-18 | 1.40E-17 |
| TXNRD2 | 1007.34 | 1.05 | 1.40E-35 | 3.03E-34 |
| SMARCC1 | 4527.75 | 1.05 | 2.34E-52 | 9.53E-51 |
| ZNF66 | 289.63 | 1.05 | 3.08E-11 | 1.72E-10 |
| RDH13 | 247.40 | 1.05 | 2.30E-09 | 1.10E-08 |
| CENPX | 728.22 | 1.05 | 3.16E-21 | 3.43E-20 |
| CTXN1 | 641.20 | 1.05 | 2.35E-28 | 3.74E-27 |
| RTKN2 | 343.89 | 1.04 | 8.60E-18 | 7.65E-17 |
| SAMD5 | 89.96 | 1.04 | 2.17E-06 | 7.74E-06 |
| ZSCAN5A | 90.04 | 1.04 | 4.70E-07 | 1.80E-06 |
| SERPINB7 | 13.90 | 1.04 | 3.28E-03 | 7.60E-03 |
| TRIM36 | 291.20 | 1.04 | 1.31E-14 | 9.44E-14 |
| CYP4F8 | 5.75 | 1.04 | 8.34E-03 | 1.79E-02 |
| NAV2 | 1081.70 | 1.04 | 2.41E-24 | 3.08E-23 |
| TEAD2 | 853.22 | 1.04 | 6.06E-34 | 1.23E-32 |
| CSNK1E | 2820.37 | 1.04 | 6.08E-72 | 4.54E-70 |
| ETS1 | 2304.85 | 1.04 | 5.51E-53 | 2.30E-51 |
| PCDHGA5 | 40.75 | 1.04 | 2.18E-04 | 6.06E-04 |
| HHLA2 | 8.40 | 1.04 | 7.04E-03 | 1.53E-02 |
| ZNF786 | 186.56 | 1.03 | 2.80E-11 | 1.57E-10 |
| MMP12 | 5.31 | 1.03 | 8.81E-03 | 1.88E-02 |
| FZD3 | 363.02 | 1.03 | 1.63E-18 | 1.52E-17 |
| JPT1 | 1784.83 | 1.03 | 5.21E-54 | 2.25E-52 |
| OTUD3 | 678.45 | 1.03 | 3.01E-18 | 2.77E-17 |
| SCRIB | 3338.12 | 1.03 | 6.46E-66 | 4.18E-64 |
| TRPM2 | 195.44 | 1.03 | 2.47E-11 | 1.39E-10 |
| LRRC37A | 138.44 | 1.03 | 1.30E-06 | 4.76E-06 |
| NBPF20 | 415.92 | 1.03 | 7.84E-19 | 7.51E-18 |
| ENPP7P12 | 3.50 | 1.03 | 8.21E-03 | 1.76E-02 |
| SLC45A3 | 341.67 | 1.03 | 1.95E-15 | 1.50E-14 |
| EXOSC4 | 1516.48 | 1.03 | 3.04E-36 | 6.84E-35 |
| ZNF778 | 600.39 | 1.02 | 1.51E-25 | 2.05E-24 |
| RRS1 | 1100.66 | 1.02 | 1.26E-40 | 3.46E-39 |
| HMGB1P5 | 1147.81 | 1.02 | 9.33E-38 | 2.25E-36 |
| CHRNA10 | 13.26 | 1.02 | 4.13E-03 | 9.39E-03 |
| PPP2R2B | 38.25 | 1.02 | 2.59E-04 | 7.12E-04 |
| SFPQ | 9926.40 | 1.02 | 7.38E-113 | 1.11E-110 |
| EXOSC2 | 1980.54 | 1.02 | 2.49E-57 | 1.24E-55 |
| TIMM10 | 509.50 | 1.02 | 4.54E-18 | 4.13E-17 |
| CLSTN2 | 190.56 | 1.02 | 6.92E-10 | 3.46E-09 |
| GPR161 | 253.13 | 1.02 | 1.23E-13 | 8.25E-13 |
| RORB | 90.26 | 1.02 | 1.52E-06 | 5.54E-06 |
| ARID1A | 3536.22 | 1.02 | 1.09E-12 | 6.81E-12 |
| CEP170P1 | 5.14 | 1.02 | 9.33E-03 | 1.98E-02 |
| CAMSAP1 | 2090.66 | 1.02 | 2.65E-38 | 6.55E-37 |
| FLNC | 7173.02 | 1.02 | 3.91E-48 | 1.41E-46 |
| MYBPH | 2.39 | 1.01 | 7.82E-03 | 1.69E-02 |
| CYB5R2 | 11.63 | 1.01 | 5.29E-03 | 1.18E-02 |
| KLHL23 | 917.26 | 1.01 | 4.82E-31 | 8.60E-30 |
| ZFP69B | 102.34 | 1.01 | 1.10E-06 | 4.03E-06 |
| DCAF4L1 | 21.46 | 1.01 | 1.62E-03 | 3.96E-03 |
| PCF11 | 706.81 | 1.01 | 1.06E-28 | 1.72E-27 |
| BZW1P2 | 372.11 | 1.01 | 4.82E-12 | 2.86E-11 |
| FANCB | 384.88 | 1.01 | 3.48E-12 | 2.09E-11 |
| NPB | 21.32 | 1.01 | 2.27E-03 | 5.41E-03 |
| HNRNPM | 7431.52 | 1.01 | 3.54E-69 | 2.54E-67 |
| GRWD1 | 1465.60 | 1.01 | 6.19E-43 | 1.88E-41 |
| MAP3K1 | 574.16 | 1.01 | 1.80E-24 | 2.33E-23 |
| CREBBP | 1691.13 | 1.01 | 1.75E-29 | 2.92E-28 |
| FOSL1 | 986.37 | 1.01 | 4.66E-35 | 9.93E-34 |
| VCX2 | 39.07 | 1.01 | 4.20E-04 | 1.12E-03 |
| U2AF1 | 1801.26 | 1.01 | 2.38E-47 | 8.40E-46 |
| ZNF892 | 225.13 | 1.01 | 1.52E-09 | 7.38E-09 |
| SMG1P7 | 26.81 | 1.01 | 9.45E-04 | 2.40E-03 |
| FGF12 | 34.12 | 1.01 | 4.36E-04 | 1.16E-03 |
| SLC7A2 | 65.34 | 1.00 | 1.76E-05 | 5.67E-05 |
| LMO7 | 1359.79 | 1.00 | 1.90E-33 | 3.75E-32 |
| XRCC2 | 784.85 | 1.00 | 6.42E-28 | 1.00E-26 |
| PTPRS | 437.75 | 1.00 | 4.80E-19 | 4.66E-18 |
| URB2 | 504.60 | 1.00 | 1.64E-18 | 1.54E-17 |
| BNIP3P17 | 40.66 | 1.00 | 3.26E-04 | 8.84E-04 |
| ZNF589 | 183.48 | 1.00 | 1.82E-10 | 9.55E-10 |
| SNURF | 32.50 | 1.00 | 1.01E-03 | 2.54E-03 |
| PRKRIP1 | 874.51 | 1.00 | 1.36E-28 | 2.19E-27 |
| CHODL | 28.92 | -1.00 | 7.87E-04 | 2.02E-03 |
| FAUP4 | 37.61 | -1.00 | 4.52E-04 | 1.20E-03 |
| NQO2 | 1615.46 | -1.00 | 3.63E-46 | 1.23E-44 |
| RHOT1 | 649.25 | -1.00 | 3.21E-27 | 4.82E-26 |
| CCDC103 | 65.02 | -1.00 | 2.13E-05 | 6.79E-05 |
| GOLGA2 | 3569.94 | -1.00 | 7.81E-71 | 5.76E-69 |
| DNHD1 | 375.99 | -1.00 | 1.36E-16 | 1.13E-15 |
| OPTN | 2292.17 | -1.00 | 2.02E-45 | 6.72E-44 |
| HSPG2 | 3485.66 | -1.00 | 1.46E-11 | 8.41E-11 |
| PIR | 874.63 | -1.00 | 7.52E-31 | 1.33E-29 |
| SPTBN4 | 33.15 | -1.00 | 5.57E-04 | 1.46E-03 |
| PLCB1 | 309.28 | -1.00 | 6.19E-15 | 4.56E-14 |
| GTPBP2 | 1757.14 | -1.00 | 6.45E-55 | 2.84E-53 |
| AQP3 | 78.29 | -1.00 | 3.60E-06 | 1.26E-05 |
| MVD | 2919.79 | -1.00 | 7.66E-37 | 1.76E-35 |
| STX1B | 45.60 | -1.01 | 1.23E-04 | 3.56E-04 |
| TRAF1 | 301.62 | -1.01 | 5.77E-14 | 3.95E-13 |
| SGTB | 481.79 | -1.01 | 2.37E-20 | 2.44E-19 |
| CSF2RB | 13.54 | -1.01 | 5.32E-03 | 1.19E-02 |
| SNCA | 392.78 | -1.01 | 1.27E-16 | 1.05E-15 |
| DENND3 | 1349.00 | -1.01 | 3.16E-46 | 1.08E-44 |
| SYNPO | 1982.64 | -1.01 | 1.01E-49 | 3.78E-48 |
| NHERF1 | 2913.96 | -1.01 | 1.19E-55 | 5.42E-54 |
| PRSS8 | 45.97 | -1.01 | 1.52E-04 | 4.32E-04 |
| PITPNM2 | 307.86 | -1.01 | 1.08E-14 | 7.79E-14 |
| CATIP | 4.75 | -1.01 | 9.56E-03 | 2.02E-02 |
| COQ8B | 868.70 | -1.01 | 2.58E-33 | 5.03E-32 |
| H2BC21 | 45.85 | -1.01 | 1.50E-04 | 4.25E-04 |
| MBOAT7 | 995.25 | -1.01 | 2.22E-23 | 2.70E-22 |
| ELF3 | 382.78 | -1.01 | 6.17E-15 | 4.55E-14 |
| TPCN1 | 2553.47 | -1.01 | 8.61E-48 | 3.07E-46 |
| PATJ | 735.99 | -1.01 | 1.60E-29 | 2.69E-28 |
| OSTM1 | 1694.88 | -1.01 | 4.74E-44 | 1.48E-42 |
| LRRC23 | 89.87 | -1.01 | 8.64E-06 | 2.88E-05 |
| CCDC113 | 319.30 | -1.01 | 9.40E-16 | 7.35E-15 |
| PSMB3 | 1433.80 | -1.01 | 4.42E-31 | 7.90E-30 |
| VEGFB | 1726.77 | -1.01 | 1.36E-48 | 4.93E-47 |
| MVB12A | 958.70 | -1.01 | 2.98E-27 | 4.49E-26 |
| PROM1 | 33.18 | -1.01 | 6.88E-04 | 1.78E-03 |
| CHAC1 | 94.07 | -1.01 | 1.34E-06 | 4.89E-06 |
| LPCAT1 | 1887.82 | -1.02 | 4.77E-44 | 1.49E-42 |
| MDH1 | 4134.10 | -1.02 | 5.30E-62 | 3.08E-60 |
| DGKA | 183.88 | -1.02 | 5.05E-10 | 2.56E-09 |
| IFITM2 | 995.67 | -1.02 | 1.70E-33 | 3.36E-32 |
| FAM76A | 214.86 | -1.02 | 1.97E-11 | 1.12E-10 |
| LRP10 | 1566.04 | -1.02 | 2.52E-51 | 9.89E-50 |
| S100A3 | 124.29 | -1.02 | 7.42E-07 | 2.78E-06 |
| TRIM38 | 1025.38 | -1.02 | 1.10E-39 | 2.90E-38 |
| TNFAIP6 | 6.07 | -1.02 | 9.22E-03 | 1.96E-02 |
| UCP2 | 680.48 | -1.02 | 1.03E-27 | 1.59E-26 |
| CCDC28B | 434.47 | -1.02 | 1.85E-17 | 1.60E-16 |
| LPCAT4 | 207.06 | -1.02 | 4.92E-12 | 2.92E-11 |
| PCYOX1L | 303.06 | -1.02 | 7.58E-16 | 5.98E-15 |
| LARP6 | 623.57 | -1.02 | 1.97E-26 | 2.83E-25 |
| SPON2 | 58.90 | -1.02 | 2.84E-05 | 8.88E-05 |
| PEX11A | 121.95 | -1.02 | 1.43E-07 | 5.77E-07 |
| TMEM47 | 284.96 | -1.02 | 6.18E-15 | 4.55E-14 |
| FKBP10 | 1968.47 | -1.02 | 1.37E-47 | 4.87E-46 |
| SCN1B | 407.41 | -1.02 | 4.59E-18 | 4.17E-17 |
| NECAB3 | 1149.59 | -1.03 | 2.38E-34 | 4.92E-33 |
| CNTNAP3 | 131.06 | -1.03 | 1.05E-07 | 4.27E-07 |
| ADCK2 | 485.17 | -1.03 | 8.86E-22 | 9.91E-21 |
| LMBRD1 | 561.12 | -1.03 | 2.32E-23 | 2.81E-22 |
| TMEM35B | 189.25 | -1.03 | 8.15E-11 | 4.41E-10 |
| TRAPPC9 | 632.43 | -1.03 | 1.45E-28 | 2.33E-27 |
| RIPK2 | 271.45 | -1.03 | 8.08E-13 | 5.09E-12 |
| FAM117A | 315.33 | -1.03 | 3.64E-16 | 2.93E-15 |
| CARF | 143.16 | -1.03 | 4.97E-09 | 2.30E-08 |
| HPCAL1 | 1373.02 | -1.03 | 1.34E-44 | 4.28E-43 |
| CNTNAP1 | 1145.49 | -1.03 | 1.26E-27 | 1.94E-26 |
| PKP3 | 698.79 | -1.03 | 5.10E-23 | 6.11E-22 |
| TYMP | 61.84 | -1.03 | 1.03E-04 | 3.01E-04 |
| ARL6IP5 | 1159.15 | -1.03 | 7.40E-41 | 2.06E-39 |
| ACSM3 | 35.31 | -1.03 | 2.75E-04 | 7.55E-04 |
| GPR37 | 11.88 | -1.03 | 5.82E-03 | 1.29E-02 |
| MFSD13A | 227.49 | -1.04 | 3.49E-13 | 2.25E-12 |
| DXO | 291.82 | -1.04 | 1.91E-12 | 1.17E-11 |
| GLRB | 188.11 | -1.04 | 1.33E-10 | 7.08E-10 |
| PLA2G4C | 392.41 | -1.04 | 1.20E-18 | 1.13E-17 |
| DMTN | 336.70 | -1.04 | 1.97E-17 | 1.71E-16 |
| ANK2 | 684.96 | -1.04 | 2.98E-21 | 3.24E-20 |
| H2AC6 | 134.48 | -1.04 | 1.17E-08 | 5.24E-08 |
| ACSL3 | 4622.38 | -1.04 | 9.36E-81 | 8.13E-79 |
| NRTN | 33.71 | -1.04 | 5.30E-04 | 1.39E-03 |
| GFPT1 | 2515.81 | -1.04 | 1.30E-67 | 9.01E-66 |
| IGIP | 88.82 | -1.04 | 8.23E-07 | 3.07E-06 |
| HCN3 | 209.58 | -1.04 | 1.68E-11 | 9.65E-11 |
| CHD9 | 563.47 | -1.04 | 4.37E-18 | 3.98E-17 |
| RAB8B | 764.56 | -1.04 | 2.00E-29 | 3.33E-28 |
| TMX4 | 828.43 | -1.04 | 4.30E-34 | 8.76E-33 |
| C4orf33 | 143.89 | -1.04 | 2.95E-09 | 1.40E-08 |
| NAA80 | 125.95 | -1.04 | 1.57E-08 | 6.93E-08 |
| TMED7-TICAM2 | 240.04 | -1.04 | 9.27E-13 | 5.82E-12 |
| PORCN | 329.96 | -1.04 | 1.07E-16 | 8.93E-16 |
| RAB3A | 102.23 | -1.04 | 3.93E-07 | 1.52E-06 |
| PHKB | 2006.63 | -1.04 | 1.93E-58 | 1.02E-56 |
| TANGO2 | 737.76 | -1.05 | 3.12E-29 | 5.18E-28 |
| UCN | 94.35 | -1.05 | 3.91E-07 | 1.51E-06 |
| ANKRA2 | 138.18 | -1.05 | 4.45E-09 | 2.07E-08 |
| CMC4 | 167.07 | -1.05 | 2.89E-10 | 1.49E-09 |
| NICN1 | 246.73 | -1.05 | 2.18E-13 | 1.43E-12 |
| NEBL | 280.58 | -1.05 | 2.26E-13 | 1.48E-12 |
| ZBTB47 | 160.56 | -1.05 | 1.59E-10 | 8.36E-10 |
| CCDC24 | 153.50 | -1.05 | 2.00E-09 | 9.60E-09 |
| XPOT | 5105.85 | -1.05 | 3.27E-84 | 3.05E-82 |
| SEMA5B | 10.66 | -1.05 | 4.94E-03 | 1.11E-02 |
| TMEM267 | 479.84 | -1.05 | 2.14E-22 | 2.47E-21 |
| FOXO6 | 188.62 | -1.05 | 3.11E-10 | 1.60E-09 |
| SCRN2 | 263.45 | -1.05 | 2.89E-13 | 1.87E-12 |
| HINT3 | 537.48 | -1.06 | 2.00E-22 | 2.32E-21 |
| ELL3 | 35.28 | -1.06 | 2.57E-04 | 7.08E-04 |
| LGALSL | 396.77 | -1.06 | 1.11E-20 | 1.17E-19 |
| COX7A2L | 2515.25 | -1.06 | 2.02E-56 | 9.67E-55 |
| NOTCH2NLA | 1489.46 | -1.06 | 9.60E-43 | 2.88E-41 |
| KAT2B | 376.40 | -1.06 | 2.68E-20 | 2.75E-19 |
| CSGALNACT1 | 34.03 | -1.06 | 4.49E-04 | 1.19E-03 |
| TMEM263 | 1577.01 | -1.06 | 7.82E-54 | 3.34E-52 |
| MYOZ2 | 8.12 | -1.06 | 6.55E-03 | 1.44E-02 |
| MACROD1 | 1425.89 | -1.06 | 3.36E-34 | 6.87E-33 |
| FBXO36 | 175.84 | -1.06 | 3.53E-11 | 1.96E-10 |
| SGCE | 701.89 | -1.06 | 6.37E-30 | 1.08E-28 |
| BCL3 | 788.26 | -1.07 | 8.80E-35 | 1.85E-33 |
| AUH | 295.50 | -1.07 | 5.93E-16 | 4.70E-15 |
| EMP3 | 1635.43 | -1.07 | 3.26E-42 | 9.59E-41 |
| C3orf33 | 142.85 | -1.07 | 1.44E-09 | 7.01E-09 |
| SOCS2 | 19.03 | -1.07 | 1.48E-03 | 3.62E-03 |
| H4C14 | 36.06 | -1.07 | 1.05E-03 | 2.64E-03 |
| PLSCR4 | 237.13 | -1.07 | 2.07E-14 | 1.47E-13 |
| C2 | 18.56 | -1.07 | 1.59E-03 | 3.88E-03 |
| SHFL | 588.58 | -1.07 | 5.77E-27 | 8.53E-26 |
| PLEKHH3 | 682.45 | -1.07 | 9.69E-23 | 1.14E-21 |
| DNASE1L1 | 786.13 | -1.07 | 2.43E-33 | 4.76E-32 |
| IFT74 | 270.06 | -1.07 | 3.75E-15 | 2.81E-14 |
| CHP1 | 2120.50 | -1.07 | 3.27E-63 | 1.96E-61 |
| TRAPPC6A | 544.03 | -1.08 | 8.14E-22 | 9.12E-21 |
| AMN1 | 160.62 | -1.08 | 1.71E-10 | 8.99E-10 |
| LTBP4 | 614.49 | -1.08 | 3.85E-30 | 6.59E-29 |
| KYAT3 | 606.42 | -1.08 | 2.25E-29 | 3.75E-28 |
| MOSPD2 | 928.18 | -1.08 | 6.49E-35 | 1.37E-33 |
| KCTD11 | 323.26 | -1.08 | 2.55E-15 | 1.94E-14 |
| LIPH | 455.89 | -1.08 | 1.38E-23 | 1.69E-22 |
| TMC1 | 4.66 | -1.08 | 7.36E-03 | 1.60E-02 |
| GOLGA8N | 21.82 | -1.08 | 1.32E-03 | 3.26E-03 |
| PPP2R5B | 419.00 | -1.08 | 8.93E-23 | 1.06E-21 |
| PPM1K | 626.78 | -1.08 | 4.24E-25 | 5.64E-24 |
| CDS1 | 205.87 | -1.08 | 2.57E-13 | 1.68E-12 |
| ST3GAL5 | 187.99 | -1.09 | 2.12E-12 | 1.29E-11 |
| CAMTA2 | 538.02 | -1.09 | 9.09E-28 | 1.41E-26 |
| FRRS1 | 472.19 | -1.09 | 1.00E-24 | 1.31E-23 |
| NDNF | 12.50 | -1.09 | 3.46E-03 | 8.00E-03 |
| IFNB1 | 2.43 | -1.09 | 6.29E-03 | 1.38E-02 |
| RBM11 | 27.48 | -1.09 | 4.38E-04 | 1.16E-03 |
| KCNK3 | 64.98 | -1.09 | 8.11E-06 | 2.72E-05 |
| PLOD2 | 1423.08 | -1.09 | 2.19E-44 | 6.93E-43 |
| BIRC3 | 395.50 | -1.09 | 1.16E-19 | 1.17E-18 |
| FAM167B | 40.02 | -1.09 | 2.39E-04 | 6.62E-04 |
| CBR3 | 150.36 | -1.09 | 5.15E-10 | 2.60E-09 |
| FBXO16 | 52.55 | -1.09 | 1.96E-05 | 6.28E-05 |
| TMEM40 | 34.75 | -1.09 | 1.84E-04 | 5.16E-04 |
| SP110 | 766.53 | -1.09 | 5.57E-35 | 1.18E-33 |
| TMOD1 | 52.31 | -1.09 | 3.08E-05 | 9.57E-05 |
| CD22 | 940.97 | -1.10 | 1.96E-41 | 5.57E-40 |
| NPAS1 | 63.99 | -1.10 | 6.21E-06 | 2.11E-05 |
| NIPSNAP1 | 2079.00 | -1.10 | 3.34E-68 | 2.35E-66 |
| COG5 | 313.61 | -1.10 | 4.06E-13 | 2.61E-12 |
| MAP2 | 239.16 | -1.10 | 2.11E-15 | 1.61E-14 |
| DRAM1 | 293.14 | -1.10 | 2.96E-17 | 2.53E-16 |
| RTN4R | 264.69 | -1.10 | 2.00E-15 | 1.54E-14 |
| TTLL1 | 57.85 | -1.10 | 1.69E-05 | 5.45E-05 |
| PGM1 | 2590.00 | -1.10 | 3.46E-76 | 2.79E-74 |
| PPFIA4 | 98.59 | -1.10 | 6.34E-07 | 2.39E-06 |
| ARHGEF3 | 287.15 | -1.10 | 2.03E-16 | 1.67E-15 |
| NUPR1 | 910.15 | -1.10 | 4.20E-40 | 1.13E-38 |
| SLC44A3 | 138.44 | -1.11 | 3.81E-10 | 1.95E-09 |
| RBKS | 37.57 | -1.11 | 1.02E-04 | 2.97E-04 |
| GAL3ST1 | 6.94 | -1.11 | 6.05E-03 | 1.34E-02 |
| BST2 | 3041.63 | -1.11 | 1.65E-57 | 8.38E-56 |
| PTRH1 | 352.58 | -1.11 | 9.75E-19 | 9.28E-18 |
| CRYAB | 271.20 | -1.11 | 4.19E-16 | 3.35E-15 |
| VAMP4 | 155.97 | -1.11 | 3.19E-10 | 1.64E-09 |
| CYP1B1 | 402.98 | -1.11 | 2.28E-22 | 2.62E-21 |
| TMEM231 | 502.96 | -1.11 | 4.02E-24 | 5.06E-23 |
| PCED1B | 35.69 | -1.11 | 2.33E-04 | 6.45E-04 |
| MXRA5 | 100.35 | -1.11 | 3.73E-07 | 1.44E-06 |
| GLB1L | 191.46 | -1.11 | 1.01E-11 | 5.88E-11 |
| ANG | 80.67 | -1.11 | 2.45E-06 | 8.69E-06 |
| KIF6 | 53.32 | -1.11 | 1.10E-05 | 3.65E-05 |
| SLC41A2 | 427.88 | -1.11 | 4.98E-24 | 6.23E-23 |
| TLCD1 | 158.21 | -1.12 | 2.41E-10 | 1.25E-09 |
| ELOVL6 | 1528.40 | -1.12 | 1.07E-48 | 3.90E-47 |
| ASAH1 | 4417.35 | -1.12 | 5.64E-98 | 6.66E-96 |
| TSPAN4 | 845.46 | -1.12 | 3.91E-36 | 8.73E-35 |
| PPA2 | 857.34 | -1.12 | 5.31E-40 | 1.42E-38 |
| MAP3K12 | 294.02 | -1.12 | 6.24E-17 | 5.26E-16 |
| STBD1 | 286.00 | -1.12 | 1.86E-16 | 1.53E-15 |
| PNPLA8 | 941.26 | -1.12 | 4.46E-35 | 9.52E-34 |
| EPHX2 | 278.42 | -1.12 | 1.33E-16 | 1.10E-15 |
| SSTR2 | 100.92 | -1.12 | 9.95E-08 | 4.06E-07 |
| CAMK2A | 19.36 | -1.12 | 1.25E-03 | 3.11E-03 |
| ANPEP | 273.17 | -1.12 | 1.56E-17 | 1.37E-16 |
| ERVFRD-1 | 14.37 | -1.13 | 2.29E-03 | 5.44E-03 |
| ABHD14A-ACY1 | 12.49 | -1.13 | 3.25E-03 | 7.55E-03 |
| MSANTD3-TMEFF1 | 127.61 | -1.13 | 6.38E-08 | 2.67E-07 |
| BMT2 | 228.13 | -1.13 | 8.94E-15 | 6.50E-14 |
| HSD17B14 | 356.13 | -1.13 | 8.16E-18 | 7.28E-17 |
| TNFRSF1A | 1491.55 | -1.13 | 7.44E-50 | 2.80E-48 |
| GRM1 | 6.90 | -1.13 | 5.55E-03 | 1.23E-02 |
| CPT2 | 1024.73 | -1.13 | 4.27E-48 | 1.53E-46 |
| TIMP4 | 46.43 | -1.13 | 2.94E-05 | 9.17E-05 |
| CTAG1B | 1226.52 | -1.13 | 1.89E-04 | 5.29E-04 |
| LAMB3 | 528.96 | -1.13 | 1.33E-28 | 2.14E-27 |
| DUSP19 | 160.71 | -1.14 | 1.76E-11 | 1.01E-10 |
| LHPP | 60.71 | -1.14 | 3.65E-06 | 1.27E-05 |
| SEMA3F | 469.63 | -1.14 | 4.29E-27 | 6.38E-26 |
| NLRC5 | 2494.28 | -1.14 | 6.12E-57 | 2.99E-55 |
| TBC1D17 | 1005.83 | -1.14 | 4.52E-45 | 1.47E-43 |
| TESMIN | 86.68 | -1.14 | 8.78E-08 | 3.61E-07 |
| LAMC3 | 48.32 | -1.14 | 1.66E-05 | 5.34E-05 |
| NIT1 | 687.92 | -1.14 | 3.07E-34 | 6.30E-33 |
| VPS9D1 | 374.59 | -1.14 | 8.36E-20 | 8.41E-19 |
| SMIM19 | 133.07 | -1.14 | 5.91E-10 | 2.97E-09 |
| PHYH | 570.74 | -1.14 | 3.99E-26 | 5.60E-25 |
| SLC30A4 | 193.59 | -1.14 | 1.93E-12 | 1.18E-11 |
| USP32 | 2718.57 | -1.14 | 8.77E-61 | 4.94E-59 |
| NAPSA | 7.49 | -1.14 | 4.85E-03 | 1.09E-02 |
| RAD9B | 21.45 | -1.14 | 1.06E-03 | 2.65E-03 |
| BBOF1 | 76.19 | -1.14 | 8.73E-07 | 3.24E-06 |
| TCEA2 | 493.96 | -1.14 | 6.02E-20 | 6.10E-19 |
| CPQ | 225.93 | -1.14 | 1.81E-14 | 1.29E-13 |
| TMEM229B | 8.60 | -1.14 | 4.80E-03 | 1.08E-02 |
| CDH2 | 2453.47 | -1.14 | 3.52E-74 | 2.76E-72 |
| PRICKLE3 | 548.86 | -1.15 | 8.25E-31 | 1.45E-29 |
| TENT5C | 25.84 | -1.15 | 4.07E-04 | 1.09E-03 |
| SLC25A35 | 190.28 | -1.15 | 3.41E-12 | 2.05E-11 |
| ASMTL | 700.25 | -1.15 | 3.01E-35 | 6.47E-34 |
| CLYBL | 190.53 | -1.15 | 1.19E-12 | 7.42E-12 |
| MMUT | 731.51 | -1.15 | 1.98E-39 | 5.16E-38 |
| ARL1 | 1087.68 | -1.15 | 1.51E-42 | 4.50E-41 |
| SHISA4 | 276.26 | -1.15 | 5.45E-18 | 4.93E-17 |
| LYST | 590.03 | -1.15 | 1.07E-23 | 1.32E-22 |
| RRAS | 368.08 | -1.15 | 5.92E-18 | 5.35E-17 |
| ADSS1 | 7.50 | -1.16 | 4.56E-03 | 1.03E-02 |
| PHF11 | 352.67 | -1.16 | 4.31E-20 | 4.39E-19 |
| TGFB1 | 3223.40 | -1.16 | 1.37E-73 | 1.06E-71 |
| SMIM30 | 521.64 | -1.16 | 3.33E-27 | 4.99E-26 |
| RUSF1 | 1346.81 | -1.16 | 3.89E-53 | 1.64E-51 |
| NINJ1 | 1099.42 | -1.16 | 7.37E-51 | 2.83E-49 |
| SLC38A6 | 397.52 | -1.16 | 8.31E-24 | 1.03E-22 |
| FADS1 | 4436.01 | -1.16 | 6.40E-100 | 7.85E-98 |
| GBP3 | 230.03 | -1.16 | 1.88E-14 | 1.34E-13 |
| KLHL38 | 302.06 | -1.16 | 4.19E-18 | 3.82E-17 |
| NPAS2 | 274.07 | -1.16 | 7.65E-18 | 6.84E-17 |
| PRPF40B | 145.83 | -1.16 | 2.20E-11 | 1.24E-10 |
| ACAD11 | 802.02 | -1.16 | 2.41E-41 | 6.83E-40 |
| CCPG1 | 1637.17 | -1.16 | 7.41E-56 | 3.44E-54 |
| OGDHL | 697.66 | -1.17 | 1.37E-35 | 2.98E-34 |
| METRN | 1444.47 | -1.17 | 5.45E-56 | 2.56E-54 |
| ABHD4 | 1102.28 | -1.17 | 4.49E-51 | 1.74E-49 |
| GPCPD1 | 436.05 | -1.17 | 8.26E-26 | 1.13E-24 |
| VPS37D | 40.90 | -1.17 | 4.10E-05 | 1.26E-04 |
| LAP3 | 4152.11 | -1.17 | 5.08E-105 | 6.70E-103 |
| MARCHF9 | 302.19 | -1.17 | 2.03E-17 | 1.76E-16 |
| ZBTB25 | 278.76 | -1.17 | 3.32E-18 | 3.04E-17 |
| TRPV2 | 26.32 | -1.17 | 7.28E-04 | 1.88E-03 |
| ZFP36 | 336.67 | -1.17 | 9.21E-21 | 9.69E-20 |
| SDHAF4 | 122.07 | -1.18 | 2.96E-10 | 1.52E-09 |
| MXI1 | 1028.42 | -1.18 | 3.27E-42 | 9.59E-41 |
| DDO | 6.47 | -1.18 | 5.00E-03 | 1.12E-02 |
| GSTZ1 | 142.53 | -1.18 | 1.61E-09 | 7.79E-09 |
| JHY | 54.81 | -1.18 | 3.15E-06 | 1.11E-05 |
| CHPT1 | 819.71 | -1.18 | 2.18E-39 | 5.68E-38 |
| CCL28 | 834.54 | -1.18 | 2.34E-42 | 6.92E-41 |
| LIPA | 1163.58 | -1.18 | 7.52E-53 | 3.13E-51 |
| BTBD8 | 162.89 | -1.18 | 6.61E-12 | 3.89E-11 |
| GGACT | 121.68 | -1.18 | 2.41E-10 | 1.25E-09 |
| SMYD3 | 522.92 | -1.18 | 1.39E-26 | 2.01E-25 |
| YIPF2 | 968.62 | -1.18 | 2.40E-37 | 5.69E-36 |
| CPEB3 | 93.05 | -1.18 | 1.91E-08 | 8.34E-08 |
| MYO18B | 625.67 | -1.18 | 1.77E-28 | 2.84E-27 |
| CTH | 723.95 | -1.19 | 2.51E-24 | 3.20E-23 |
| TK2 | 655.63 | -1.19 | 4.09E-36 | 9.11E-35 |
| KCNE3 | 12.14 | -1.19 | 2.46E-03 | 5.83E-03 |
| FAM149A | 13.47 | -1.19 | 1.71E-03 | 4.16E-03 |
| PGGHG | 186.34 | -1.19 | 6.33E-12 | 3.74E-11 |
| RTN2 | 490.02 | -1.19 | 5.09E-30 | 8.68E-29 |
| CLU | 2210.02 | -1.19 | 3.69E-58 | 1.92E-56 |
| ZNF524 | 191.85 | -1.19 | 4.71E-14 | 3.24E-13 |
| FBXO2 | 72.79 | -1.20 | 1.89E-07 | 7.52E-07 |
| TSPAN31 | 485.68 | -1.20 | 7.94E-29 | 1.29E-27 |
| KLHL35 | 73.31 | -1.20 | 3.55E-07 | 1.38E-06 |
| MYZAP | 21.24 | -1.20 | 4.73E-04 | 1.25E-03 |
| SNX10 | 284.61 | -1.20 | 5.38E-18 | 4.88E-17 |
| PIP4P2 | 489.56 | -1.21 | 8.14E-26 | 1.12E-24 |
| TMEM187 | 178.24 | -1.21 | 2.71E-13 | 1.76E-12 |
| BSCL2 | 255.40 | -1.21 | 3.38E-16 | 2.72E-15 |
| DYNC2I1 | 339.19 | -1.21 | 3.29E-21 | 3.57E-20 |
| TRADD | 708.53 | -1.21 | 2.59E-38 | 6.41E-37 |
| LGALS3BP | 9793.00 | -1.21 | 3.61E-146 | 9.04E-144 |
| IQCH | 81.05 | -1.21 | 7.62E-08 | 3.16E-07 |
| NRSN2 | 713.23 | -1.22 | 8.96E-39 | 2.27E-37 |
| IQCD | 132.87 | -1.22 | 8.39E-11 | 4.53E-10 |
| SLC66A3 | 335.57 | -1.22 | 2.46E-22 | 2.83E-21 |
| TUBG2 | 390.48 | -1.22 | 2.87E-25 | 3.84E-24 |
| SEPTIN4 | 17.45 | -1.22 | 7.28E-04 | 1.88E-03 |
| SCN3A | 11.94 | -1.22 | 2.51E-03 | 5.92E-03 |
| NNMT | 284.10 | -1.22 | 1.77E-18 | 1.65E-17 |
| SPINT1 | 9.90 | -1.22 | 3.05E-03 | 7.11E-03 |
| ABCA3 | 681.00 | -1.22 | 1.35E-33 | 2.68E-32 |
| NAMPT | 2516.46 | -1.22 | 4.30E-89 | 4.30E-87 |
| LOX | 1444.62 | -1.22 | 1.25E-65 | 7.97E-64 |
| HAGHL | 714.73 | -1.22 | 2.37E-31 | 4.28E-30 |
| NCK1 | 881.77 | -1.22 | 1.03E-46 | 3.55E-45 |
| SIDT2 | 808.32 | -1.22 | 1.24E-42 | 3.69E-41 |
| APOL2 | 1234.84 | -1.22 | 1.42E-53 | 6.05E-52 |
| MID2 | 521.37 | -1.22 | 2.57E-27 | 3.88E-26 |
| GMPR2 | 379.76 | -1.22 | 2.99E-13 | 1.94E-12 |
| JUP | 107.12 | -1.22 | 3.77E-09 | 1.77E-08 |
| MYL5 | 135.23 | -1.22 | 3.04E-10 | 1.56E-09 |
| C4B | 66.71 | -1.22 | 4.84E-03 | 1.09E-02 |
| STAT2 | 1683.81 | -1.22 | 1.96E-67 | 1.36E-65 |
| TRIM6 | 316.03 | -1.23 | 4.06E-22 | 4.63E-21 |
| PDK4 | 48.87 | -1.23 | 1.43E-05 | 4.67E-05 |
| RNASE4 | 126.00 | -1.23 | 1.51E-10 | 7.99E-10 |
| PC | 2324.01 | -1.23 | 2.53E-82 | 2.31E-80 |
| OCLN | 952.34 | -1.23 | 1.38E-39 | 3.63E-38 |
| ME1 | 1601.81 | -1.23 | 3.16E-58 | 1.65E-56 |
| SLC46A3 | 408.86 | -1.23 | 1.87E-24 | 2.41E-23 |
| FBXO8 | 249.71 | -1.23 | 1.86E-16 | 1.53E-15 |
| YPEL5 | 1337.93 | -1.23 | 4.98E-56 | 2.34E-54 |
| JAZF1 | 240.27 | -1.23 | 8.95E-18 | 7.94E-17 |
| NPIPA5 | 4.60 | -1.23 | 4.42E-03 | 1.00E-02 |
| PSG3 | 60.94 | -1.23 | 2.06E-06 | 7.38E-06 |
| GARS1 | 5691.79 | -1.23 | 2.41E-145 | 5.94E-143 |
| HSD11B1 | 3.79 | -1.24 | 4.52E-03 | 1.02E-02 |
| NYAP2 | 591.75 | -1.24 | 4.92E-38 | 1.19E-36 |
| CRYL1 | 188.32 | -1.24 | 4.11E-15 | 3.06E-14 |
| C3orf18 | 149.64 | -1.24 | 1.74E-12 | 1.07E-11 |
| NCOA7 | 1052.86 | -1.24 | 2.18E-52 | 8.91E-51 |
| ATOSB | 415.35 | -1.24 | 8.21E-26 | 1.13E-24 |
| IL18R1 | 42.42 | -1.24 | 1.06E-05 | 3.49E-05 |
| SDAD1P1 | 103.35 | -1.24 | 3.05E-09 | 1.44E-08 |
| CPT1B | 45.89 | -1.24 | 1.40E-05 | 4.58E-05 |
| COG6 | 739.24 | -1.25 | 4.97E-40 | 1.33E-38 |
| LRRC4C | 3.79 | -1.25 | 4.41E-03 | 9.98E-03 |
| ADM2 | 188.76 | -1.25 | 2.37E-15 | 1.80E-14 |
| PSG9 | 579.41 | -1.25 | 3.05E-27 | 4.58E-26 |
| NAGLU | 977.55 | -1.25 | 8.26E-52 | 3.29E-50 |
| ZCCHC2 | 374.09 | -1.25 | 3.15E-24 | 3.99E-23 |
| FAM151A | 5.21 | -1.25 | 4.54E-03 | 1.03E-02 |
| ABLIM2 | 204.47 | -1.25 | 1.71E-14 | 1.22E-13 |
| MAGED2 | 3075.84 | -1.25 | 3.29E-94 | 3.67E-92 |
| WDR31 | 221.15 | -1.25 | 7.96E-17 | 6.68E-16 |
| GSDMD | 853.45 | -1.25 | 7.00E-08 | 2.91E-07 |
| ACTA2 | 4028.99 | -1.25 | 7.46E-17 | 6.26E-16 |
| PDK2 | 1108.11 | -1.25 | 2.16E-54 | 9.45E-53 |
| PXK | 343.72 | -1.25 | 2.39E-23 | 2.89E-22 |
| ARRDC4 | 117.82 | -1.25 | 1.08E-09 | 5.32E-09 |
| LPAR5 | 102.14 | -1.25 | 3.76E-09 | 1.76E-08 |
| SCD | 20473.36 | -1.26 | 1.81E-126 | 3.48E-124 |
| ABCG2 | 45.86 | -1.26 | 1.52E-05 | 4.92E-05 |
| ANKEF1 | 241.05 | -1.26 | 6.00E-17 | 5.06E-16 |
| NOS3 | 577.12 | -1.26 | 4.26E-37 | 9.96E-36 |
| DNAJB5 | 494.94 | -1.26 | 6.84E-30 | 1.16E-28 |
| NR1H3 | 202.35 | -1.26 | 5.57E-15 | 4.12E-14 |
| ADGRE1 | 66.33 | -1.26 | 2.31E-07 | 9.16E-07 |
| INSIG2 | 991.04 | -1.26 | 5.40E-47 | 1.88E-45 |
| TEX19 | 79.84 | -1.26 | 2.89E-07 | 1.13E-06 |
| ZNF564 | 64.83 | -1.26 | 1.52E-05 | 4.94E-05 |
| GGT1 | 459.09 | -1.27 | 7.88E-21 | 8.32E-20 |
| PSMB10 | 598.73 | -1.27 | 1.28E-33 | 2.54E-32 |
| PTPRH | 51.20 | -1.27 | 1.72E-06 | 6.22E-06 |
| ATP8A1 | 87.33 | -1.27 | 3.72E-09 | 1.74E-08 |
| IL17RC | 644.03 | -1.27 | 7.52E-36 | 1.65E-34 |
| SLC17A5 | 1341.99 | -1.27 | 5.66E-69 | 4.03E-67 |
| RRAGD | 1378.47 | -1.27 | 1.14E-64 | 7.03E-63 |
| SLC25A23 | 2170.64 | -1.28 | 1.45E-85 | 1.40E-83 |
| NPEPPSP1 | 11.74 | -1.28 | 3.14E-03 | 7.31E-03 |
| PRODH | 45.24 | -1.28 | 8.31E-06 | 2.78E-05 |
| SUCLG2 | 1022.31 | -1.28 | 1.38E-56 | 6.65E-55 |
| CLIC2 | 481.39 | -1.28 | 4.50E-27 | 6.69E-26 |
| DALRD3 | 530.46 | -1.28 | 6.09E-35 | 1.29E-33 |
| PRPH | 2.86 | -1.28 | 3.72E-03 | 8.55E-03 |
| TAPBPL | 69.25 | -1.28 | 4.96E-07 | 1.90E-06 |
| TCP11L2 | 65.40 | -1.28 | 1.52E-07 | 6.13E-07 |
| RGS9 | 361.90 | -1.29 | 3.96E-26 | 5.58E-25 |
| GPRASP3 | 29.34 | -1.29 | 9.64E-05 | 2.82E-04 |
| TMEM141 | 654.60 | -1.29 | 2.41E-35 | 5.19E-34 |
| LRSAM1 | 731.38 | -1.29 | 5.29E-42 | 1.54E-40 |
| BNIP3L | 1914.45 | -1.30 | 7.66E-66 | 4.94E-64 |
| AK4 | 1458.96 | -1.30 | 8.76E-61 | 4.94E-59 |
| FBXL8 | 350.00 | -1.30 | 1.32E-26 | 1.91E-25 |
| H1-2 | 303.75 | -1.30 | 5.15E-19 | 4.99E-18 |
| HES4 | 891.54 | -1.30 | 1.23E-43 | 3.80E-42 |
| HFE | 388.96 | -1.30 | 2.22E-27 | 3.37E-26 |
| TMEM8B | 185.51 | -1.30 | 8.63E-16 | 6.77E-15 |
| TDRD7 | 792.07 | -1.30 | 6.41E-49 | 2.35E-47 |
| LRP11 | 971.76 | -1.30 | 4.16E-56 | 1.96E-54 |
| CNOT6LP1 | 14.23 | -1.30 | 9.52E-04 | 2.41E-03 |
| ACSL5 | 208.07 | -1.30 | 3.86E-17 | 3.29E-16 |
| NAT16 | 15.53 | -1.30 | 8.11E-04 | 2.08E-03 |
| ATP4A | 5.10 | -1.30 | 3.91E-03 | 8.94E-03 |
| PIGV | 297.65 | -1.30 | 3.32E-19 | 3.25E-18 |
| RABAC1 | 1601.75 | -1.31 | 3.50E-78 | 2.91E-76 |
| SPATA7 | 53.11 | -1.31 | 7.02E-07 | 2.64E-06 |
| ALPK1 | 187.35 | -1.31 | 3.12E-16 | 2.53E-15 |
| TMEM120A | 365.71 | -1.31 | 2.53E-24 | 3.23E-23 |
| CCDC121 | 127.34 | -1.31 | 7.29E-11 | 3.95E-10 |
| MSMO1 | 4099.19 | -1.31 | 3.09E-67 | 2.09E-65 |
| KAZALD1 | 84.92 | -1.31 | 3.04E-09 | 1.44E-08 |
| HERPUD1 | 2162.16 | -1.31 | 2.42E-97 | 2.81E-95 |
| DDX60L | 1563.22 | -1.31 | 7.61E-71 | 5.64E-69 |
| TRIM14 | 1682.06 | -1.31 | 1.98E-55 | 9.00E-54 |
| TNNT1 | 683.44 | -1.31 | 2.01E-34 | 4.18E-33 |
| TOB1 | 477.34 | -1.32 | 1.14E-29 | 1.91E-28 |
| TLR3 | 85.48 | -1.32 | 3.30E-09 | 1.55E-08 |
| RARG | 1007.63 | -1.32 | 2.72E-49 | 1.00E-47 |
| BHLHE41 | 522.90 | -1.32 | 1.79E-31 | 3.24E-30 |
| TRIQK | 130.00 | -1.32 | 4.11E-12 | 2.46E-11 |
| TRIM21 | 190.08 | -1.32 | 9.75E-17 | 8.15E-16 |
| GRAMD1B | 4.99 | -1.32 | 3.72E-03 | 8.54E-03 |
| DRAM2 | 658.34 | -1.32 | 6.03E-40 | 1.60E-38 |
| H2AJ | 428.58 | -1.32 | 2.03E-23 | 2.47E-22 |
| RGS6 | 3.97 | -1.32 | 3.62E-03 | 8.33E-03 |
| HRH2 | 623.69 | -1.32 | 4.32E-39 | 1.11E-37 |
| METTL25B | 522.72 | -1.32 | 7.59E-34 | 1.54E-32 |
| BTN3A1 | 707.37 | -1.32 | 4.42E-42 | 1.29E-40 |
| NAP1L5 | 252.02 | -1.32 | 2.20E-17 | 1.90E-16 |
| STK17B | 841.95 | -1.33 | 3.69E-50 | 1.40E-48 |
| NEFL | 99.84 | -1.33 | 9.59E-10 | 4.74E-09 |
| TLR5 | 15.00 | -1.33 | 1.03E-03 | 2.60E-03 |
| FIBCD1 | 496.48 | -1.33 | 3.01E-34 | 6.18E-33 |
| SPACA9 | 172.64 | -1.33 | 1.36E-13 | 9.11E-13 |
| NMRK1 | 293.56 | -1.33 | 2.15E-23 | 2.61E-22 |
| B2M | 16207.63 | -1.33 | 7.46E-124 | 1.33E-121 |
| SLC25A1 | 2687.94 | -1.33 | 1.07E-114 | 1.65E-112 |
| DECR2 | 380.88 | -1.33 | 2.33E-21 | 2.55E-20 |
| RAP1GAP | 84.98 | -1.34 | 2.72E-09 | 1.29E-08 |
| PDP2 | 1620.67 | -1.34 | 2.41E-85 | 2.30E-83 |
| SELENBP1 | 409.96 | -1.34 | 1.99E-28 | 3.18E-27 |
| GSN | 1001.62 | -1.34 | 4.99E-47 | 1.74E-45 |
| OXCT1 | 2685.51 | -1.35 | 6.11E-113 | 9.25E-111 |
| CCDC110 | 16.26 | -1.35 | 5.35E-04 | 1.40E-03 |
| PLXNC1 | 102.96 | -1.35 | 5.22E-11 | 2.87E-10 |
| EFEMP1 | 429.62 | -1.35 | 8.00E-33 | 1.54E-31 |
| AARS1 | 15268.38 | -1.35 | 1.41E-180 | 4.58E-178 |
| MOB3C | 196.22 | -1.35 | 2.83E-18 | 2.60E-17 |
| STAT4 | 17.17 | -1.36 | 5.09E-04 | 1.34E-03 |
| ACY1 | 578.69 | -1.36 | 7.76E-37 | 1.78E-35 |
| EPOR | 292.73 | -1.36 | 5.32E-24 | 6.64E-23 |
| NUDT18 | 156.56 | -1.36 | 2.31E-14 | 1.63E-13 |
| IFNAR2-IL10RB | 2.77 | -1.37 | 3.11E-03 | 7.24E-03 |
| TSPAN13 | 61.56 | -1.37 | 2.41E-07 | 9.49E-07 |
| PARP14 | 3508.87 | -1.37 | 2.40E-106 | 3.25E-104 |
| BCAN | 167.78 | -1.37 | 5.90E-16 | 4.68E-15 |
| TRPA1 | 34.02 | -1.37 | 3.36E-05 | 1.04E-04 |
| MTHFR | 2264.72 | -1.37 | 2.75E-107 | 3.85E-105 |
| PTK2B | 109.56 | -1.37 | 1.55E-10 | 8.19E-10 |
| NECAB1 | 189.42 | -1.37 | 2.73E-16 | 2.21E-15 |
| TCEANC | 59.95 | -1.37 | 2.46E-07 | 9.71E-07 |
| DBH | 73.27 | -1.38 | 7.15E-09 | 3.27E-08 |
| NR3C2 | 38.22 | -1.38 | 6.76E-06 | 2.29E-05 |
| PLIN2 | 1351.68 | -1.38 | 4.47E-59 | 2.39E-57 |
| RILP | 233.95 | -1.38 | 8.09E-19 | 7.74E-18 |
| SERINC2 | 960.40 | -1.38 | 4.46E-66 | 2.90E-64 |
| MIF4GD | 311.91 | -1.39 | 1.92E-26 | 2.77E-25 |
| CERT1 | 981.35 | -1.39 | 8.71E-64 | 5.27E-62 |
| GOLGA5 | 1035.65 | -1.39 | 7.55E-68 | 5.28E-66 |
| PRDM16 | 5.93 | -1.39 | 2.97E-03 | 6.94E-03 |
| DPYD | 184.31 | -1.39 | 2.90E-17 | 2.49E-16 |
| HLA-E | 3645.51 | -1.39 | 1.94E-75 | 1.55E-73 |
| RALGDS | 2523.99 | -1.40 | 3.46E-125 | 6.28E-123 |
| HCFC1R1 | 1204.05 | -1.40 | 5.92E-53 | 2.47E-51 |
| FAS | 260.30 | -1.40 | 1.57E-17 | 1.37E-16 |
| ZNFX1 | 2186.10 | -1.40 | 1.14E-62 | 6.68E-61 |
| TXLNB | 9.35 | -1.40 | 1.70E-03 | 4.14E-03 |
| RAB27B | 199.29 | -1.40 | 2.88E-18 | 2.65E-17 |
| RRAD | 134.87 | -1.41 | 1.94E-10 | 1.01E-09 |
| PDGFA | 290.80 | -1.41 | 5.91E-17 | 4.99E-16 |
| MICALL2 | 499.01 | -1.41 | 2.39E-38 | 5.95E-37 |
| CERCAM | 694.56 | -1.41 | 9.02E-50 | 3.38E-48 |
| SP100 | 1696.17 | -1.41 | 1.22E-95 | 1.40E-93 |
| HMGCL | 837.42 | -1.41 | 2.44E-50 | 9.32E-49 |
| S100A6 | 3355.41 | -1.41 | 2.30E-67 | 1.57E-65 |
| ESRP1 | 9.46 | -1.41 | 1.72E-03 | 4.18E-03 |
| NUCB2 | 1225.46 | -1.41 | 8.92E-56 | 4.09E-54 |
| CACNA1H | 2.89 | -1.41 | 2.77E-03 | 6.49E-03 |
| GPM6A | 11.00 | -1.42 | 1.04E-03 | 2.62E-03 |
| ZNF25 | 143.58 | -1.42 | 4.51E-15 | 3.36E-14 |
| TMEM53 | 172.83 | -1.42 | 1.53E-17 | 1.34E-16 |
| NR4A3 | 46.56 | -1.42 | 7.03E-07 | 2.64E-06 |
| HIBADH | 563.75 | -1.42 | 8.73E-36 | 1.91E-34 |
| PRUNE2 | 2775.10 | -1.43 | 7.09E-21 | 7.50E-20 |
| FMNL1 | 1488.03 | -1.43 | 5.58E-91 | 5.72E-89 |
| ZC3H6 | 139.82 | -1.43 | 7.45E-15 | 5.46E-14 |
| PSG4 | 63.19 | -1.43 | 1.50E-08 | 6.66E-08 |
| PTH1R | 9.31 | -1.43 | 1.48E-03 | 3.63E-03 |
| PRR7 | 622.28 | -1.43 | 3.30E-39 | 8.51E-38 |
| TNS2 | 708.89 | -1.44 | 8.40E-56 | 3.87E-54 |
| TMEM135 | 674.74 | -1.44 | 1.68E-50 | 6.45E-49 |
| SREBF1 | 2548.25 | -1.44 | 2.52E-119 | 4.16E-117 |
| CTSB | 7896.31 | -1.44 | 4.01E-188 | 1.47E-185 |
| CASKIN1 | 88.61 | -1.44 | 1.25E-10 | 6.66E-10 |
| SYT13 | 55.76 | -1.44 | 1.28E-06 | 4.68E-06 |
| GMPR | 358.57 | -1.45 | 5.77E-32 | 1.07E-30 |
| EIF2AK2 | 4221.31 | -1.45 | 3.30E-119 | 5.39E-117 |
| CD70 | 149.41 | -1.45 | 1.78E-15 | 1.37E-14 |
| MAPRE3 | 612.45 | -1.45 | 2.84E-45 | 9.35E-44 |
| SHC2 | 98.68 | -1.45 | 1.08E-10 | 5.76E-10 |
| SLC2A10 | 371.58 | -1.45 | 2.33E-33 | 4.56E-32 |
| SIGIRR | 245.81 | -1.45 | 6.02E-21 | 6.42E-20 |
| FBXO4 | 426.66 | -1.45 | 2.37E-32 | 4.49E-31 |
| BTN3A3 | 403.80 | -1.46 | 1.29E-35 | 2.81E-34 |
| LGALS9 | 28.25 | -1.46 | 2.96E-05 | 9.23E-05 |
| MN1 | 213.79 | -1.46 | 4.91E-19 | 4.76E-18 |
| BICDL1 | 211.91 | -1.46 | 1.05E-17 | 9.24E-17 |
| PPM1M | 239.40 | -1.46 | 8.12E-24 | 1.01E-22 |
| BTN3A2 | 1277.41 | -1.46 | 2.55E-81 | 2.24E-79 |
| WNT6 | 359.69 | -1.46 | 1.23E-23 | 1.52E-22 |
| WIPI1 | 1156.39 | -1.47 | 1.72E-72 | 1.31E-70 |
| HSF4 | 712.87 | -1.47 | 3.50E-51 | 1.37E-49 |
| GALK1 | 455.45 | -1.47 | 4.13E-36 | 9.19E-35 |
| MFSD3 | 789.33 | -1.47 | 4.28E-52 | 1.72E-50 |
| STARD4 | 4428.59 | -1.48 | 1.55E-152 | 3.99E-150 |
| SMIM29 | 361.18 | -1.48 | 1.14E-30 | 2.00E-29 |
| BAIAP2L2 | 50.83 | -1.48 | 4.98E-07 | 1.90E-06 |
| PGLS | 892.27 | -1.48 | 2.97E-56 | 1.42E-54 |
| HCN2 | 325.53 | -1.48 | 2.95E-26 | 4.19E-25 |
| EIF4E3 | 124.78 | -1.49 | 1.51E-12 | 9.31E-12 |
| FAM86B3P | 2.93 | -1.49 | 2.37E-03 | 5.62E-03 |
| INHBC | 8.58 | -1.49 | 1.29E-03 | 3.19E-03 |
| SCNN1D | 36.85 | -1.49 | 2.29E-06 | 8.16E-06 |
| MAPK11 | 315.24 | -1.49 | 1.17E-28 | 1.90E-27 |
| SLCO2B1 | 70.24 | -1.49 | 1.55E-08 | 6.84E-08 |
| CYBRD1 | 2368.52 | -1.50 | 9.86E-134 | 2.01E-131 |
| PERM1 | 9.01 | -1.50 | 1.33E-03 | 3.28E-03 |
| TREX1 | 337.77 | -1.51 | 4.35E-30 | 7.45E-29 |
| TESK2 | 290.68 | -1.51 | 3.18E-28 | 5.02E-27 |
| AJM1 | 371.91 | -1.51 | 3.48E-28 | 5.50E-27 |
| GPNMB | 24191.40 | -1.51 | 6.43E-263 | 5.31E-260 |
| GOLGA7B | 79.42 | -1.51 | 1.62E-10 | 8.55E-10 |
| HOXD8 | 230.28 | -1.52 | 4.28E-24 | 5.38E-23 |
| CALCOCO2 | 1483.65 | -1.52 | 4.77E-88 | 4.75E-86 |
| THBS3 | 1241.88 | -1.52 | 9.60E-82 | 8.67E-80 |
| IDH2 | 3362.30 | -1.53 | 1.90E-157 | 5.14E-155 |
| NPY6R | 7.98 | -1.53 | 1.29E-03 | 3.19E-03 |
| FABP3 | 1670.49 | -1.53 | 1.20E-69 | 8.74E-68 |
| IL15RA | 148.01 | -1.53 | 3.50E-14 | 2.43E-13 |
| TMTC1 | 602.69 | -1.54 | 3.00E-42 | 8.83E-41 |
| ACADS | 185.70 | -1.54 | 5.92E-18 | 5.35E-17 |
| TLE6 | 16.57 | -1.54 | 4.10E-04 | 1.10E-03 |
| ABCA7 | 494.91 | -1.54 | 6.82E-45 | 2.19E-43 |
| MVP | 1963.77 | -1.54 | 5.67E-107 | 7.87E-105 |
| OCEL1 | 127.21 | -1.55 | 1.13E-14 | 8.19E-14 |
| SEC24D | 1821.70 | -1.55 | 2.17E-103 | 2.83E-101 |
| SEMA4B | 986.45 | -1.55 | 1.92E-63 | 1.16E-61 |
| SERPINA5 | 17.00 | -1.55 | 1.87E-04 | 5.24E-04 |
| TMEM255A | 354.63 | -1.55 | 4.88E-33 | 9.48E-32 |
| CTSD | 15985.77 | -1.55 | 2.41E-253 | 1.81E-250 |
| PARP12 | 877.31 | -1.55 | 5.09E-72 | 3.82E-70 |
| FBXO6 | 225.80 | -1.55 | 2.38E-24 | 3.05E-23 |
| MGP | 61.66 | -1.55 | 3.28E-09 | 1.55E-08 |
| RNPEPL1 | 1570.18 | -1.55 | 1.87E-114 | 2.86E-112 |
| MR1 | 267.19 | -1.55 | 9.45E-24 | 1.17E-22 |
| KDELR3 | 342.36 | -1.56 | 6.12E-35 | 1.30E-33 |
| SPATA20 | 828.27 | -1.56 | 3.93E-62 | 2.29E-60 |
| ATP1A2 | 88.69 | -1.56 | 1.58E-11 | 9.05E-11 |
| ABHD14B | 1308.02 | -1.56 | 6.10E-92 | 6.37E-90 |
| EGFLAM | 8.98 | -1.56 | 1.03E-03 | 2.59E-03 |
| CASTOR1 | 71.04 | -1.56 | 7.24E-10 | 3.61E-09 |
| LMTK3 | 45.22 | -1.56 | 2.30E-07 | 9.10E-07 |
| HMOX1 | 853.48 | -1.57 | 1.41E-49 | 5.24E-48 |
| DTX3L | 2887.05 | -1.57 | 1.23E-115 | 1.96E-113 |
| GRIP2 | 93.32 | -1.57 | 2.02E-12 | 1.24E-11 |
| CERS4 | 627.41 | -1.57 | 5.10E-49 | 1.87E-47 |
| ZCWPW2 | 17.77 | -1.58 | 1.25E-04 | 3.60E-04 |
| SEPTIN1 | 22.96 | -1.58 | 3.56E-05 | 1.10E-04 |
| CEBPB | 2333.24 | -1.58 | 1.62E-98 | 1.94E-96 |
| NRCAM | 378.66 | -1.58 | 1.57E-36 | 3.56E-35 |
| KRCC1 | 214.19 | -1.58 | 4.60E-25 | 6.11E-24 |
| SMIM10L2B | 19.55 | -1.59 | 6.04E-05 | 1.81E-04 |
| VWA5A | 180.89 | -1.59 | 5.16E-22 | 5.85E-21 |
| IDH1 | 4439.09 | -1.59 | 1.33E-170 | 4.05E-168 |
| NDRG1 | 8589.07 | -1.60 | 1.66E-141 | 3.70E-139 |
| ACP3 | 7.05 | -1.60 | 1.43E-03 | 3.51E-03 |
| SLC50A1 | 1059.90 | -1.60 | 5.80E-92 | 6.10E-90 |
| SLFN5 | 1427.62 | -1.60 | 1.87E-77 | 1.53E-75 |
| C1QTNF6 | 605.55 | -1.61 | 5.35E-47 | 1.86E-45 |
| TSLP | 42.36 | -1.61 | 2.83E-07 | 1.11E-06 |
| CLIP3 | 856.54 | -1.61 | 4.31E-55 | 1.93E-53 |
| PDK1 | 849.75 | -1.62 | 3.99E-70 | 2.93E-68 |
| TMEM37 | 28.84 | -1.62 | 8.14E-06 | 2.73E-05 |
| PIGZ | 181.06 | -1.62 | 2.48E-21 | 2.71E-20 |
| CTBS | 768.88 | -1.63 | 2.47E-66 | 1.62E-64 |
| RBM4 | 32.36 | -1.63 | 4.42E-06 | 1.53E-05 |
| ICA1 | 174.79 | -1.63 | 2.36E-20 | 2.44E-19 |
| FGF19 | 9.93 | -1.63 | 6.54E-04 | 1.70E-03 |
| F8 | 426.72 | -1.64 | 3.52E-45 | 1.15E-43 |
| CEBPD | 390.85 | -1.64 | 1.77E-33 | 3.50E-32 |
| TP53INP2 | 1311.18 | -1.64 | 2.20E-93 | 2.39E-91 |
| CADPS2 | 88.96 | -1.64 | 3.37E-12 | 2.03E-11 |
| APOLD1 | 964.30 | -1.65 | 2.64E-82 | 2.39E-80 |
| SLC16A13 | 189.70 | -1.65 | 1.01E-20 | 1.07E-19 |
| UBQLNL | 3.12 | -1.66 | 1.64E-03 | 4.01E-03 |
| SULT1C2 | 943.64 | -1.66 | 4.47E-78 | 3.69E-76 |
| RUNDC3B | 64.32 | -1.66 | 3.90E-10 | 1.99E-09 |
| SSC4D | 38.37 | -1.67 | 1.01E-06 | 3.74E-06 |
| SLC6A8 | 973.50 | -1.67 | 1.07E-67 | 7.48E-66 |
| HSD3B7 | 842.27 | -1.67 | 2.48E-81 | 2.19E-79 |
| ANKRD29 | 94.06 | -1.68 | 1.04E-13 | 7.00E-13 |
| TMPRSS3 | 23.67 | -1.68 | 1.19E-05 | 3.92E-05 |
| TRIML2 | 496.68 | -1.69 | 1.76E-51 | 6.92E-50 |
| SERPINB1 | 956.28 | -1.69 | 3.52E-83 | 3.23E-81 |
| JAKMIP2 | 61.26 | -1.69 | 7.99E-10 | 3.97E-09 |
| SRR | 247.70 | -1.70 | 4.79E-30 | 8.18E-29 |
| IL34 | 304.42 | -1.70 | 1.83E-32 | 3.49E-31 |
| HPN | 18.37 | -1.70 | 8.83E-05 | 2.59E-04 |
| TNFSF9 | 294.35 | -1.70 | 1.02E-33 | 2.04E-32 |
| GSAP | 28.77 | -1.71 | 2.57E-06 | 9.11E-06 |
| EGR1 | 97.78 | -1.71 | 2.52E-14 | 1.77E-13 |
| NR1D1 | 315.61 | -1.71 | 8.90E-39 | 2.26E-37 |
| PNRC1 | 616.89 | -1.72 | 4.38E-59 | 2.35E-57 |
| FAM114A1 | 1803.18 | -1.72 | 3.40E-137 | 7.11E-135 |
| GGT4P | 65.84 | -1.72 | 9.64E-11 | 5.17E-10 |
| SAT1 | 1007.68 | -1.72 | 1.88E-72 | 1.43E-70 |
| ISG20 | 61.82 | -1.72 | 2.12E-10 | 1.10E-09 |
| GLI1 | 34.32 | -1.72 | 3.75E-07 | 1.45E-06 |
| SLC2A12 | 50.48 | -1.72 | 8.85E-09 | 4.02E-08 |
| SELENOP | 6.58 | -1.74 | 1.11E-03 | 2.77E-03 |
| SULT1A1 | 1452.91 | -1.74 | 2.29E-119 | 3.82E-117 |
| PER1 | 197.41 | -1.75 | 7.73E-25 | 1.02E-23 |
| DENND2D | 6.05 | -1.75 | 1.27E-03 | 3.15E-03 |
| RBM43 | 138.84 | -1.75 | 9.83E-19 | 9.36E-18 |
| POLD4 | 236.89 | -1.76 | 2.92E-27 | 4.39E-26 |
| DPP4 | 786.06 | -1.76 | 8.62E-72 | 6.41E-70 |
| RASD2 | 40.10 | -1.76 | 1.13E-06 | 4.15E-06 |
| CD68 | 779.28 | -1.76 | 3.07E-83 | 2.83E-81 |
| RPL36A-HNRNPH2 | 376.70 | -1.76 | 4.33E-37 | 1.01E-35 |
| ERICH2 | 130.71 | -1.76 | 3.64E-19 | 3.57E-18 |
| TSHZ2 | 77.86 | -1.76 | 4.02E-11 | 2.23E-10 |
| CPLX3 | 3.39 | -1.76 | 1.32E-03 | 3.28E-03 |
| HSPB3 | 22.83 | -1.77 | 1.59E-05 | 5.15E-05 |
| CEACAMP10 | 304.37 | -1.78 | 9.88E-37 | 2.26E-35 |
| IQSEC2 | 444.30 | -1.78 | 2.37E-54 | 1.03E-52 |
| ANKRD24 | 43.06 | -1.79 | 6.90E-08 | 2.87E-07 |
| ARHGEF6 | 375.94 | -1.79 | 5.96E-39 | 1.53E-37 |
| ACSL1 | 1800.00 | -1.80 | 1.33E-143 | 3.00E-141 |
| SECTM1 | 203.10 | -1.80 | 3.74E-24 | 4.72E-23 |
| PPP2R2C | 17.78 | -1.80 | 3.93E-05 | 1.21E-04 |
| NUDT7 | 117.19 | -1.81 | 7.21E-17 | 6.06E-16 |
| NXPH4 | 461.74 | -1.82 | 3.85E-47 | 1.35E-45 |
| ESPN | 10.86 | -1.82 | 3.66E-04 | 9.86E-04 |
| GREB1 | 449.47 | -1.82 | 3.91E-56 | 1.85E-54 |
| SAMD9 | 586.68 | -1.82 | 2.09E-57 | 1.05E-55 |
| ACVR1C | 28.90 | -1.84 | 9.13E-07 | 3.38E-06 |
| PDZD7 | 34.05 | -1.84 | 1.88E-07 | 7.50E-07 |
| P2RX6 | 15.95 | -1.84 | 6.54E-05 | 1.96E-04 |
| TRIB3 | 2012.93 | -1.84 | 4.00E-109 | 5.74E-107 |
| EVI5L | 633.98 | -1.85 | 3.27E-73 | 2.53E-71 |
| ARRDC3 | 507.90 | -1.85 | 1.94E-59 | 1.05E-57 |
| FCHO1 | 86.36 | -1.85 | 1.82E-12 | 1.12E-11 |
| KLF9 | 498.07 | -1.85 | 7.31E-63 | 4.31E-61 |
| TMEM129 | 1532.25 | -1.86 | 1.84E-144 | 4.28E-142 |
| ACBD4 | 299.11 | -1.86 | 4.36E-36 | 9.65E-35 |
| TNFRSF9 | 529.61 | -1.87 | 1.37E-56 | 6.62E-55 |
| BTC | 30.44 | -1.87 | 1.05E-06 | 3.88E-06 |
| NFAM1 | 14.68 | -1.88 | 8.11E-05 | 2.39E-04 |
| EML2 | 2578.06 | -1.88 | 9.29E-210 | 4.15E-207 |
| RASSF4 | 215.92 | -1.89 | 2.64E-30 | 4.57E-29 |
| IL1R1 | 131.79 | -1.89 | 6.34E-21 | 6.73E-20 |
| ADGRA2 | 342.80 | -1.89 | 2.78E-45 | 9.16E-44 |
| INPP5J | 139.07 | -1.90 | 1.21E-18 | 1.15E-17 |
| TSPAN10 | 949.08 | -1.90 | 3.23E-80 | 2.77E-78 |
| SQOR | 465.44 | -1.90 | 5.81E-57 | 2.85E-55 |
| ANO9 | 57.26 | -1.90 | 3.53E-11 | 1.97E-10 |
| ZSCAN18 | 82.19 | -1.92 | 4.35E-14 | 3.00E-13 |
| NCF2 | 48.48 | -1.92 | 3.62E-09 | 1.70E-08 |
| SPRY1 | 28.80 | -1.93 | 9.18E-07 | 3.40E-06 |
| IFITM3 | 4301.86 | -1.93 | 7.12E-263 | 5.60E-260 |
| CCDC194 | 33.03 | -1.94 | 9.42E-08 | 3.86E-07 |
| MUC1 | 178.39 | -1.94 | 7.74E-26 | 1.07E-24 |
| SCNN1A | 72.27 | -1.94 | 3.69E-12 | 2.22E-11 |
| SLC25A18 | 191.49 | -1.95 | 2.49E-30 | 4.32E-29 |
| TPK1 | 288.70 | -1.96 | 1.19E-41 | 3.39E-40 |
| AKR1C3 | 55.69 | -1.96 | 3.47E-10 | 1.78E-09 |
| PLSCR1 | 1647.30 | -1.96 | 6.96E-179 | 2.17E-176 |
| IFIT5 | 806.61 | -1.96 | 1.03E-98 | 1.24E-96 |
| SNPH | 220.99 | -1.97 | 9.65E-34 | 1.94E-32 |
| CDK18 | 630.94 | -1.97 | 1.03E-81 | 9.28E-80 |
| FBXL9P | 62.44 | -1.97 | 2.47E-12 | 1.50E-11 |
| TRIM69 | 308.74 | -1.97 | 2.29E-37 | 5.44E-36 |
| NRN1 | 43.96 | -1.98 | 2.06E-09 | 9.85E-09 |
| NT5M | 75.85 | -1.98 | 5.18E-13 | 3.30E-12 |
| PDGFRL | 98.00 | -1.99 | 2.88E-17 | 2.47E-16 |
| ADRA1B | 107.21 | -1.99 | 8.15E-20 | 8.21E-19 |
| IL2RG | 126.91 | -2.00 | 2.03E-22 | 2.35E-21 |
| CTSS | 163.79 | -2.00 | 3.34E-26 | 4.74E-25 |
| TFEB | 84.73 | -2.00 | 6.62E-14 | 4.51E-13 |
| EGLN3 | 451.66 | -2.00 | 1.10E-56 | 5.34E-55 |
| SAMD13 | 17.51 | -2.01 | 1.46E-05 | 4.76E-05 |
| CCN5 | 251.26 | -2.01 | 2.49E-39 | 6.46E-38 |
| ARHGAP4 | 46.10 | -2.02 | 2.70E-09 | 1.28E-08 |
| SNTA1 | 1400.48 | -2.02 | 3.43E-126 | 6.52E-124 |
| BLVRB | 2085.83 | -2.02 | 1.93E-150 | 4.90E-148 |
| ALDH3B1 | 1516.74 | -2.02 | 4.06E-145 | 9.70E-143 |
| COL4A3 | 72.56 | -2.02 | 2.38E-13 | 1.56E-12 |
| ST6GALNAC2 | 10.39 | -2.02 | 1.73E-04 | 4.88E-04 |
| MTMR11 | 156.05 | -2.03 | 2.63E-23 | 3.18E-22 |
| TRANK1 | 521.95 | -2.03 | 7.71E-50 | 2.90E-48 |
| SRRM3 | 167.50 | -2.04 | 5.31E-29 | 8.72E-28 |
| PLAAT2 | 14.45 | -2.04 | 3.77E-05 | 1.16E-04 |
| IL4I1 | 55.60 | -2.04 | 6.80E-12 | 4.00E-11 |
| NRG2 | 103.09 | -2.04 | 1.20E-18 | 1.13E-17 |
| APOBEC3F | 218.17 | -2.05 | 1.54E-34 | 3.21E-33 |
| PCDH1 | 253.95 | -2.07 | 8.35E-41 | 2.31E-39 |
| FAXDC2 | 126.89 | -2.07 | 1.13E-22 | 1.33E-21 |
| CRAT | 1048.40 | -2.08 | 4.42E-135 | 9.12E-133 |
| SLC9A9 | 103.36 | -2.08 | 1.93E-20 | 2.00E-19 |
| STAT1 | 7137.06 | -2.09 | 0.00E+00 | 0.00E+00 |
| NKAIN4 | 26.15 | -2.09 | 2.73E-07 | 1.07E-06 |
| ABCD1 | 695.66 | -2.12 | 6.28E-103 | 8.11E-101 |
| COPZ2 | 34.01 | -2.13 | 4.95E-08 | 2.09E-07 |
| PDE2A | 16.68 | -2.13 | 1.17E-05 | 3.85E-05 |
| CLDN14 | 15.16 | -2.14 | 2.33E-05 | 7.36E-05 |
| FBXO32 | 642.20 | -2.14 | 2.99E-91 | 3.10E-89 |
| IRF9 | 235.54 | -2.15 | 8.55E-33 | 1.64E-31 |
| GPD1 | 17.69 | -2.16 | 1.93E-05 | 6.19E-05 |
| SPTLC3 | 74.05 | -2.16 | 6.00E-15 | 4.43E-14 |
| APOL1 | 1056.00 | -2.16 | 3.44E-117 | 5.56E-115 |
| TJP3 | 161.34 | -2.17 | 1.57E-30 | 2.76E-29 |
| S100A14 | 12.41 | -2.17 | 9.18E-05 | 2.69E-04 |
| NGEF | 633.29 | -2.18 | 8.29E-93 | 8.83E-91 |
| PFKFB4 | 435.44 | -2.18 | 2.09E-69 | 1.51E-67 |
| NKAIN1 | 5.69 | -2.18 | 5.81E-04 | 1.52E-03 |
| SESN3 | 223.91 | -2.18 | 1.39E-34 | 2.92E-33 |
| RDH12 | 10.91 | -2.18 | 9.13E-05 | 2.68E-04 |
| RIGI | 2063.22 | -2.18 | 1.03E-215 | 5.51E-213 |
| SLPI | 11.87 | -2.18 | 6.85E-05 | 2.04E-04 |
| HLA-F | 403.92 | -2.19 | 3.98E-38 | 9.73E-37 |
| DEPTOR | 77.34 | -2.19 | 2.00E-15 | 1.54E-14 |
| PPP1R3C | 416.42 | -2.19 | 1.31E-62 | 7.70E-61 |
| CHCHD10 | 1453.11 | -2.21 | 4.57E-129 | 8.98E-127 |
| ITGAX | 607.89 | -2.22 | 2.79E-94 | 3.14E-92 |
| SAMHD1 | 1794.05 | -2.22 | 3.44E-187 | 1.24E-184 |
| SRCIN1 | 12.66 | -2.22 | 7.76E-05 | 2.30E-04 |
| EFNA4-EFNA3 | 4.05 | -2.24 | 5.50E-04 | 1.44E-03 |
| METTL7A | 352.96 | -2.24 | 1.84E-58 | 9.75E-57 |
| FAM131C | 45.51 | -2.25 | 1.10E-10 | 5.90E-10 |
| IFIT2 | 1506.87 | -2.25 | 4.04E-196 | 1.63E-193 |
| UBE2L6 | 1642.89 | -2.25 | 1.24E-212 | 5.86E-210 |
| ALDH1L1 | 305.20 | -2.25 | 8.07E-58 | 4.14E-56 |
| GPT | 39.13 | -2.25 | 6.95E-10 | 3.47E-09 |
| FCGR2A | 8.28 | -2.25 | 3.16E-04 | 8.59E-04 |
| PLD1 | 888.71 | -2.26 | 2.06E-115 | 3.25E-113 |
| APOL6 | 1616.29 | -2.26 | 9.11E-185 | 3.13E-182 |
| HERC5 | 1771.31 | -2.27 | 1.13E-193 | 4.35E-191 |
| RAB26 | 370.02 | -2.27 | 2.36E-57 | 1.18E-55 |
| SARDH | 1513.79 | -2.27 | 3.09E-145 | 7.50E-143 |
| GPIHBP1 | 14.27 | -2.28 | 2.45E-05 | 7.73E-05 |
| ADHFE1 | 59.35 | -2.28 | 2.39E-13 | 1.57E-12 |
| MYO3B | 14.49 | -2.28 | 2.54E-05 | 7.99E-05 |
| TTC39A | 448.75 | -2.29 | 1.23E-74 | 9.73E-73 |
| GBP4 | 153.23 | -2.29 | 1.02E-31 | 1.87E-30 |
| CAPG | 1727.90 | -2.30 | 1.80E-182 | 5.95E-180 |
| VEPH1 | 164.33 | -2.31 | 6.67E-35 | 1.41E-33 |
| SNCG | 967.74 | -2.32 | 3.79E-93 | 4.06E-91 |
| SLC15A3 | 194.77 | -2.32 | 4.87E-41 | 1.37E-39 |
| FER1L4 | 65.42 | -2.32 | 3.11E-13 | 2.01E-12 |
| TAP1 | 2714.68 | -2.33 | 1.21E-214 | 6.04E-212 |
| ASS1 | 1590.12 | -2.35 | 1.00E-214 | 5.18E-212 |
| CFB | 72.82 | -2.35 | 1.54E-14 | 1.10E-13 |
| ACSS2 | 2689.90 | -2.36 | 3.24E-289 | 3.56E-286 |
| ITGB4 | 168.84 | -2.36 | 1.79E-36 | 4.06E-35 |
| INHA | 17.95 | -2.36 | 5.80E-06 | 1.98E-05 |
| ERVV-2 | 546.23 | -2.37 | 7.72E-99 | 9.37E-97 |
| USP18 | 655.87 | -2.38 | 5.54E-121 | 9.62E-119 |
| MTMR9LP | 28.87 | -2.38 | 9.29E-09 | 4.21E-08 |
| KCNE4 | 478.94 | -2.38 | 1.18E-81 | 1.05E-79 |
| PARP10 | 2405.66 | -2.39 | 3.85E-144 | 8.82E-142 |
| SLC12A8 | 348.84 | -2.39 | 1.60E-57 | 8.13E-56 |
| ENDOD1 | 382.84 | -2.40 | 2.65E-74 | 2.10E-72 |
| ALDOC | 173.33 | -2.40 | 3.29E-35 | 7.06E-34 |
| S1PR4 | 13.63 | -2.41 | 1.30E-05 | 4.24E-05 |
| PIP5KL1 | 209.62 | -2.42 | 1.41E-43 | 4.36E-42 |
| SH3TC1 | 281.62 | -2.43 | 6.64E-58 | 3.42E-56 |
| INSIG1 | 4540.33 | -2.43 | 1.29E-286 | 1.33E-283 |
| GDF15 | 580.92 | -2.47 | 8.31E-103 | 1.06E-100 |
| MLXIPL | 226.67 | -2.48 | 4.74E-42 | 1.38E-40 |
| PDZK1IP1 | 81.40 | -2.48 | 1.55E-20 | 1.61E-19 |
| METTL27 | 96.05 | -2.48 | 9.55E-24 | 1.18E-22 |
| MMRN2 | 128.38 | -2.48 | 1.19E-25 | 1.62E-24 |
| LTF | 64.62 | -2.49 | 3.02E-15 | 2.28E-14 |
| GBP2 | 888.07 | -2.50 | 7.18E-120 | 1.24E-117 |
| SMIM14 | 738.17 | -2.50 | 1.11E-125 | 2.04E-123 |
| DBP | 332.42 | -2.50 | 5.04E-55 | 2.25E-53 |
| TSC22D3 | 617.87 | -2.52 | 7.47E-115 | 1.16E-112 |
| KCNN1 | 22.20 | -2.53 | 4.20E-07 | 1.62E-06 |
| UNC13A | 56.99 | -2.53 | 1.10E-14 | 7.92E-14 |
| CA11 | 204.79 | -2.57 | 1.19E-41 | 3.39E-40 |
| FA2H | 108.85 | -2.58 | 8.74E-27 | 1.28E-25 |
| ANXA9 | 198.41 | -2.58 | 2.37E-43 | 7.27E-42 |
| TLE2 | 58.96 | -2.59 | 9.20E-16 | 7.20E-15 |
| INHBE | 296.08 | -2.61 | 1.23E-57 | 6.29E-56 |
| SDSL | 187.79 | -2.61 | 6.08E-38 | 1.47E-36 |
| GCNT3 | 175.47 | -2.62 | 9.93E-37 | 2.26E-35 |
| ORAI3 | 499.02 | -2.62 | 5.81E-102 | 7.27E-100 |
| SPNS3 | 42.88 | -2.64 | 2.60E-11 | 1.46E-10 |
| PLIN4 | 77.92 | -2.66 | 1.56E-19 | 1.56E-18 |
| SNAP91 | 52.98 | -2.66 | 3.01E-15 | 2.27E-14 |
| CDK5R2 | 39.46 | -2.66 | 7.87E-12 | 4.61E-11 |
| CCM2L | 46.17 | -2.66 | 7.77E-14 | 5.27E-13 |
| IFI16 | 1158.87 | -2.67 | 4.98E-184 | 1.68E-181 |
| EXOC3L4 | 23.40 | -2.67 | 5.85E-08 | 2.45E-07 |
| SPAG4 | 189.29 | -2.68 | 4.40E-40 | 1.18E-38 |
| TRIM22 | 104.97 | -2.69 | 3.72E-26 | 5.26E-25 |
| ODF3B | 56.98 | -2.71 | 3.08E-16 | 2.49E-15 |
| UPB1 | 74.48 | -2.72 | 1.24E-20 | 1.29E-19 |
| PARP9 | 1534.14 | -2.73 | 1.27E-217 | 7.23E-215 |
| ADGRG1 | 996.53 | -2.75 | 4.00E-180 | 1.27E-177 |
| HERC6 | 578.41 | -2.76 | 3.53E-105 | 4.70E-103 |
| OAS3 | 4818.07 | -2.77 | 2.45E-297 | 2.88E-294 |
| SEC14L5 | 9.68 | -2.80 | 6.82E-05 | 2.03E-04 |
| FUT1 | 66.90 | -2.81 | 1.19E-18 | 1.13E-17 |
| C1S | 396.58 | -2.81 | 1.80E-94 | 2.04E-92 |
| SLC2A5 | 79.74 | -2.84 | 1.08E-22 | 1.27E-21 |
| HYAL1 | 108.39 | -2.86 | 1.82E-25 | 2.46E-24 |
| C3 | 269.75 | -2.87 | 1.92E-72 | 1.45E-70 |
| MAT1A | 14.21 | -2.88 | 2.65E-06 | 9.37E-06 |
| NOXA1 | 26.57 | -2.89 | 6.94E-09 | 3.18E-08 |
| CLIC3 | 658.40 | -2.90 | 1.41E-119 | 2.40E-117 |
| CT47A12 | 4.48 | -2.91 | 1.69E-04 | 4.78E-04 |
| BLACAT1 | 37.04 | -2.91 | 1.21E-12 | 7.53E-12 |
| IFIT3 | 2324.11 | -2.93 | 0.00E+00 | 0.00E+00 |
| TLR1 | 26.89 | -2.94 | 1.39E-09 | 6.74E-09 |
| SH2D3C | 37.63 | -2.95 | 5.46E-13 | 3.47E-12 |
| PALMD | 64.45 | -2.96 | 4.63E-20 | 4.71E-19 |
| THEMIS2 | 76.12 | -2.97 | 2.02E-23 | 2.46E-22 |
| BHLHE40 | 1837.90 | -2.97 | 0.00E+00 | 0.00E+00 |
| APOL3 | 111.73 | -2.97 | 2.25E-33 | 4.42E-32 |
| DUSP13 | 88.79 | -2.99 | 1.89E-26 | 2.71E-25 |
| CCR10 | 113.76 | -3.02 | 2.34E-32 | 4.44E-31 |
| CPEB1 | 35.26 | -3.03 | 3.93E-12 | 2.36E-11 |
| ZNF467 | 176.89 | -3.04 | 1.23E-45 | 4.15E-44 |
| CCR3 | 15.50 | -3.05 | 9.90E-07 | 3.66E-06 |
| MB | 45.98 | -3.05 | 9.83E-16 | 7.67E-15 |
| IFIH1 | 1816.69 | -3.06 | 0.00E+00 | 0.00E+00 |
| SNAI3 | 25.86 | -3.07 | 1.68E-09 | 8.10E-09 |
| ANGPTL4 | 62.16 | -3.07 | 2.76E-18 | 2.55E-17 |
| TRIM34 | 77.89 | -3.07 | 4.96E-20 | 5.04E-19 |
| TRPM8 | 30.49 | -3.09 | 3.23E-11 | 1.81E-10 |
| TSPAN1 | 98.40 | -3.10 | 2.47E-28 | 3.92E-27 |
| CCL5 | 880.86 | -3.11 | 1.93E-203 | 7.95E-201 |
| RAB7B | 38.98 | -3.13 | 2.00E-13 | 1.32E-12 |
| BATF2 | 83.29 | -3.14 | 5.67E-26 | 7.88E-25 |
| SLC22A18 | 509.28 | -3.16 | 2.26E-127 | 4.39E-125 |
| PLAAT4 | 69.92 | -3.16 | 1.88E-22 | 2.19E-21 |
| IFI27 | 454.97 | -3.17 | 1.00E-83 | 9.28E-82 |
| CASP1 | 45.21 | -3.18 | 3.98E-15 | 2.97E-14 |
| TXNIP | 921.69 | -3.20 | 5.43E-210 | 2.49E-207 |
| ABCA1 | 368.85 | -3.21 | 1.64E-94 | 1.87E-92 |
| BIRC7 | 931.08 | -3.23 | 1.14E-49 | 4.24E-48 |
| TP73 | 13.64 | -3.23 | 1.73E-06 | 6.26E-06 |
| DDIT4 | 1349.22 | -3.24 | 6.34E-233 | 4.03E-230 |
| KLHDC7B | 23.55 | -3.26 | 1.06E-09 | 5.22E-09 |
| HELZ2 | 5739.57 | -3.27 | 0.00E+00 | 0.00E+00 |
| DEPP1 | 73.65 | -3.28 | 6.79E-23 | 8.08E-22 |
| FER1L6 | 4.99 | -3.31 | 8.30E-05 | 2.45E-04 |
| FAM107A | 32.92 | -3.32 | 8.83E-13 | 5.55E-12 |
| FOS | 79.75 | -3.35 | 1.15E-27 | 1.77E-26 |
| CA9 | 123.06 | -3.37 | 1.11E-34 | 2.33E-33 |
| DHRS3 | 624.93 | -3.39 | 7.75E-153 | 2.03E-150 |
| PLCD4 | 68.34 | -3.40 | 6.27E-21 | 6.67E-20 |
| IL1B | 67.58 | -3.40 | 5.02E-24 | 6.28E-23 |
| OASL | 463.57 | -3.43 | 1.65E-144 | 3.89E-142 |
| IFI35 | 1019.23 | -3.44 | 4.39E-240 | 2.90E-237 |
| NFE2 | 15.14 | -3.46 | 4.20E-07 | 1.62E-06 |
| DHX58 | 108.42 | -3.46 | 1.84E-37 | 4.38E-36 |
| PSG5 | 202.72 | -3.54 | 1.89E-65 | 1.20E-63 |
| C15orf48 | 124.57 | -3.54 | 3.47E-43 | 1.06E-41 |
| ISG15 | 5875.47 | -3.56 | 0.00E+00 | 0.00E+00 |
| EPSTI1 | 1025.13 | -3.57 | 1.99E-249 | 1.43E-246 |
| PSMB9 | 39.45 | -3.60 | 2.04E-14 | 1.45E-13 |
| FBXO39 | 8.57 | -3.62 | 3.38E-05 | 1.05E-04 |
| ACHE | 33.17 | -3.63 | 1.19E-13 | 7.99E-13 |
| MAF | 178.32 | -3.64 | 3.97E-56 | 1.88E-54 |
| PIK3IP1 | 49.35 | -3.67 | 6.64E-19 | 6.40E-18 |
| CFI | 46.56 | -3.69 | 1.15E-15 | 8.96E-15 |
| DDX60 | 995.18 | -3.73 | 1.73E-246 | 1.19E-243 |
| HSH2D | 11.74 | -3.73 | 1.02E-05 | 3.39E-05 |
| GAS2L2 | 9.47 | -3.89 | 1.90E-05 | 6.10E-05 |
| IFI44 | 667.07 | -3.90 | 3.03E-206 | 1.32E-203 |
| IFIT1 | 2447.56 | -4.04 | 0.00E+00 | 0.00E+00 |
| SAMD9L | 558.90 | -4.04 | 3.02E-169 | 8.91E-167 |
| STC1 | 196.59 | -4.05 | 2.08E-69 | 1.51E-67 |
| UBA7 | 263.96 | -4.05 | 1.71E-93 | 1.87E-91 |
| CXCL11 | 68.93 | -4.29 | 1.66E-27 | 2.54E-26 |
| OAS1 | 1357.60 | -4.32 | 0.00E+00 | 0.00E+00 |
| PLA2G3 | 24.11 | -4.35 | 1.73E-10 | 9.08E-10 |
| CXCL10 | 47.91 | -4.36 | 1.99E-19 | 1.98E-18 |
| IFI6 | 4887.38 | -4.41 | 0.00E+00 | 0.00E+00 |
| CMPK2 | 172.51 | -4.47 | 1.75E-64 | 1.07E-62 |
| MX2 | 182.30 | -4.52 | 1.89E-68 | 1.34E-66 |
| RSAD2 | 367.76 | -4.57 | 2.29E-119 | 3.82E-117 |
| FGF21 | 38.65 | -4.75 | 1.43E-15 | 1.11E-14 |
| TNFSF10 | 13.04 | -4.77 | 1.80E-06 | 6.50E-06 |
| PLEK | 8.87 | -5.12 | 1.25E-06 | 4.59E-06 |
| MX1 | 6468.04 | -5.17 | 0.00E+00 | 0.00E+00 |
| CCR1 | 53.92 | -5.50 | 5.86E-19 | 5.66E-18 |
| IFITM1 | 675.02 | -5.61 | 2.57E-205 | 1.09E-202 |
| SDS | 397.81 | -5.73 | 7.17E-126 | 1.34E-123 |
| IL21R | 156.59 | -6.11 | 6.28E-47 | 2.18E-45 |
| VCAM1 | 48.87 | -6.16 | 3.96E-14 | 2.75E-13 |
| OAS2 | 2080.64 | -6.21 | 0.00E+00 | 0.00E+00 |
| NDUFA4L2 | 121.02 | -6.21 | 8.06E-34 | 1.63E-32 |
| XAF1 | 1006.72 | -6.30 | 3.27E-271 | 2.84E-268 |
| RORC | 66.42 | -6.32 | 1.66E-18 | 1.55E-17 |
| LDHD | 166.34 | -6.42 | 2.06E-45 | 6.85E-44 |
| RDM1 | 17.49 | -6.55 | 2.05E-09 | 9.82E-09 |
| IFI44L | 813.51 | -7.73 | 5.16E-138 | 1.09E-135 |
| TMEM265 | 42.93 | -7.94 | 3.80E-13 | 2.44E-12 |
| IFI27L1 | 46.38 | -8.06 | 1.37E-13 | 9.11E-13 |
| TFPT | 69.11 | -8.64 | 2.01E-15 | 1.54E-14 |
| AK6 | 72.96 | -8.71 | 1.51E-15 | 1.17E-14 |
| FLOT1 | 466.53 | -10.38 | 2.60E-13 | 1.70E-12 |

**Table S2**. List of the top 100 SOX12-binding genes in Huh7-SOX12-Flag cell calculated from the ChIP-seq data

| Gene | SOX12-Flag score | annotation |
| --- | --- | --- |
| EEF1A1 | 196 | Promoter (<=1kb) |
| MTF2 | 189 | Promoter (<=1kb) |
| CD274 | 186 | Promoter (<=1kb) |
| SOX12 | 179 | Promoter (<=1kb) |
| ZNF3 | 141 | Promoter (<=1kb) |
| CCL22 | 130 | Promoter (<=1kb) |
| MC5R | 127 | Promoter (<=1kb) |
| H4C5 | 126 | Promoter (<=1kb) |
| TWIST1 | 118 | Promoter (<=1kb) |
| CCN1 | 114 | Promoter (<=1kb) |
| KANSL3 | 112 | Promoter (<=1kb) |
| PRCC | 108 | Promoter (<=1kb) |
| FGFBP1 | 108 | Promoter (<=1kb) |
| RPS10 | 108 | Promoter (<=1kb) |
| LOC124905574 | 108 | Promoter (<=1kb) |
| EGR1 | 107 | Promoter (<=1kb) |
| GNAS | 107 | Promoter (<=1kb) |
| LINC02009 | 105 | Promoter (<=1kb) |
| LOC124902309 | 104 | Promoter (<=1kb) |
| RAB5IF | 104 | Promoter (<=1kb) |
| FLJ39095 | 102 | Promoter (<=1kb) |
| MKNK2 | 102 | Promoter (<=1kb) |
| NANP | 101 | Promoter (<=1kb) |
| IGF1 | 101 | Promoter (<=1kb) |
| LINC00526 | 100 | Promoter (<=1kb) |
| CALM2 | 98 | Promoter (<=1kb) |
| LINC00526 | 97 | Promoter (<=1kb) |
| SETD2 | 97 | Promoter (<=1kb) |
| PPIA | 96 | Promoter (<=1kb) |
| HERPUD1 | 96 | Promoter (<=1kb) |
| DYNC2I2 | 93 | Promoter (<=1kb) |
| KLF12 | 93 | Promoter (<=1kb) |
| MMP7 | 93 | Promoter (<=1kb) |
| PLEKHG2 | 93 | Promoter (<=1kb) |
| TSC1 | 91 | Promoter (<=1kb) |
| GOT2 | 88 | Promoter (<=1kb) |
| MIR17HG | 88 | Promoter (<=1kb) |
| YWHAB | 88 | Promoter (<=1kb) |
| TNNC1 | 87 | Promoter (<=1kb) |
| RMDN3 | 87 | Promoter (<=1kb) |
| FOSB | 87 | Promoter (<=1kb) |
| DOCK7 | 86 | Promoter (<=1kb) |
| LOC101928237 | 86 | Promoter (<=1kb) |
| ARRB1 | 86 | Promoter (<=1kb) |
| ENKD1 | 86 | Promoter (<=1kb) |
| PIK3R3 | 85 | Promoter (<=1kb) |
| H2AC17 | 85 | Promoter (<=1kb) |
| TNFRSF12A | 85 | Promoter (<=1kb) |
| HES4 | 84 | Promoter (<=1kb) |
| SDCBP | 83 | Promoter (<=1kb) |
| SMARCD2 | 83 | Promoter (<=1kb) |
| MIDN | 83 | Promoter (<=1kb) |
| GNB1 | 82 | Promoter (<=1kb) |
| ASNS | 82 | Promoter (<=1kb) |
| UGGT1 | 82 | Promoter (<=1kb) |
| RB1CC1 | 82 | Promoter (<=1kb) |
| HCFC1 | 82 | Promoter (<=1kb) |
| MAST2 | 81 | Promoter (<=1kb) |
| MYL5 | 81 | Promoter (<=1kb) |
| OGFRL1 | 81 | Promoter (<=1kb) |
| NEAT1 | 81 | Promoter (<=1kb) |
| HSP90AA1 | 81 | Promoter (<=1kb) |
| GLS | 81 | Promoter (<=1kb) |
| PIAS4 | 81 | Promoter (<=1kb) |
| XRN2 | 81 | Promoter (<=1kb) |
| C16orf87 | 80 | Promoter (<=1kb) |
| HSBP1 | 79 | Promoter (<=1kb) |
| RPS15 | 78 | Promoter (<=1kb) |
| RNF19B | 77 | Promoter (<=1kb) |
| GCLM | 77 | Promoter (<=1kb) |
| PTBP2 | 77 | Promoter (<=1kb) |
| IDO1 | 77 | Promoter (<=1kb) |
| CAMK2N2 | 77 | Promoter (<=1kb) |
| FNIP2 | 77 | Promoter (<=1kb) |
| HMGCS1 | 77 | Promoter (<=1kb) |
| BEND3 | 77 | Promoter (<=1kb) |
| ZBTB24-DT | 77 | Promoter (<=1kb) |
| PSMD5 | 77 | Promoter (<=1kb) |
| GSN | 77 | Promoter (<=1kb) |
| GARNL3 | 77 | Promoter (<=1kb) |
| KMT5B | 77 | Promoter (<=1kb) |
| UHRF1BP1L | 77 | Promoter (<=1kb) |
| LATS2 | 77 | Promoter (<=1kb) |
| FGF9 | 77 | Promoter (<=1kb) |
| POLG | 77 | Promoter (<=1kb) |
| FCSK | 77 | Promoter (<=1kb) |
| CCL5 | 77 | Promoter (<=1kb) |
| PVR | 77 | Promoter (<=1kb) |
| CDK5RAP1 | 77 | Promoter (<=1kb) |
| ADARB1 | 77 | Promoter (<=1kb) |
| SUMO1 | 76 | Promoter (<=1kb) |
| FAM184B | 76 | Promoter (<=1kb) |
| SPCS2P3 | 76 | Promoter (<=1kb) |
| TALAM1 | 76 | Promoter (<=1kb) |
| RCE1 | 76 | Promoter (<=1kb) |
| POGLUT3 | 76 | Promoter (<=1kb) |
| MZT1 | 76 | Promoter (<=1kb) |
| STMN1 | 75 | Promoter (<=1kb) |
| SNHG12 | 75 | Promoter (<=1kb) |

Score：10*(-log10(qvalue)).

**Table S3**. Correlation between CCL22 expression and clinicopathological characteristics of HCC patients

|  |  | Cohort I | | |  |  | Cohort II | | |  |
| --- | --- | --- | --- | --- | --- | --- | --- | --- | --- | --- |
| Clinicopathological variables | | Tumor CCL22 expression | | | *P* Value |  | Tumor CCL22 expression | | | *P* Value |
|  |  | Negative (n=162) | | Positive (n=98) |  |  | Negative (n=169) | | Positive (n=111) |  |
| Age | | 52.53(10.420) | | 51.03(12.601) | 0.179 |  | | 50.43(9.907) | 51.85(9.894) | 0.842 |
| Sex | female | | 27 | 22 | 0.256 |  | | 24 | 20 | 0.405 |
|  | male | | 135 | 76 |  |  | | 145 | 91 |  |
| Serum AFP | ≤20ng/ml | | 46 | 21 | 0.243 |  | | 29 | 26 | 0.220 |
|  | >20ng/ml | | 116 | 77 |  |  | | 140 | 85 |  |
| Virus infection | HBV | | 125 | 67 | 0.307 |  | | 134 | 91 | 0.942 |
|  | HCV | | 12 | 13 |  |  | | 12 | 7 |  |
|  | HBV+HCV | | 6 | 6 |  |  | | 10 | 5 |  |
|  | none | | 19 | 12 |  |  | | 13 | 8 |  |
| Cirrrhosis | absent | | 46 | 26 | 0.777 |  | | 48 | 28 | 0.585 |
|  | present | | 116 | 72 |  |  | | 121 | 83 |  |
| Child-pugh score | Class A | | 124 | 72 | 0.656 |  | | 143 | 90 | 0.514 |
|  | Class B | | 38 | 26 |  |  | | 26 | 21 |  |
| Tumor number | single | | 109 | 45 | 0.001 |  | | 135 | 75 | 0.024 |
|  | multiple | | 53 | 53 |  |  | | 34 | 36 |  |
| Maximal tumor size | ≤5cm | | 78 | 30 | 0.006 |  | | 120 | 38 | <0.001 |
|  | >5cm | | 84 | 68 |  |  | | 49 | 73 |  |
| Tumor encapsulation | absent | | 113 | 32 | <0.001 |  | | 131 | 64 | 0.001 |
|  | present | | 49 | 66 |  |  | | 38 | 47 |  |
| Microvascular invasion | absent | | 108 | 34 | <0.001 |  | | 112 | 41 | <0.001 |
|  | present | | 54 | 64 |  |  | 57 | | 70 |  |
| Tumor differentiation | I-II | | 141 | 65 | <0.001 |  | 143 | | 66 | <0.001 |
|  | III-Ⅳ | | 21 | 33 |  |  | 26 | | 45 |  |
| TNM stage | I-II | | 146 | 52 | <0.001 |  | 145 | | 73 | <0.001 |
|  | III-Ⅳ | 16 | | 46 |  |  | 24 | | 38 |  |

**Table S4**. Correlation between PD-L1 expression and clinicopathological characteristics of HCC patients

|  |  | Cohort I | | |  |  | Cohort II | | |  |  |
| --- | --- | --- | --- | --- | --- | --- | --- | --- | --- | --- | --- |
| Clinicopathological variables | | Tumor PD-L1 expression | | | *P* Value |  | Tumor PD-L1 expression | | | *P* Value |  |
|  |  | Negative (n=213) | | Positive (n=47) |  |  | Negative (n=226) | | Positive (n=54) |  |  |
| Age | | | 52.13(11.505) | | 51.23(10.355) | 0.405 |  | | 51.20(9.815) | 50.13(10.340) | 0.840 |
| Sex | | female | | 36 | 13 | 0.100 |  | | 39 | 5 | 0.210 |
|  | | male | | 177 | 34 |  |  | | 187 | 49 |  |
| Serum AFP | | ≤20ng/ml | | 60 | 7 | 0.066 |  | | 46 | 9 | 0.703 |
|  | | >20ng/ml | | 153 | 40 |  |  | | 180 | 45 |  |
| Virus infection | | HBV | | 161 | 31 | 0.460 |  | | 174 | 51 | 0.027 |
|  | | HCV | | 18 | 7 |  |  | | 17 | 2 |  |
|  | | HBV+HCV | | 10 | 2 |  |  | | 14 | 1 |  |
|  | | none | | 24 | 7 |  |  | | 21 | 0 |  |
| Cirrrhosis | | absent | | 59 | 13 | 1.000 |  | | 67 | 9 | 0.061 |
|  | | present | | 154 | 34 |  |  | | 159 | 45 |  |
| Child-pugh score | | Class A | | 159 | 37 | 0.709 |  | | 187 | 46 | 0.840 |
|  | | Class B | | 54 | 10 |  |  | | 39 | 8 |  |
| Tumor number | | single | | 134 | 20 | 0.014 |  | | 182 | 28 | <0.001 |
|  | | multiple | | 79 | 27 |  |  | | 44 | 26 |  |
| Maximal tumor size | | ≤5cm | | 88 | 20 | 0.872 |  | | 118 | 40 | 0.004 |
|  | | >5cm | | 125 | 27 |  |  | | 108 | 14 |  |
| Tumor encapsulation | | absent | | 132 | 13 | <0.001 |  | | 168 | 27 | 0.001 |
|  | | present | | 81 | 34 |  |  | | 58 | 27 |  |
| Microvascular invasion | | absent | | 129 | 13 | <0.001 |  | | 131 | 22 | 0.033 |
|  | | present | | 84 | 34 |  |  | 95 | | 32 |  |
| Tumor differentiation | | I-II | | 177 | 29 | 0.002 |  | 181 | | 28 | <0.001 |
|  | | III-Ⅳ | | 36 | 18 |  |  | 45 | | 26 |  |
| TNM stage | | I-II | | 171 | 27 | 0.002 |  | 189 | | 29 | <0.001 |
|  | | III-Ⅳ | | 42 | 20 |  |  | 37 | | 25 |  |

**Table S5**. Correlation between SOX12 expression and clinicopathological characteristics of HCC patients

|  |  | Cohort I | |  |  | Cohort II | | |  |
| --- | --- | --- | --- | --- | --- | --- | --- | --- | --- |
| Clinicopathological variables | | Tumor SOX12 expression | | *P* Value |  | Tumor SOX12 expression | | | *P* Value |
|  |  | Negative (n=121) | Positive (n=139) |  |  | Negative (n=116) | | Positive (n=164) |  |
| Age | | 52.40(10.984) | 51.59(11.579) | 0.570 |  | | 51.65(10.108) | 50.53(9.769) | 0.299 |
| Sex | female | 24 | 25 | 0.752 |  | | 13 | 31 | 0.096 |
|  | male | 97 | 114 |  |  | | 103 | 133 |  |
| Serum AFP | ≤20ng/ml | 36 | 31 | 0.201 |  | | 29 | 26 | 0.067 |
|  | >20ng/ml | 85 | 108 |  |  | | 87 | 138 |  |
| Virus infection | HBV | 94 | 98 | 0.389 |  | | 97 | 128 | 0.131 |
|  | HCV | 11 | 14 |  |  | | 7 | 12 |  |
|  | HBV+HCV | 3 | 9 |  |  | | 8 | 7 |  |
|  | none | 13 | 18 |  |  | | 4 | 17 |  |
| Cirrrhosis | absent | 33 | 39 | 1.000 |  | | 30 | 46 | 0.785 |
|  | present | 88 | 100 |  |  | | 86 | 118 |  |
| Child-pugh score | Class A | 89 | 107 | 0.565 |  | | 102 | 131 | 0.104 |
|  | Class B | 32 | 32 |  |  | | 14 | 33 |  |
| Tumor number | single | 87 | 67 | <0.001 |  | | 93 | 117 | 0.123 |
|  | multiple | 34 | 72 |  |  | | 23 | 47 |  |
| Maximal tumor size | ≤5cm | 56 | 52 | 0.166 |  | | 72 | 86 | 0.114 |
|  | >5cm | 65 | 87 |  |  | | 44 | 78 |  |
| Tumor encapsulation | absent | 90 | 55 | <0.001 |  | | 93 | 102 | 0.001 |
|  | present | 31 | 84 |  |  | | 23 | 62 |  |
| Microvascular invasion | absent | 87 | 55 | <0.001 |  | | 72 | 81 | 0.039 |
|  | present | 34 | 84 |  |  | 44 | | 83 |  |
| Tumor differentiation | I-II | 107 | 99 | 0.001 |  | 99 | | 110 | <0.001 |
|  | III-Ⅳ | 14 | 40 |  |  | 17 | | 54 |  |
| TNM stage | I-II | 114 | 84 | <0.001 |  | 104 | | 114 | <0.001 |
|  | III-Ⅳ | 7 | 55 |  |  | 12 | | 50 |  |

**Table S6**. Correlation between Foxp3 expression and clinicopathological characteristics of HCC patients

|  | |  | Cohort I | |  |  | Cohort II | | |  |
| --- | --- | --- | --- | --- | --- | --- | --- | --- | --- | --- |
| Clinicopathological variables | | | Tumor Foxp3 expression | | *P* Value |  | Tumor Foxp3 expression | | | *P* Value |
|  |  |  | Negative (n=180) | Positive (n=80) |  |  | Negative (n=194) | | Positive (n=86) |  |
| Age | | | 52.52(10.979) | 50.73(11.944) | 0.407 |  | | 50.52(10.036) | 52.07(9.583) | 0.323 |
| Sex | female | | 29 | 20 | 0.121 |  | | 31 | 13 | 1.000 |
|  | male | | 151 | 60 |  |  | | 163 | 73 |  |
| Serum AFP | ≤20ng/ml | | 53 | 14 | 0.046 |  | | 46 | 9 | 0.009 |
|  | >20ng/ml | | 127 | 66 |  |  | | 148 | 77 |  |
| Virus infection | HBV | | 146 | 46 | 0.001 |  | | 155 | 70 | 0.484 |
|  | HCV | | 13 | 12 |  |  | | 11 | 8 |  |
|  | HBV+HCV | | 5 | 7 |  |  | | 12 | 3 |  |
|  | none | | 16 | 15 |  |  | | 16 | 5 |  |
| Cirrrhosis | absent | | 49 | 23 | 0.881 |  | | 53 | 23 | 1.000 |
|  | present | | 131 | 57 |  |  | | 141 | 63 |  |
| Child-pugh score | Class A | | 135 | 61 | 0.877 |  | | 162 | 71 | 0.863 |
|  | Class B | | 45 | 19 |  |  | | 32 | 15 |  |
| Tumor number | single | | 112 | 42 | 0.171 |  | | 152 | 58 | 0.072 |
|  | multiple | | 68 | 38 |  |  | | 42 | 28 |  |
| Maximal tumor size | ≤5cm | | 88 | 20 | <0.001 |  | | 114 | 44 | 0.243 |
|  | >5cm | | 92 | 60 |  |  | | 80 | 42 |  |
| Tumor encapsulation | absent | | 127 | 18 | <0.001 |  | | 149 | 46 | <0.001 |
|  | present | | 53 | 62 |  |  | | 45 | 40 |  |
| Microvascular invasion | absent | | 124 | 18 | <0.001 |  | | 119 | 34 | 0.001 |
|  | present | | 56 | 62 |  |  | 75 | | 52 |  |
| Tumor differentiation | I-II | | 149 | 57 | 0.046 |  | 157 | | 52 | 0.001 |
|  | III-Ⅳ | | 31 | 23 |  |  | 37 | | 34 |  |
| TNM stage | I-II | | 150 | 48 | <0.001 |  | 159 | | 59 | 0.019 |
|  | III-Ⅳ | | 30 | 32 |  |  | 35 | | 27 |  |

**Table S7**. Correlation between CD11b expression and clinicopathological characteristics of HCC patients

|  |  | Cohort I | | |  |  | Cohort II | | |  |
| --- | --- | --- | --- | --- | --- | --- | --- | --- | --- | --- |
| Clinicopathological variables | | Tumor CD11b expression | | | *P* Value |  | Tumor CD11b expression | | | *P* Value |
|  |  | Negative (n=191) | | Positive (n=69) |  |  | Negative (n=202) | | Positive (n=78) |  |
| Age | | 52.31 (11.116) | | 51.01(11.794) | 0.772 |  | | 50.83(10.194) | 51.42(9.176) | 0.203 |
| Sex | female | | 36 | 13 | 1.000 |  | | 30 | 14 | 0.583 |
|  | male | | 155 | 56 |  |  | | 172 | 64 |  |
| Serum AFP | ≤20ng/ml | | 56 | 11 | 0.036 |  | | 36 | 19 | 0.241 |
|  | >20ng/ml | | 135 | 58 |  |  | | 166 | 59 |  |
| Virus infection | HBV | | 145 | 47 | 0.555 |  | | 159 | 66 | 0.419 |
|  | HCV | | 17 | 8 |  |  | | 15 | 4 |  |
|  | HBV+HCV | | 9 | 3 |  |  | | 10 | 5 |  |
|  | none | | 20 | 11 |  |  | | 18 | 3 |  |
| Cirrrhosis | absent | | 48 | 24 | 0.157 |  | | 57 | 19 | 0.552 |
|  | present | | 143 | 45 |  |  | | 145 | 59 |  |
| Child-pugh score | Class A | | 144 | 52 | 1.000 |  | | 170 | 63 | 0.482 |
|  | Class B | | 47 | 17 |  |  | | 32 | 15 |  |
| Tumor number | single | | 122 | 32 | 0.015 |  | | 161 | 49 | 0.005 |
|  | multiple | | 69 | 37 |  |  | | 41 | 29 |  |
| Maximal tumor size | ≤5cm | | 80 | 28 | 0.887 |  | | 131 | 27 | <0.001 |
|  | >5cm | | 111 | 41 |  |  | | 71 | 51 |  |
| Tumor encapsulation | absent | | 117 | 28 | 0.004 |  | | 159 | 36 | <0.001 |
|  | present | | 74 | 41 |  |  | | 43 | 42 |  |
| Microvascular invasion | absent | | 115 | 27 | 0.003 |  | | 126 | 27 | <0.001 |
|  | present | | 76 | 42 |  |  | 76 | | 51 |  |
| Tumor differentiation | I-II | | 159 | 47 | 0.015 |  | 163 | | 46 | <0.001 |
|  | III-Ⅳ | | 32 | 22 |  |  | 39 | | 32 |  |
| TNM stage | I-II | | 154 | 44 | 0.008 |  | 169 | | 49 | <0.001 |
|  | III-Ⅳ | | 37 | 25 |  |  | 33 | | 29 |  |

**Table S8**. Correlation between CD163 expression and clinicopathological characteristics of HCC patients

|  |  | Cohort I | | |  |  | Cohort II | | |  |
| --- | --- | --- | --- | --- | --- | --- | --- | --- | --- | --- |
| Clinicopathological variables | | Tumor CD163 expression | | | *P* Value |  | Tumor CD163 expression | | | *P* Value |
|  |  | Negative (n=156) | | Positive (n=104) |  |  | Negative (n=165) | | Positive (n=115) |  |
| Age | | | 52.21 (11.862) | 51.60(10.424) | 0.294 |  | | 51.75(9.961) | 49.91(9.773) | 0.666 |
| Sex | female | | 27 | 22 | 0.518 |  | | 26 | 18 | 1.000 |
|  | male | | 129 | 82 |  |  | | 139 | 97 |  |
| Serum AFP | ≤20ng/ml | | 48 | 19 | 0.030 |  | | 34 | 21 | 0.650 |
|  | >20ng/ml | | 108 | 85 |  |  | | 131 | 94 |  |
| Virus infection | HBV | | 117 | 75 | 0.131 |  | | 126 | 99 | 0.189 |
|  | HCV | | 10 | 15 |  |  | | 13 | 6 |  |
|  | HBV+HCV | | 9 | 3 |  |  | | 12 | 3 |  |
|  | none | | 20 | 11 |  |  | | 14 | 7 |  |
| Cirrrhosis | absent | | 46 | 26 | 0.481 |  | | 47 | 29 | 0.586 |
|  | present | | 110 | 78 |  |  | | 118 | 86 |  |
| Child-pugh score | Class A | | 118 | 78 | 1.000 |  | | 137 | 96 | 1.000 |
|  | Class B | | 38 | 26 |  |  | | 28 | 19 |  |
| Tumor number | single | | 95 | 59 | 0.522 |  | | 134 | 76 | 0.005 |
|  | multiple | | 61 | 45 |  |  | | 31 | 39 |  |
| Maximal tumor size | ≤5cm | | 73 | 35 | 0.040 |  | | 98 | 60 | 0.270 |
|  | >5cm | | 83 | 69 |  |  | | 67 | 55 |  |
| Tumor encapsulation | absent | | 97 | 48 | 0.015 |  | | 129 | 66 | <0.001 |
|  | present | | 59 | 56 |  |  | | 36 | 49 |  |
| Microvascular invasion | absent | | 100 | 42 | <0.001 |  | | 96 | 57 | 0.180 |
|  | present | | 56 | 62 |  |  | 69 | | 58 |  |
| Tumor differentiation | I-II | | 131 | 75 | 0.028 |  | 131 | | 78 | 0.036 |
|  | III-Ⅳ | | 25 | 29 |  |  | 34 | | 37 |  |
| TNM stage | I-II | | 129 | 69 | 0.003 |  | 137 | | 81 | 0.019 |
|  | III-Ⅳ | | 27 | 35 |  |  | 28 | | 34 |  |

**Table S9**. Correlation between CD8 expression and clinicopathological characteristics of HCC patients

|  |  | Cohort I | | |  |  | Cohort II | | |  |
| --- | --- | --- | --- | --- | --- | --- | --- | --- | --- | --- |
| Clinicopathological variables | | Tumor CD8 expression | | | *P* Value |  | Tumor CD8 expression | | | *P* Value |
|  |  | Negative (n=208) | | Positive (n=52) |  |  | Negative (n=221) | | Positive (n=59) |  |
| Age | | | 51.66(11.047) | 53.19(12.259) | 0.624 |  | | 50.58(10.117) | 52.54(8.993) | 0.282 |
| Sex | female | | 37 | 12 | 0.428 |  | | 34 | 10 | 0.840 |
|  | male | | 171 | 40 |  |  | | 187 | 49 |  |
| Serum AFP | ≤20ng/ml | | 51 | 16 | 0.378 |  | | 45 | 10 | 0.712 |
|  | >20ng/ml | | 157 | 36 |  |  | | 176 | 49 |  |
| Virus infection | HBV | | 149 | 43 | 0.268 |  | | 181 | 44 | 0.303 |
|  | HCV | | 22 | 3 |  |  | | 15 | 4 |  |
|  | HBV+HCV | | 9 | 3 |  |  | | 9 | 6 |  |
|  | none | | 28 | 3 |  |  | | 16 | 5 |  |
| Cirrrhosis | absent | | 60 | 12 | 0.490 |  | | 58 | 18 | 0.514 |
|  | present | | 148 | 40 |  |  | | 163 | 41 |  |
| Child-pugh score | Class A | | 162 | 34 | 0.072 |  | | 183 | 50 | 0.846 |
|  | Class B | | 46 | 18 |  |  | | 38 | 9 |  |
| Tumor number | single | | 125 | 29 | 0.637 |  | | 154 | 56 | <0.001 |
|  | multiple | | 83 | 23 |  |  | | 67 | 3 |  |
| Maximal tumor size | ≤5cm | | 78 | 30 | 0.011 |  | | 113 | 45 | 0.001 |
|  | >5cm | | 130 | 22 |  |  | | 108 | 14 |  |
| Tumor encapsulation | absent | | 106 | 39 | 0.002 |  | | 142 | 53 | <0.001 |
|  | present | | 102 | 13 |  |  | | 79 | 6 |  |
| Microvascular invasion | absent | | 100 | 42 | <0.001 |  | | 110 | 43 | 0.002 |
|  | present | | 108 | 10 |  |  | 111 | | 16 |  |
| Tumor differentiation | I-II | | 163 | 43 | 0.570 |  | 157 | | 52 | 0.007 |
|  | III-Ⅳ | | 45 | 9 |  |  | 64 | | 7 |  |
| TNM stage | I-II | | 152 | 46 | 0.019 |  | 164 | | 54 | 0.004 |
|  | III-Ⅳ | | 56 | 6 |  |  | 57 | | 5 |  |

**Table S10**. Univariate and multivariate analysis of factors associated with survival and recurrence in two independent cohorts of human HCC

| Clinical Variables | Time To Recurrence | |  | Overall Survival | |
| --- | --- | --- | --- | --- | --- |
|  | HR( 95% CI ) | P value |  | HR(95% CI) | P value |
| **Univariate analysis** |  |  |  |  |  |
| **Cohort I(n=260)** |  |  |  |  |  |
| Age (≤50 versus > 50) | 0.982(0.969-0.995) | 0.006 |  | 0.981(0.968-0.994) | 0.005 |
| Sex (female versus male) | 1.081(0.735-1.589) | 0.692 |  | 1.145(0.778-1.686) | 0.492 |
| Serum AFP (≤20 versus >20 ng/ml) | 0.741(0.518-1.061) | 0.102 |  | 0.712(0.491-1.031) | 0.072 |
| HBV infection (no versus yes) | 1.263(0.899-1.773) | 0.178 |  | 1.304(0.925-1.840) | 0.130 |
| Cirrhosis (absent versus present) | 1.107(0.794-1.545) | 0.548 |  | 1.141(0.815-1.599) | 0.442 |
| Child-pugh score (A versus B) | 1.042(0.733-1.482) | 0.818 |  | 1.055(0.736-1.513) | 0.769 |
| Tumor number (single versus multiple) | 0.510(0.376-0.691) | <0.001 |  | 0.488(0.358-0.665) | <0.001 |
| Maximal tumor size (≤5cm versus >5) | 0.662(0.484-0.907) | 0.010 |  | 0.636(0.460-0.878) | 0.006 |
| tumor encapsulation (present versus absent) | 0.292(0.214-0.399) | <0.001 |  | 0.279(0.203-0.384) | <0.001 |
| Microvascular invasion (absent versus present) | 0.321(0.236-0.439) | <0.001 |  | 0.302(0.219-0.415) | <0.001 |
| Tumor differentiation (I-II versus III-Ⅳ) | 0.317(0.225-0.446) | <0.001 |  | 0.297(0.211-0.419) | <0.001 |
| TNM stage (I-II versus III) | 0.150(0.106-0.211) | <0.001 |  | 0.141(0.100-0.199) | <0.001 |
| SOX12 expression (negative versus positive) | 0.564(0.414-0.769) | <0.001 |  | 0.540(0.393-0.740) | <0.001 |
| **Multivariate analysis** |  |  |  |  |  |
| Tumor number (single versus multiple) | 0.741(0.476-1.154) | 0.185 |  | 0.704(0.447-1.107) | 0.128 |
| Maximal tumor size (≤5cm versus >5) | 0.739(0.503-1.087) | 0.124 |  | 0.721(0.484-1.074) | 0.108 |
| Tumor encapsulation (present versus absent) | 0.747(0.469-1.191) | 0.221 |  | 0.785(0.486-1.266) | 0.320 |
| Microvascular invasion (absent versus present) | 0.569(0.369-0.878) | 0.011 |  | 0.523(0.336-0.812) | 0.004 |
| Tumor differentiation (I-II versus III-Ⅳ) | 0.593(0.406-0.865) | 0.007 |  | 0.561(0.384-0.821) | 0.003 |
| TNM stage (I-II versus III) | 0.293(0.175-0.490) | <0.001 |  | 0.295(0.174-0.499) | <0.001 |
| SOX12 expression (negative versus positive) | 1.151(0.805-1.644) | 0.441 |  | 1.119(0.778-1.610) | 0.543 |
|  |  |  |  |  |  |
| **Cohort II(n=280)** |  |  |  |  |  |
| Age (≤50 versus > 50) | 0.992(0.977-1.008) | 0.327 |  | 0.988(0.972-1.003) | 0.117 |
| Sex (female versus male) | 0.926(0.596-1.439) | 0.732 |  | 1.013(0.662-1.550) | 0.952 |
| Serum AFP (≤20 versus >20 ng/ml) | 1.052(0.726-1.523) | 0.789 |  | 1.103(0.768-1.584) | 0.595 |
| HBV infection (no versus yes) | 0.454(0.284-0.725) | 0.001 |  | 0.434(0.272-0.692) | <0.001 |
| Cirrhosis (absent versus present) | 0.825(0.583-1.168) | 0.278 |  | 0.805(0.570-1.139) | 0.221 |
| Child-pugh score (A versus B) | 0.736(0.500-1.083) | 0.120 |  | 0.747(0.508-1.097) | 0.137 |
| Tumor number (single versus multiple) | 0.417(0.300-0.579) | <0.001 |  | 0.418(0.302-0.580) | <0.001 |
| Maximal tumor size (≤5cm versus >5) | 0.398(0.292-0.544) | <0.001 |  | 0.432(0.317-0.587) | <0.001 |
| Tumor encapsulation (present versus absent) | 0.327(0.239-0.447) | <0.001 |  | 0.345(0.253-0.470) | <0.001 |
| Microvascular invasion (absent versus present) | 0.452(0.332-0.616) | <0.001 |  | 0.441(0.324-0.599) | <0.001 |
| Tumor differentiation (I-II versus III-Ⅳ) | 0.283(0.204-0.391) | <0.001 |  | 0.290(0.211-0.400) | <0.001 |
| TNM stage (I-II versus III) | 0.182(0.130-0.256) | <0.001 |  | 0.170(0.122-0.238) | <0.001 |
| SOX12 expression (negative versus positive) | 0.459(0.330-0.638) | <0.001 |  | 0.411(0.295-0.573) | <0.001 |
| **Multivariate analysis** |  |  |  |  |  |
| Tumor number (single versus multiple) | 0.789(0.527-1.181) | 0.249 |  | 0.818(0.542-1.233) | 0.336 |
| Maximal tumor size (≤5cm versus >5) | 0.773(0.536-1.115) | 0.169 |  | 0.872(0.608-1.249) | 0.454 |
| Tumor encapsulation (present versus absent) | 0.746(0.492-1.132) | 0.168 |  | 0.806(0.533-1.221) | 0.310 |
| Microvascular invasion (absent versus present) | 0.692(0.464-1.032) | 0.071 |  | 0.626(0.422-0.929) | 0.020 |
| Tumor differentiation (I-II versus III-Ⅳ) | 0.538(0.374-0.773) | 0.001 |  | 0.550(0.385-0.786) | 0.001 |
| TNM stage (I-II versus III) | 0.350(0.221-0.552) | <0.001 |  | 0.304(0.193-0.481) | <0.001 |
| SOX12 expression (negative versus positive) | 0.630(0.445-0.892) | 0.009 |  | 0.567(0.399-0.804) | 0.001 |

**Table S11**. Primer sequences used in the study

| Primer name | Primer sequences | Enzyme |
| --- | --- | --- |
| Primers for real-time PCR: |  |  |
| Human SOX12 sense: | 5'-CGCGATGGTGCAGCAGCG-3' |  |
| Human SOX12 antisense: | 5'-GCCACTGGTCCATGATCTTC-3' |  |
| Mouse SOX12 sense: | 5'-GGAGACGGTGGTATCTGGG-3' |  |
| Mouse SOX12 antisense: | 5'-ATCATCTCGGTAACCTCGGGG-3' |  |
| Human CCL22 sense: | 5'-ATTACGTCCGTTACCGTCTGC-3' |  |
| Human CCL22 antisense: | 5'-TCCCTGAAGGTTAGCAACACC-3' |  |
| Human CD274 sense: | 5'-GGACAAGCAGTGACCATCAAG-3' |  |
| Human CD274 antisense: | 5'-CCCAGAATTACCAAGTGAGTCCT-3' |  |
| Mouse CCR4 sense: | 5'-GGAAGGTATCAAGGCATTTGGG-3' |  |
| Mouse CCR4 antisense: | 5'-GTACACGTCCGTCATGGACTT-3' |  |
| Mouse IL-10 sense: | 5'-CAGAGCCACATGCTCCTAGA-3' |  |
| Mouse IL-10 antisense: | 5'-TGTCCAGCTGGTCCTTTGTT-3' |  |
| Mouse IL-35 sense: | 5'-TCAGAATCACAACCATCA-3' |  |
| Mouse IL-35 antisense: | 5'-CGCCATTATGATTCAGAGACTG-3' |  |
| Mouse TGF-β1 sense: | 5'-AAACTAAGGCTCGCCAGTCC-3' |  |
| Mouse TGF-β1 antisense: | 5'-TTGTTGCGGTCCACCATT-3' |  |
| Human β-actin sense: | 5'-CATGTACGTTGCTATCCAGGC-3' |  |
| Human β-actin antisense: | 5'-CTCCTTAATGTCACGCACGAT-3' |  |
| Mouse GAPDH sense: | 5'-AGGTCGGTGTGAACGGATTTG-3' |  |
| Mouse GAPDH antisense: | 5'-TGTAGACCATGTAGTTGAGGTCA-3' |  |
|  |  |  |
| Primers for *CCL22* promoter construct: | | |
| (-1127/+148) CCL22 sense: | 5’-TATAGGTACCTTGAGCCCAGGAGGTCAA-3’ | KpnI |
| (-454/+148) CCL22 sense: | 5’-TATAGGTACCCTGCCTGGGCCTTCCAAA-3’ | KpnI |
| (-315/+148) CCL22 sense: | 5’-TATAGGTACCTAGAGCTTGGAGTCTGAC-3’ | KpnI |
| Antisense: | 5’-ATATAAGCTTTCCACCTCAGGCTGGGAT-3’ | HindIII |
| Primers for *CCL22* promoter site-directed mutagenesis: | | |
| SOX12 binding site: |  |  |
| binding site 1 mutation sense: | 5’-TCCTGCCTCTAAA**gtgg**CGACAACAACAAC-3’ |  |
| binding site 1 mutation antisense: | 5’-GTTGTTGTTGTCG**ccac**TTTAGAGGCAGGA-3’ |  |
| binding site 2 mutation sense: | 5’-TAAAACAACGACA**gtgg**CAACAAAAATCCT -3’ |  |
| binding site 2 mutation antisense: | 5’-AGGATTTTTGTTG**ccac**TGTCGTTGTTTTA-3’ |  |
| binding site 3 mutation sense: | 5’-GTGTTCTGAGCAC**ttgg**CATGTGCCTGATC-3’ |  |
| binding site 3 mutation antisense: | 5’-GATCAGGCACATG**ccaa**GTGCTCAGAACAC-3’ |  |
| Primers used for ChIP in the *CCL22* promoter: | | |
| distant region sense: | 5’-AAGTGATCTGCCTGCCTC-3’ |  |
| distant region antisense: | 5’-TATGTGCCAAGTGCACTG-3’ |  |
| binding site sense: | 5’-TGTGAACTGGGCAAGTCC-3’ |  |
| binding site antisense: | 5’-TTCCCCTGAGGGATCACA-3’ |  |
|  |  |  |
| Primers for *CD274* promoter construct: | | |
| (-1972/+156) CD274 sense: | 5’-TATAGGTACCTTCAATCTCCGGGTAGTT-3’ | KpnI |
| (-1677/+156) CD274 sense: | 5’-TATAGGTACCTGATTTAATCCTGGCTAC-3’ | KpnI |
| (-1055/+156) CD274 sense: | 5’-TATAGGTACCAATGCAAGGGCTATCTCA-3’ | KpnI |
| (-908/+156) CD274 sense: | 5’-TATAGGTACCGGCTCAAGCCTGTAATAT-3’ | KpnI |
| Antisense: | 5’-ATATAAGCTTATCCCAAAGAAAGGGTGT-3’ | HindIII |
| Primers for *CD274* promoter site-directed mutagenesis: | |  |
| SOX12 binding site: |  |  |
| binding site 1 mutation sense: | 5’-GGTGGGAAAGATG**ggtg**AAAACACGAATCC-3’ |  |
| binding site 1 mutation antisense: | 5’-GGATTCGTGTTTT**cacc**CATCTTTCCCACC-3’ |  |
| binding site 2 mutation sense: | 5’-GCTCTGAAGCCAG**ccac**TTTTTTTTGTTTT-3’ |  |
| binding site 2 mutation antisense: | 5’-AAAACAAAAAAAA**gtgg**CTGGCTTCAGAGC-3’ |  |
| binding site 3 mutation sense: | 5’-GAAGGAAAGGCAA**gtgg**CGAAGAGTCCAAT-3’ |  |
| binding site 3 mutation antisense: | 5’-ATTGGACTCTTCG**ccac**TTGCCTTTCCTTC-3’ |  |
| Primers used for ChIP in the *CD274* promoter: | | |
| distant region sense: | 5’-CTCCGGGTAGTTGATCAA-3’ |  |
| distant region antisense: | 5’-ACTAGCTGAGTTAACTTG-3’ |  |
| binding site sense: | 5’-TTTGGGAAGTCACCCAAA-3’ |  |
| binding site antisense: | 5’-GTTCCTCGACATAATGAA-3’ |  |
|  |  |  |
| Primers for *SOX12* promoter construct: | | |
| (-3041/+198) SOX12 sense: | 5’-TATAGAGCTCAATGTCCTCTCTTCCAGG-3’ | SacI |
| (-2901/+198) SOX12 sense: | 5’-TATAGAGCTCAAGTCCTGGTGTCTACAC-3’ | SacI |
| (-824/+198) SOX12 sense: | 5’-TATAGAGCTCAGCTCAGGACCTTTGGCT-3’ | SacI |
| (-108/+198) SOX12 sense: | 5’-TATAGAGCTCGGCACCTCGGTGTTTACA-3’ | SacI |
| (-3/+198) SOX12 sense: | 5’-TATAGAGCTCGACACTGAAAGGCGTCGG-3’ | SacI |
| Antisense: | 5’-ATATAAGCTTAGAAGGTCAGCGCGGTCT-3’ | HindIII |
| Primers for *SOX12* promoter site-directed mutagenesis: | | |
| Smad2/3/4 binding site: |  |  |
| binding site 4 mutation sense: | 5’-CACTGCATAAATG**ctc**GTTGAGGAAGAAA-3’ |  |
| binding site 4 mutation antisense: | 5’-TTTCTTCCTCAAC**gag**CATTTATGCAGTG-3’ |  |
| binding site 3 mutation sense: | 5’-CCTAGGTTCAGCC**gag**CTTTAACCATCTC-3’ |  |
| binding site 3 mutation antisense: | 5’-GAGATGGTTAAAG**ctc**GGCTGAACCTAGG-3’ |  |
| binding site 2 mutation sense: | 5’-AATCGAATCCTAG**ctc**GCGTGTCCTCGGT-3’ |  |
| binding site 2 mutation antisense: | 5’-ACCGAGGACACGC**gag**CTAGGATTCGATT-3’ |  |
| binding site 1 mutation sense: | 5’-CGCAGCGGCCCGC**gag**CGGCGAGGGGGAG-3’ |  |
| binding site 1 mutation antisense: | 5’-CTCCCCCTCGCCG**ctc**GCGGGCCGCTGCG-3’ |  |
| Primers used for ChIP in the *SOX12* promoter: | | |
| distant region sense: | 5’-GCCCCAGTTCCAAGTTCA-3’ |  |
| distant region antisense: | 5’-AGGCCCTGGAAGAACCTT-3’ |  |
| binding site sense: | 5’-ACGGCAGGTGTCTCGGAA-3’ |  |
| binding site antisense: | 5’-AGAAGGTCAGCGCGGTCT-3’ |  |
|  |  |  |
| Primers for genome sequencing of genetically modified mice: | | |
| *Sox12*^fl/fl^ forward | 5’-CGGTCTGCCCTTGTGAGAAATG-3’ | |
| *Sox12*^fl/fl^ reverse | 5’-TACACATGCAGAACCACAGGGC-3’ | |
| *Sox12*^fl-stop-fl^ WT forward | 5’-CACTTGCTCTCCCAAAGTCGCTC-3’ | |
| *Sox12*^fl-stop-fl^ flox forward | 5’-GCATCTGACTTCTGGCTAATAAAG-3 | |
| *Sox12*^fl-stop-fl^ reverse | 5’-ATACTCCGAGGCGGATCACAA-3’ | |
| *Ccr4*^fl/fl^ forward | 5’-GTAGGGGTGATGGTTCAGTGG-3’ | |
| *Ccr4*^fl/fl^ reverse | 5’-CTGTGTGTGCGGTCATTCAGC-3’ | |
| *Alb-cre* forward | 5’-GAAGCAGAAGCTTAGGAAGATGG-3’ | |
| *Alb-cre* reverse | 5’-TTGGCCCCTTACCATAACTG-3’ | |
| *Foxp3*^cre^ forward | 5’-AGCAAGTGAGGTGCTGGACATG-3’ | |
| *Foxp3*^cre^ reverse | 5’-CTGCACACAGACAGGAGCATCTTC-3’ | |

**Table S12**. Gene knockdown sequences used in this study

| Gene name | Sequence (5’-3’) |
| --- | --- |
| Mouse shSOX12 | CCGG**GCTACAGCAGAGGAGGGCGAA**CTCGAGTTCGCCCTCCTCTGCTGTAGCTTTTTG |
| Mouse shCCL22 | CCGG**GCCATCACGTTTAGTGAAGGA**CTCGAGTCCTTCACTAAACGTGATGGCTTTTTG |
| Human shCCL22 | CCGG**GCGTGGTGAAACACTTCTACT**CTCGAGAGTAGAAGTGTTTCACCACGCTTTTTG |
| Mouse shPD-L1 | CCGG**CCGAAATGATACACAATTCGA**CTCGAGTCGAATTGTGTATCATTTCGGTTTTTG |
| Human shSmad2 | CCGG**CAAGTACTCCTTGCTGGATTG**CTCGAGCAATCCAGCAAGGAGTACTTGTTTTTG |
| Human shSmad3 | CCGG**GAGCCTGGTCAAGAAACTCAA**CTCGAGTTGAGTTTCTTGACCAGGCTCTTTTTG |
| Human shSmad4 | CCGG**GTACTTCATACCATGCCGATT**CTCGAGAATCGGCATGGTATGAAGTACTTTTTG |
